# Partial Enteral Nutrition in the Management of Crohn’s Disease: A Systematic Review and Meta-Analysis

**DOI:** 10.1093/ecco-jcc/jjae177

**Published:** 2024-11-20

**Authors:** Aleksandra Jatkowska, Bernadette White, Konstantinos Gkikas, John Paul Seenan, Jonathan MacDonald, Konstantinos Gerasimidis

**Affiliations:** Human Nutrition, School of Medicine, Dentistry and Nursing, University of Glasgow, Glasgow, UK; Human Nutrition, School of Medicine, Dentistry and Nursing, University of Glasgow, Glasgow, UK; Human Nutrition, School of Medicine, Dentistry and Nursing, University of Glasgow, Glasgow, UK; Human Nutrition, School of Medicine, Dentistry and Nursing, University of Glasgow, Glasgow, UK; Department of Gastroenterology, Queen Elizabeth University Hospital, Glasgow, UK; Human Nutrition, School of Medicine, Dentistry and Nursing, University of Glasgow, Glasgow, UK; Department of Gastroenterology, Queen Elizabeth University Hospital, Glasgow, UK; Human Nutrition, School of Medicine, Dentistry and Nursing, University of Glasgow, Glasgow, UK

**Keywords:** Enteral nutrition, Crohn's Disease, nutritional therapy, biologics

## Abstract

**Background:**

Exclusive enteral nutrition is an established treatment for active Crohn’s disease but the role of partial enteral nutrition (PEN) in the broader management of the disease is less clear. This systematic review and meta-analysis reviewed the literature on the role of PEN in Crohn’s disease management.

**Methods:**

This review was conducted following Cochrane recommendations. The protocol was registered on PROSPERO. Findings were reported following the PRISMA guidelines.

**Results:**

Sixty-four articles were identified, of which 11 reported data from randomized control trials. Good quality evidence suggests that PEN may be used as a maintenance and induction therapy, particularly at high dosages and/or alongside exclusion diets. A higher dosage of PEN is associated with a lower risk of subsequent disease relapse, with benefits observed at intakes above 35% of energy requirements (35%-50% PEN: OR [95% confidence intervals (CI)]: 0.42 [0.27-0.65]; > 50% PEN: OR [95% CI]: 0.27 [0.08-0.88]). Low-quality evidence suggests that postoperative use of PEN may prevent disease recurrence or enhance treatment outcomes when used as adjunct therapy to biologics. PEN can improve nutritional parameters, showing efficacy comparable to EEN in pediatric patients (weight: OR [95% CI]: −0.04 [−0.32, 0.25]). The effect of PEN on improving patients’ quality of life is comparable to that of EEN and anti-tumor necrosis factor alpha therapies.

**Conclusions:**

Partial enteral nutrition may help in various aspects of Crohn’s disease management but much of the current evidence is of low quality. Well-designed randomized control trials are required to confirm findings from current literature and before clinical recommendations can be made.

## 1. Introduction

In Crohn’s disease (CD), mainstream induction treatments like corticosteroids and advanced therapies are effective but their use is often associated with significant side effects,^1^ modest primary response rates,^2^ or subsequent loss of response (LOR).^3^ Exclusive enteral nutrition (EEN), commonly used in the management of pediatric active CD, involves the exclusive consumption of a proprietary nutritional formula for 6-12 weeks.^4^ In children, EEN demonstrates efficacy rates comparable to oral corticosteroids but with a superior safety profile and the additional benefits of nutritional rehabilitation and mucosal healing.^5^ Despite its effectiveness, EEN is less commonly used in adults due to palatability and the impact of treatment on lifestyle.^6^

Partial enteral nutrition (PEN) is often used as maintenance treatment, involving the replacement of a portion of a person’s habitual diet with the same proprietary formula used in EEN, but with the flexibility of incorporating regular table foods. Societal guidelines from the European Crohn’s and Colitis Organisation and the European Society of Paediatric Gastroenterology Hepatology and Nutrition endorse PEN as an effective maintenance treatment for CD if provided in appropriate dosages.^7^ However, the exact dosage of PEN needed to be effective remains to be established. Previous and recent research has also explored the efficacy of PEN as a sole or adjunct induction therapy alongside unrestricted diet, conventional medical treatments, and/or coupled with dietary modification.^8–10^

The present systematic review and meta-analysis investigated the role of PEN in the broader management of CD. We evaluated the efficacy of PEN either as a stand-alone therapy or in combination with other treatments in induction of remission in active disease and maintenance of remission in patients of all ages. Additionally, we assessed the effect of PEN on secondary outcomes, including nutritional status parameters, quality of life, and other relevant factors identified during the literature search.

## 2. Methods

The review protocol was registered on the international database of prospectively registered systematic reviews (PROSPERO, protocol ID: CRD42021239325), and the findings were reported following the preferred reporting items for systematic reviews and meta-analyses guidelines.^11^

### 2.1. Eligibility criteria

Eligible studies included randomized control trials (RCTs), non-RCTs, prospective observational, retrospective, cross-sectional, and case-control studies, which used PEN in CD either as a stand-alone treatment or in conjunction with other drug therapies or exclusion diets. Studies including patients with active CD and those in remission were eligible. There were no age or other demographic restrictions, and no limitations on the duration or dosage of PEN. Exclusion criteria included patients with inflammatory bowel disease (IBD) subtypes other than CD, studies reporting combined results for different IBD subtypes, non-English studies, and research in animals or in vitro. Treatment groups for comparisons with PEN comprised drug therapies, dietary therapies, and unrestricted diet. Studies without a comparator group were also eligible. All eligible studies, meeting the above criteria and evaluating at least one of the following efficacy outcomes, were included: Clinical, endoscopic, histological, radiological, postoperative remission/relapse rates, systemic and intestinal inflammatory biomarkers, nutritional status parameters, quality of life, healthcare costs, and hospitalization rates.

For the purposes of this review, supplementary PEN is defined as PEN provided in addition to a patient’s usual diet and is a modality more commonly used in patients with malnutrition. In contrast, PEN that replaces a person’s habitual diet is used more commonly as a disease-modifying therapeutic strategy, with its dosage often expressed as a percentage of total energy requirements or intake.

### 2.2. Search strategy

PubMed, Ovid Embase, Cochrane Controlled Register of Trials, and Cumulative Index to Nursing and Allied Health Literature databases were searched in March 2021, and the search was repeated again in November 2023 prior to finalizing the manuscript for submission. Reference lists of identified articles were screened for additional studies. The search strategy used the following keywords:

1. Crohn* [Title/Abstract]
2. Enteral Nutrition
3. Enteral Feeding
4. Diet
5. Diets
6. Nutrition* Therap*
7. Diet* Therap*
8. Elemental Diet
9. Enteric Feeding
10. Liquid Diet
11. Tube Feeding
12. 2 OR 3 OR 4 OR 5 OR 6 OR 7 OR 8 OR 9 OR 10 OR 11
13. 1 AND 12

### 2.3. Data collection

Two reviewers (AJ and BW) independently conducted database searches, removing duplicates with EndNote 20. Titles, abstracts, and full texts of articles were independently screened by the same 2 reviewers according to predetermined eligibility criteria. In case of disagreement, a third senior reviewer (KG) independently assessed full texts to reach a consensus.

The methodological quality of the included studies was evaluated by the 2 reviewers using the Critical Appraisal Skills Programme (CASP) tool.^12,13^ In instances of disagreement, the third senior reviewer was consulted. An evidence table was created to provide an overview of the included studies and report on the risk of bias. Indicators of quality of evidence for each subtheme included an RCT study design, inclusion of a comparator group, assessment of study bias, compliance assessment, and reporting of objective outcome measures.

### 2.4. Meta-analysis

Meta-analysis was conducted using RevMan5,^14^ pooling data from studies assessing similar outcomes and comparing PEN against similar comparator groups. Data were analyzed with random-effects meta-analysis, considering anticipated heterogeneity in the included studies. Dichotomous data were analyzed using the Mantel–Haenszel method to calculate odds ratios (ORs). Continuous data were analyzed with mean differences when studies used the same outcome measure, or standardized mean differences when different outcome measures were reported. In cases where mean or standard deviation (SD) values were not provided, these were imputed from other available descriptive statistics using the equations and estimations recommended by the Cochrane Handbook (https://training.cochrane.org/handbook/current). Likewise, changes from baseline and post-treatment values were combined into a single analysis following the same recommendations. To stratify studies by PEN dosage, values expressed in kcal/day were converted to a percentage of the recommended daily intake, assuming an average daily energy requirement of 2000 kcal/day. In instances where the energy content of the formula was not specified, a standard energy content of 1 kcal/mL was assumed.

## 3. Results

**Figure 1:**
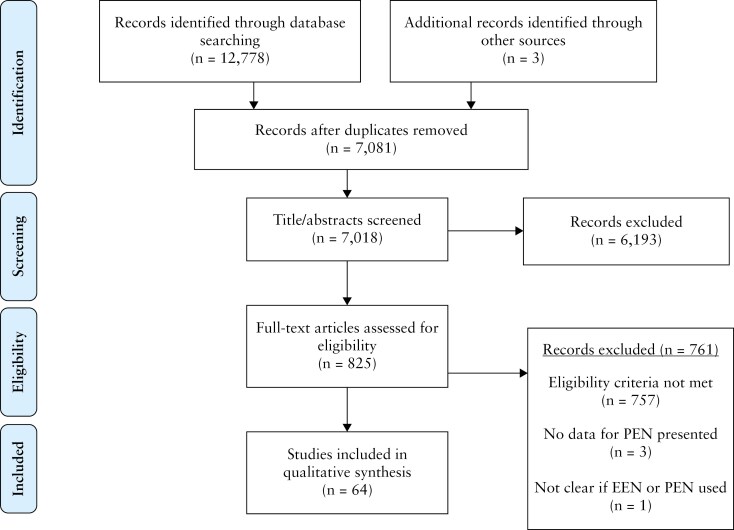
PRISMA flow diagram of the review process. PRISMA, preferred reporting items for systematic reviews and meta-analyses.

Figure 1 describes the screening process. A total of 12,778 records were identified, with an additional 3 articles^15–17^ identified through screening reference lists. From the excluded records, 10 initially showed discrepancies between the 2 reviewers, prompting further evaluation by a third, independent reviewer (Supplementary Table 1). Sixty-four articles were included in the final review.

Most articles originated from Japan (25 of 64, 39%), followed by the United Kingdom (8 of 64, 13%). Almost half of the included articles (30 of 64, 47%) used PEN in patients with CD in remission, while 39% (25 of 64) in those with active disease. Among the latter, most articles used clinical disease activity indices or a combination of clinical, biochemical, and endoscopic criteria to define active disease. Two recent studies used fecal calprotectin (FC) values to define the active disease.^18,19^ In 3 articles, disease status was unclear, and another 6 included patients with both active disease and those in remission. In 2 of these 6 studies, the majority of patients had active disease, and these studies were included in the theme focusing on the use of PEN in active CD,^20,21^ while one mostly included patients in remission^22^ and was included in the theme looking at the use of PEN as maintenance treatment. Notably, 56% of the articles (36 of 64) included adult patients, 39% included children and adolescents (25 of 64), 3% included both adults and children, and 2% did not specify patient age. The use of polymeric formulas was the most common (27 of 64, 42%), followed by elemental formulas (21 of 64, 33%).

Compliance with PEN was assessed in most articles (41 of 64, 64%), primarily using self-reported measures and conventional dietary assessment methodology, with a single study objectively assessing compliance to PEN formula enriched with fish oil by measuring plasma phospholipids.^23^ Sixty-three percent of articles (40 of 64) had a comparator group, 20% did not have a comparator group (13 of 64), and 17% categorized patients into PEN and non-PEN groups based on PEN intakes (11 of 64, 17%). However, as both groups included patients receiving PEN, they were treated as 2 PEN groups. Almost half (29 of 64, 45%) were of retrospective study design and 13 reported findings from 11 distinct RCTs. Detailed information and study characteristics of the 64 articles are presented in Supplementary Table 2.

Following the literature search, 4 primary themes emerged, each with subthemes, and each subtheme included at least 3 articles: (1) PEN as induction treatment, (2) PEN as maintenance treatment, (3) PEN and nutritional outcomes, and (4) PEN and quality of life. A fifth theme explored additional outcomes, which could not form their own themes due to scarce literature or a limited number of available studies. The sixth and final theme explored associations between residual dietary intake and disease outcomes during PEN.

### 3.1. PEN as induction treatment

Twenty-seven articles investigated the role of PEN in the treatment of active CD,^8–10,18–21,23–43^ and were categorized into 3 subthemes depending on the type of concomitant medical or dietary treatments used.

#### 3.1.1. PEN alongside unrestricted diet

Twelve articles (12 of 27, 44%) used PEN alongside an unrestricted diet in patients with active CD (Supplementary Table 3).^8,20,23,26,28–31,33–35,42^ Of these, 6 studies included children,^8,20,26,29,30,34^ and the other 6 included adults.^23,28,31,33,35,42^ One study was an RCT,^8^ and another one a pragmatic RCT.^28^ Eight articles reported clinical remission rates,^8,20,26,28,30,31,34,42^ 9 reported changes in systemic inflammatory biomarkers,^8,20,23,26,31,33–35,42^ 2 in FC,^30,33^ and 1 study described endoscopic findings.^35^

Clinical remission rates varied widely across studies, ranging from 15% to 72%, but with the majority (6 of 8, 75%) reporting ≥ 50% remission rates.^20,26,30,31,34,42^ The lowest remission rate (15%) was observed in an RCT involving pediatric patients, where 50% PEN was less effective than EEN (42% remission rate) after 6 weeks of both (*p* = 0.035).^8^ Low efficacy rates, including patients receiving EEN, may be attributed to high dropout rates, poor compliance likely due to the use of less palatable elemental formula, and overcompensation of the habitual diet (median energy intake: 126% of estimated average requirements [EARs]).^8^ Conversely, the highest remission rate of 72% was reported in a study involving malnourished adults who received 12 weeks of supplementary PEN at 200-600 kcal/day, alongside initiation of medical induction therapy with corticosteroids or biologics; hence, the net effect of PEN on remission rates could not be determined.^42^ The second-highest remission rate of 68% was reported in a study using the highest dosage of PEN (~1500 kcal/day).^31^ Of note, 1 retrospective study observed a higher remission rate of 57% with 35%-50% PEN using the Modulen IBD formula, compared to 22% with the same dosage and duration of PEN but using the Ensure Plus formula (*p* = 0.03).^20^ Nonetheless, it is important to note that the retrospective study design may have introduced sample selection bias.

Regarding the effect of treatment with PEN on systemic inflammatory biomarkers, the majority of articles (6 of 9, 67%) reported improvements.^20,26,31,34,35,42^ Among these 6 articles, 2 provided different PEN formulas and observed improvements only in patients receiving the Modulen IBD formula, but not in those receiving the Ensure Plus^20^ or Nutren formulas.^35^ It is noteworthy that 2 of 3 studies that did not observe improvements in systemic inflammatory biomarkers involved patients with normal C-reactive protein (CRP) levels at PEN initiation.^23,33^ In the only RCT available, improvements in ESR, albumin, platelet count, and hemoglobin were observed only with EEN but not with PEN, yet neither treatment improved CRP levels.^8^ In a study involving malnourished adults receiving supplementary PEN for purposes of nutritional rehabilitation, only those who received higher PEN dosages and were compliant with the treatment had improvements in albumin levels.^42^

In an observational study, 8 weeks of 50% PEN reduced FC levels in pediatric patients.^30^ However, only 14% of patients achieved FC levels < 250 µg/g, which was lower compared to both treatment with EEN (45%, *p* = 0.04) and anti-tumor necrosis factor alpha (TNFα) agents (62%, *p* = 0.02). In another open-label controlled trial, 6 weeks of PEN did not further reduce FC levels in adults after the initial reduction observed with 2 weeks of EEN. This trend was also noted in the parallel group that continued EEN, suggesting a possible decline in treatment compliance over time.^33^ At treatment completion, 22% of patients in the PEN group and 36% in the EEN group had FC levels below 500 µg/g.

A single open-label controlled trial reported both endoscopic and histological findings.^35^ The authors of this study reported that patients receiving 3 months of 50% PEN with the Modulen IBD formula experienced significant histological improvements (*p* < 0.01). In contrast, patients using the Nutren formula did not show similar histological improvements. Regarding endoscopic scoring, none of the groups demonstrated improvements. Importantly, both groups increased their residual energy intake from their habitual diet and reported consuming 30-35% of their daily energy intake from PEN, below the prescribed target of 50%.

Collectively, there is poor-quality evidence to suggest that PEN used alongside unrestricted diet may improve disease activity outcomes in patients with active CD.

#### 3.1.2. PEN alongside exclusion diets

Twelve out of the 27 articles (44%) used PEN in combination with exclusion diets in patients with active CD^10,18,19,21,27,32,36–40,43^ (Table 1). The majority of these studies (7 of 12, 58%) reported results from studies including children,^10,18,19,36–38,43^ 3 included adults,^21,39,40^ and 2 included both age groups.^27,32^ Notably, 5 articles (5 of 12, 42%), including 3^10,36,40^ reporting findings from 2 distinct RCTs, were affiliated with the research group that developed the Crohn’s disease exclusion diet (CDED).^10,27,32,36,40^ In short, CDED limits processed foods, food additives, animal fat, sugar, dairy products, and gluten, and mandates daily consumption of 5 specific foods alongside PEN. Seven of the remaining studies (7 of 12, 58%), including 1 RCT,^19^ were conducted by independent research groups assessing the effect of PEN coupled with CDED or other exclusion diets resembling CDED,^18,19,21,37–39,43^ with 2 of these studies presenting combined results for patients receiving PEN alongside CDED and those on CDED alone.^21,40^ Remission rates and changes in systemic inflammatory biomarkers were reported in all 12 articles in this subtheme. Eight studies measured FC levels,^10,18,19,21,37,39,40,43^ while 2 described endoscopic findings.^37,40^

**Table 1:** Summary of study results investigating the clinical efficacy of partial enteral nutrition alongside exclusion diets as induction treatment in patients with active Crohn’s disease.

Study reference	Intervention description	Comparison description	Duration^a^	Response/remission criteria	Results
**Sigall Boneh et al.^27^** Retrospective	50% PEN + CDED for first 6 weeks, 25% PEN + CDED for another 6 weeks (*n* = 40) CDED for 12 weeks (*n* = 7)	✘	12 weeks	Response: ↓PCDAI*≥12.5/ ↓HBI ≥ 2/ remission Remission: HBI≤3/PCDAI*<7.5 CRP normalization: CRP ≤ 0.5 mg/dL *PCDAI without height component	Combined results for PEN + CDED and CDED groups unless stated otherwise. *Response rates*: **75% (30/40): 75.9% (22/29)** children, **72.7% (8/11)** adults, PEN + CDED group, 100% (7/7) CDED group after 6 weeks *Remission rates*: **67.5% (27/40): 72.4% (21/29)** children, **54.5% (6/11)** adults, PEN + CDED group, 85.7% (6/7): 75% (3/4) children, 100% (3/3) adults, CDED group after 6 weeks; **84% (27/32)** sustained remission after 12 weeks *HBI mean* (*SD*): ↓from **6.4 (2.7) to 1.9 (2.9)** after 6 weeks (*p* < 0.0001), ↓from **5.9 (2.7) to 0.8 (1.8)** after 12 weeks (*p* < 0.0001) *PCDAI mean* (*SD*): ↓from **27.7 (9.4) to 5.4 (8.0)** after 6 weeks (*p* < 0.0001),↓from **25.7 (8.9) to 6.4 (8.1)** after 12 weeks (*p* < 0.0001) *CRP normalization*: **70% (21/30)** after 6 weeks *CRP mean* (*SD*): ↓from **2.9 (2.7) to 0.9 (1.0) mg/L** after 6 weeks (*p* < 0.0001), ↓from **2.3 (2.3) to 0.8 (0.6) mg/L** after 12 weeks (*p* = 0.002) *ESR mean* (*SD*): ↓from **29.3 (16.6) to 17.0 (10.9) mm/h** after 6 weeks (*p* < 0.0001), ↓from **25.7 (12.7) to 17.0 (8.2) mm/h** after 12 weeks (*p* = 0.001) *Hemoglobin mean* (*SD*): from **12.2 (1.3) to 12.3 (1.2) g/dL** after 6 weeks (NS), from **12.0 (1.4) to 12.6 (1.3) g/dL** after 12 weeks (NS) *Albumin mean* (*SD*): from **4.2 (2.0) to 4.1 (0.4) g/dL** after 6 weeks (NS), ↑from **3.8 (0.4) to 4.1 (0.4) g/dL** after 12 weeks (*p* < 0.0001)
**Sigall Boneh et al.^32^** Retrospective	50% PEN + CDED for first 6 weeks, 25% PEN + CDED for another 6 weeks (*n* = 17) CDED for 12 weeks (*n* = 4)	✘	12 weeks	*Response*: ↓HBI ≥ 3/remission *Remission*: PGA (based on complete cessation of symptoms eg, diarrhea, abdominal pain, weight loss, rectal bleeding) + HBI < 5	Combined results for PEN + CDED and CDED groups unless stated otherwise. *Response rate*: **90.4% (19/21)** after 6 weeks *Remission rate*: **59% (10/17)** PEN + CDED group, 75% (3/4) CDED group after 6 weeks *HBI mean* (*SD*): ↓from **9.4 (4.2) to 2.6 (3.8)** after 6 weeks (*p* < 0.001), ↓from **8.6 to 2.2** after 12 weeks (*p* < 0.001) CRP mean (SD): ↓from **2.8 (3.4) to 0.7 (0.5) mg/dL** after 6 weeks (*p* = 0.025), ↓from **2.7 to 0.6 mg/dL** after 12 weeks (*p* = 0.021) *Albumin mean* (*SD*): from **3.5 (0.6) to 3.8 (0.5)** after 6 weeks (NS)
**Levine et al.^10^** Parallel RCT	50% PEN + CDED for first 6 weeks, 25% PEN + CDED for another 6 weeks (*n* = 40)	EEN for first 6 weeks, 25% PEN for another 6 weeks (*n* = 38)	12 weeks	*Response*: ↓PCDAI ≥ 12.5/remission *Remission*: PCDAI < 10 + PCDAI ≤ 10/PCDAI < 7.5* CRP normalization: CRP ≤ 5 mg/L *PCDAI without height component	*Response rates*: **85% (34/40)** PEN + CDED group, 85.3% (29/34) EEN group after 6 weeks (PEN + CDED vs EEN: NS) *Remission rates* (*PCDAI ≤ 10*): **80% (32/40)** PEN + CDED group, 73.5% (25/34) EEN group after 6 weeks (PEN + CDED vs EEN: NS); **87.5% (28/32)** PEN + CDED group, 56% (14/25) EEN group after 12 weeks (PEN + CDED vs EEN: *p* = 0.01) *Remission rates* (*PCDAI*≤*10*): **75% (30/40)** PEN + CDED group, 58.8% (20/34) EEN group after 6 weeks (PEN + CDED vs EEN: NS); **75.6% (28/37)** PEN + CDED group, 45.1% (14/31) EEN group after 12 weeks (PEN + CDED vs EEN: *p* = 0.01) *PCDAI median* (*IQR*): ↓from **25 (20-35) to 2.5 (0-7.5)** PEN + CDED group (*p* < 0.001), ↓from 27.5 (18.75-32.5) to 5 (0-10) EEN group (*p* < 0.001) after 6 weeks *CRP normalization*: **51.3% (20/39)** PEN + CDED group, 55.8% (19/34) EEN group after 6 weeks (PEN + CDED vs EEN: NS); **75.9% (22/29)** PEN + CDED group, 47.6% (10/21) EEN group after 12 weeks (PEN + CDED vs EEN: *p* = 0.04) *CRP median* (*IQR*): ↓from **23.6 (9.8-54.2) to 5 (2.7-8.0) mg/L** PEN + CDED group (*p* < 0.001), ↓from 24 (10.1-52.9) to 4.1 (1.3-8.4) mg/L EEN group (*p* < 0.001) after 6 weeks *FC median*: ↓from **3126 to 1744 μg/g** PEN + CDED group (*p* = 0.002), from 2647 to 1021 μg/g EEN group (*p* = 0.011) after 6 weeks (PEN + CDED vs EEN: NS); from **1774 to 732 μg/g** PEN + CDED group (week 6 vs week 12: NS; baseline vs week 12: *p* = 0.001), from 1021 to 1589 μg/g EEN group (week 6 vs week 12: NS; baseline vs week 12: *p* = 0.026) after 12 weeks
**Sigall Boneh et al.^36^** Secondary outcome analysis to a parallel RCT (Levine et al., 2019)	50% PEN + CDED for first 6 weeks, 25% PEN + CDED for another 6 weeks (*n* = 39)	EEN for first 6 weeks, 25% PEN for another 6 weeks (*n* = 34)	12 weeks	*Response*: ↓PCDAI ≥ 12.5/remission *Remission*: PCDAI < 10 *CRP normalization*: CRP < 5 mg/L	*Response rates*: **82% (32/39)** PEN + CDED group, 85% (29/34) EEN group after 3 weeks (PEN + CDED vs EEN: NS) *Remission rates*: **61.5% (24/39)** PEN + CDED group, 64.7% (22/34) EEN group after 3 weeks (PEN + CDED vs EEN: NS) *PCDAI median* (*IQR*): ↓from **25 (20-35) to 5 (0-12.5)** PEN + CDED group (*p* < 0.001), ↓from 27.5 (17.5-32.5) to 5 (0-10) EEN group (*p* < 0.001) after 3 weeks *CRP normalization*: **43.6% (17/39)** PEN + CDED group, 50% (17/34) EEN group after 3 weeks (PEN + CDED vs EEN: NS) *CRP median* (*IQR*): ↓from **28.1 (8-56) to 5 (3.3-9.5) mg/L** PEN + CDED group (*p* < 0.001), ↓ from 24 (9-52) to 5 (1.1-8.7) mg/L EEN group (*p* < 0.001) after 3 weeks
**Urlep et al.^37^** Open-label controlled trial	75% PEN + AID-CD (*n* = 12)	EEN (*n* = 13)	6 weeks	*Clinical response*: ↓PCDAI ≥ 15 *Clinical remission*: PCDAI < 10 *Endoscopic response*: ↓SES-CD ≥ 50% *Endoscopic remission*: SES-CD ≤ 2 *Mucosal healing*: SES-CD = 0	*Clinical response rates*: **100% (11/11)** PEN + AID-CD group, 90.9% (10/11) EEN group (PEN + AID-CD vs EEN: NS) *Clinical remission rates*: **81.8% (9/11)** PEN + AID-CD group, 81.8% (9/11) EEN group (PEN + AID-CD vs EEN: NS) *PCDAI mean* (*SE*): ↓from **31.4 (3.2) to 4.1 (1.4)** PEN + AID-CD group (*p* < 0.001), ↓from 30.5 (3.6) to 6.1 (2.8) EEN group (*p* < 0.001) (PEN + AID-CD vs EEN: NS) *ESR mean* (*SE*): ↓from **38.5 (7.6) to 13.8 (1.4) mm/h** PEN + AID-CD group (*p* = 0.003), ↓from 37.1 (6.5) to 16.3 (3.6) mm/h EEN group (*p* = 0.009) (PEN + AID-CD vs EEN: NS) *CRP mean* (*SE*): ↓from **18.4 (5.8) to 7.9 (0) mg/L** PEN + AID-CD group (*p* = 0.007), ↓from 16.5 (3.1) to 8.8 (1.3) mg/L EEN group (*p* = 0.008) (PEN + AID-CD vs EEN: NS) *Albumin mean* (*SE*): ↑from **39.8 (1.2) to 43.3 (1.3) g/L** PEN + AID-CD group (*p* = 0.012), from 40.0 (1.6) to 43.2 (1.6) g/L EEN group (*p* = 0.049) (PEN + AID-CD vs EEN: NS) *Hemoglobin mean* (*SE*): from **121.8 (3.1) to 124.7 (2.3) g/L** PEN + AID-CD group (NS), from 118.7 (3.5) to 115.0 (4.9) g/L EEN group (NS) (PEN + AID-CD vs EEN: NS) *Platelet count mean* (*SE*): ↓from **435.7 (42.3) to 370.6 (32.7) × 10**^**9**^**/L** PEN + AID-CD group (*p* = 0.003), ↓from 438.4 (34.0) to 344.9 (30.6)** × **10^9^/L EEN group (*p* = 0.017) (PEN + AID-CD vs EEN: NS) *FC mean* (*SE*): ↓from **426.5 (31.8) to 138.2 (25.0) μg/g** PEN + AID-CD group (*p* < 0.001), ↓from 381.1 (37.3) to 206.9 (41.3) μg/g EEN group (*p* = 0.009) (PEN + AID-CD vs EEN: NS) *Endoscopic response rates*: **90.9% (10/11)** PEN + AID-CD group, 63.6% (7/11) EEN group (PEN + AID-CD vs EEN: NS) *Endoscopic remission rates*: **45.5% (5/11)** PEN + AID-CD group, 45.5% (5/11) EEN group (PEN + AID-CD vs EEN: NS) *Mucosal healing rates*: **27.3% (3/11)** PEN + AID-CD group, 45.5% (5/11) EEN group (PEN + AID-CD vs EEN: NS) *SES-CD mean* (*SE*): ↓from **13.5 (1.7) to 3.2 (1.0)** PEN + AID-CD group (*p* < 0.001), ↓from 10.7 (1.8) to 4.6 (1.6) EEN group (*p* = 0.003) (PEN + AID-CD vs EEN: NS)
**Niseteo et al.^38^** Retrospective	EEN for first 1-2 weeks, 50% PEN + CDED for another 6 weeks (*n* = 16) 50% PEN + CDED for 6 weeks (*n* = 4)	EEN for 6-8 weeks (*n* = 41)	6-8 weeks	*Remission*: wPCDAI < 12.5	Combined results for EEN + PEN + CDED and PEN + CDED groups unless stated otherwise. *Remission rates*: **75% (12/16)** EEN + PEN + CDED group, **75% (3/4)** PEN + CDED group, 65.9% (27/41) EEN group *wPCDAI median* (*range*): ↓by **27.5 (−57.5 to −5.0)** PEN + CDED group, ↓by 32.5 (−90.0 to 32.5) EEN group (PEN + CDED vs EEN: NS) *CRP median* (*range*): ↓by **16.3 (−116.0 to 43.8) mg/L** PEN + CDED group, ↓by 11.5 (−166.2 to 84.4) mg/L EEN group (PEN + CDED vs EEN: NS) *Hemoglobin median* (*range*): ↑by **4.0 (−23.0 to 28.0) g/L** PEN + CDED group, ↑by 2.0 (−90.0 to 28.0) g/L EEN group (PEN + CDED vs EEN: NS) *Platelet count median* (*range*): ↓by **57.0 (−334.0 to 292.0) × 10**^**9**^**/L** PEN + CDED group, ↓by 66.0 (−289.0 to 125.0) × 10^9^/L EEN group (PEN + CDED vs EEN: NS) *Leukocyte count median* (*range*): ↓by **4.1 (−7.5 to 1.8) × 10**^**9**^**/L** PEN + CDED group, ↓by 2.7 (−13.0 to 8.6) × 10^9^/L EEN group (PEN + CDED vs EEN: NS)
**Szczubełek et al.^39^** Open-label uncontrolled trial	50% PEN + CDED for first 6 weeks, 25% PEN + CDED for another 6 weeks (*n* = 32)	✘	12 weeks	*Response*: ↓CDAI ≥ 100 *Remission*: CDAI < 150	*Response rates*: **83.3%** after 6 weeks, **85.7%** after 12 weeks *Remission rates*: **76.7%** after 6 weeks, **82.1%** after 12 weeks *CDAI median* (*IQR*): ↓from **253.0 (175.5-373.0) to 53.4 (31.4-143.0)** after 6 weeks (*p* < 0.001), **to 45.0 (24.8-122.0)** after 12 weeks (vs baseline: *p* < 0.001; vs 6 weeks: NS) *CRP median* (*IQR*): from **4.3 (1.1-10.1) to 1.3 (0.5-3.7)** after 6 weeks (NS), **to 1.4 (0.6-4.8) mg/L** after 12 weeks (vs baseline: NS; vs 6 weeks: NS) *ESR median* (*IQR*): from **9.0 (2.8-22.3) to 10.0 (6.0-16.5)** after 6 weeks (NS), **to 6.0 (4.0-16.5) mm/h** after 12 weeks (vs baseline: NS; vs 6 weeks: NS) *Albumin mean* (*SD*): from **4.4 (0.5) to 4.4 (0.5)** after 6 weeks (NS), **to 4.3 (0.6) g/dL** after 12 weeks (vs baseline: NS; vs 6 weeks: NS) *White blood cell count mean* (*SD*): from **6.8 (2.3) to 7.3 (2.8)** after 6 weeks (NS), **to 6.8 (3.0) 10**^**3**^**/µL** after 12 weeks (vs baseline: NS; vs 6 weeks: NS) *Platelet count mean* (*SD*): from **303.1 (91.3) to 301.6 (89.2)** after 6 weeks (NS), **to 281.3 (75.7) 10**^**3**^**/µL** after 12 weeks (vs baseline: NS; vs 6 weeks: NS) *Hemoglobin mean* (*SD*): from **13.3 (1.8) to 13.6 (1.5)** after 6 weeks (NS), **to 13.5 (1.4) g/dL** after 12 weeks (vs baseline: NS; vs 6 weeks: NS) *Hematocrit mean* (*SD*): **from 40.1 (4.3) to 41.1 (3.6)** after 6 weeks (NS), **to 41.1 (3.3) %** after 12 weeks (vs baseline: NS; vs 6 weeks: NS) *FC median* (*IQR*): ↓from **393.0 (58.9-969.0) to 231.0 (37.2-577.5)** after 6 weeks (NS), **to 122.0 (34.0-387.8) μg/g** after 12 weeks (vs baseline: *p* = 0.021; vs 6 weeks: NS)
**Matuszczyk et al.^18^** Open-label uncontrolled trial	50% PEN + CDED for first 6 weeks, 25% PEN + CDED for another 6 weeks (*n* = 48)	✘	12 weeks	*Response*: ↓PCDAI ≥ 12.5 *Remission*: PCDAI < 10 *FC response*: ↓FC ≥ 50% *FC normalization*: < 250 µg/g	*Clinical response*: **68.9%** (20/29) in patients with baseline PCDAI ≥ 10 *Clinical remission*: **55.2%** (16/29) in patients with baseline PCDAI ≥ 10 *PCDAI median* (*IQR*): ↓from **12.5 (17.5) to 5.0 (7.5)***p* = 0.0002) *CRP median* (*IQR*): ↓from **1.0 (1.8) to 0.2 (0.3) mg/dL** (*p* = 0.0002) *ESR median* (*IQR*): ↓from **21.0 (19.5) to 11.0 (11.0) mm/h** (*p* = 0.0014) *FC response*: **56.3%** (27/48) *FC normalization*: **35.4%** (17/48) *FC median* (*IQR*): ↓from **1045 to 363 µg/g** (*p* = 0.0002)
**Yanai et al.^40^** Parallel RCT	PEN (1000 kcal/day) + CDED + vitamin D (2000 IU/day) supplementation for first 6 weeks, PEN (600 kcal/day) + CDED + calcium and vitamin D (2000 IU/day) supplementation for next 6 weeks, CDED + calcium and vitamin D (2000 IU/day) supplementation for last 12 weeks (*n* = 19)	CDED + calcium and vitamin D (2000 IU/day) supplementation for first 6 weeks, CDED + calcium and vitamin D (2000 IU/day) supplementation for next 6 weeks, CDED + calcium and vitamin D (2000 IU/day) supplementation for last 12 weeks (*n* = 21)	24 weeks	*Clinical response*: ↓HBI ≥ 3 *Clinical remission*: HBI < 5 *Endoscopic remission*: SES-CD ≤ 3	*Clinical response rates*: **73.7% (14/19)** PEN + CDED group, 66.7% (14/21) CDED group after 6 weeks (PEN + CDED vs CDED: NS) *Clinical remission rates*: **68.4% (13/19)** PEN + CDED group, 57.1% (12/21) CDED group after 6 weeks (PEN + CDED vs CDED: NS); **63.2% (12/19)** PEN + CDED group, 47.6% (10/21) CDED group after 12 weeks (PEN + CDED vs CDED: NS); **63.2% (12/19)** PEN + CDED group, 38.2% (8/21) CDED group after 24 weeks (PEN + CDED vs CDED: NS) *HBI median*: ↓from **8.0 to 3.0** after 6 weeks, **to 3.0** after 12 weeks, **to 2.0** after 24 weeks, PEN + CDED group; ↓from 6.0 to 4.0 after 6 weeks, to 3.0 after 12 weeks, to 3.5 after 24 weeks, CDED group (PEN + CDED vs CDED: NS) *CRP median* (*IQR*): ↓from **15.8 (6.9-36.1) to 8.8 (5.3-17.7)** after 6 weeks, **to 7.4 (5.7-19.5)** after 12 weeks, **to 8.0 (5.3-18.9) mg/L** after 24 weeks, PEN + CDED group; ↓from 12.1 (8.7-43.7) to 8.2 (5.5-43.7) after 6 weeks, to 6.1 (4.3-33.6) after 12 weeks, to 7.7 (5.0-35.0) mg/L after 24 weeks, CDED group, in patients with elevated baseline CRP (PEN + CDED vs CDED: NS) *FC median* (*IQR*): ↓from **229.0 (97.6-1050.0) to 268.0 (127.0-859.0)** after 6 weeks, **to 104.1 (65.4-370.3) μg/g** after 12 weeks, PEN + CDED group; ↓from 294.5 (53.5-1620.0) to 216.0 (50.3-761.0) after 6 weeks, to 97.3 (49.2-212.0) μg/g after 12 weeks, CDED group (PEN + CDED vs CDED: NS) *Endoscopic remission rates*: **53% (8/15)** PEN + CDED group, 46% (6/13) CDED group after 24 weeks (PEN + CDED vs CDED: NS) *SES-CD median* (*IQR*): ↓by **5.0 (−6.2 to 1.0)** points after 24 weeks (*p* = 0.0025) in all 22 patients with paired colonoscopies (no paired comparisons within each study group presented by the authors)
**Arcucci et al.^19^** Parallel RCT	50% PEN + CDED for first 6 weeks, 25% PEN + CDED for another 6 weeks (*n* = 11)	Unrestricted diet (*n* = 10)	12 weeks	*Remission*: PCDAI < 10	*Remission rates*: **100% (4/4)** PEN + CDED group, 33% (1/3) control group, in patients with baseline PCDAI ≥ 10 *CRP median* (*IQR*): from **1.0 (0.4-1.7) to 0.4 (0.2-1.7) mg/L** PEN + CDED group (NS), from 1.5 (0.2-9.1) to 0.6 (0.2-0.75) mg/L control group (NS) *Albumin median* (*IQR*): from **4.3 (4.1-4.4) to 4.2 (4.0-4.4) g/dL** PEN + CDED group (NS), from 4.1 (3.9-4.3) to 4.4 (4.2-4.4) g/dL control group (NS) *ESR median* (*IQR*): from **9.0 (6.5-35.0) to 10.0 (8.5-16.0) mm/h** PEN + CDED group (NS), from 15.0 (6.5-28.8) to 16.5 (13.5-30.0) control group (NS) *Hematocrit median* (*IQR*): from **39.0 (36.5-42.0) to 38.0 (35.2-41.8) %** PEN + CDED group (NS), from 36.5 (35.0-41.5) to 38.8 (36.4-42.7) % control group (NS) *FC median (IQR)*: ↓from **750.0 (594.5-914.0) to 339.0 (104.0-413.5) μg/g** PEN + CDED group (*p* = 0.0049), from 719.5 (503.0-967.3) to 671.0 (470.3-862.5) μg/g control group (NS)
**Fliss-Isakov et al.^21^** Retrospective	50% PEN + CDED for first 6 weeks, 25% PEN + CDED for another 6 weeks (*n* = 22) CDED for 12 weeks (*n* = 50)	✘	12 weeks	*Response*: ↓HBI ≥ 3 *Remission*: HBI < 5	*Response rate*: **45.8%** (33/72) *Remission rates*: **62.5%** (30/48) after 6 weeks, **62.5%** (30/48) after 12 weeks *HBI mean* (*SD*): ↓fy **3.7 (3.5)** (*p* < 0.001) *CRP mean* (*SE*): ↓from 2.0 (1.5) to 0.75 (1.0) mg/dL* (*p* < 0.001) *FC mean* (*SD*): ↓by **668 (1284) μg/g** (*p* < 0.001) *Values estimated from Figure 2C
**Jijón Andrade et al.^43^** Retrospective	50% PEN + CDED for first 6 weeks, 25% PEN + CDED for another 6 weeks, CDED for last 12 weeks (*n* = 15: treatment-naïve (*n* = 9), with LOR to biologics (*n* = 6)	✘	24 weeks	*Remission*: PCDAI < 10	*Remission rates*: **100% (15/15)** after 6 weeks, **100% (15/15)** after 12 weeks, **77% (10/13)** after 24 weeks PCDAI median: ↓by **13.8** after 6 weeks (*p* = 0.09), ↓by **15** after 12 weeks (*p* = 0.002), ↓by **15** after 24 weeks (*p* = 0.002), in treatment-naïve patients; significant ↓ in PCDAI after 12 weeks (*p* = 0.049) in patients with LOR to biologics (values not presented) *Albumin median*: ↑by **4 g/L** after 6 weeks (*p* = 0.03), ↑by **5 g/L** after 12 weeks (*p* = 0.03), ↑by **3 g/L** after 24 weeks (*p* = 0.016) in treatment-naïve patients; no changes in patients with LOR to biologics (values not presented) *ESR median*: ↓by **7 mm/h** after 12 weeks (*p* = 0.021), ↓by **5 mm/h** after 24 weeks (*p* = 0.027) in treatment-naïve patients; no changes in patients with LOR to biologics (values not presented) *Hemoglobin median*: ↑by **0.9 g/dL** after 24 weeks (*p* = 0.048) in treatment-naïve patients; no changes in patients with LOR to biologics (values not presented) *CRP*: no changes (values not presented) *FC median*: ↓by **585 μg/g** after 6 weeks (*p* = 0.02), ↓by **801 μg/g** after 12 weeks (*p* = 0.016), ↓by **723 μg/g** (*p* = 0.019) **μg/g** after 24 weeks, in treatment-naïve patients; no changes in patients with LOR to biologics (values not presented)

^a^Duration is presented for the duration of intervention unless stated otherwise.

Results are presented at the end of the intervention/follow-up duration unless stated otherwise. Values in bold represent data for patients receiving partial enteral nutrition.

Abbreviations: AID-CD, anti-inflammatory diet for Crohn’s disease; CDAI, Crohn’s Disease Activity Index; CDED, Crohn’s disease exclusion diet; CRP, C-reactive protein; EEN, exclusive enteral nutrition; ESR, erythrocyte sedimentation rate; FC, fecal calprotectin; HBI, Harvey-Bradshaw Index; IQR, interquartile range; IU, international units; LOR, loss of response; NS, not significant; PCDAI, Paediatric Crohn’s Disease Activity Index; PEN, partial enteral nutrition; PGA, Physician Global Assessment; RCT, randomized control trial; SD, standard deviation; SE, standard error; SES-CD, simplified endoscopic score for Crohn’s disease; wPCDAI, weighted Paediatric Crohn’s Disease Activity Index.

Clinical remission rates following 6 weeks of treatment ranged from 59% to 100%. The highest remission rate of 100% was observed in a retrospective study without a control group, which used PEN with CDED in 15 treatment-naïve children who also received azathioprine concurrently.^43^ Two studies reported remission rates after 12 weeks of treatment based on a subset of patients with active disease (FC used to define active CD), with one showing a 55% remission rate^18^ and the other reporting 100%, albeit based on only 4 participants.^19^ Interestingly, 2 other small-sized studies, including 1 RCT, found no difference in efficacy rates between patients receiving PEN with CDED and those receiving CDED alone.^21,40^

The majority of these 12 articles (10 of 12, 83%) reported some improvements in systemic inflammatory biomarkers. Two articles^19,39^ that did not report any improvements, either included patients that had normal baseline biomarker levels^39^ or did not have active symptoms.^19^ Four articles, including 2 reports from the same RCT, found no difference between PEN alongside exclusion diets and compared with EEN,^10,36–38^ albeit the studies were not powered to show non-inferiority. This was also the case in another RCT, where no differences were found between CDED with PEN and CDED alone. However, PEN was used at lower doses compared to previous research by the same authors.^40^

All 7 articles measuring FC levels reported significant decreases after treatment with PEN alongside exclusion diets.^10,18,19,21,37,39,40,43^ Interestingly, 2 articles, including 1 RCT, comparing PEN with exclusion diets against EEN found no differences in the median baseline FC change^10^ or levels^37^ after 6 weeks between the 2 groups. Nonetheless, in an RCT comparing PEN with CDED to EEN, median FC levels remained high in both groups, indicating ongoing intestinal inflammation,^10^ and were higher than those reported by other independent studies using EEN.^44^ In the only study where CDED + PEN was compared to CDED alone, as mentioned above, similar reductions and levels of FC at intervention completion were observed between the 2 groups, although almost half of the patients had normal FC levels at study enrollment.^40^

Two studies assessing endoscopic outcomes reported similar outcomes with PEN alongside exclusion diets compared to EEN or exclusion diet alone. In 1 study, a 46% endoscopic remission rate was observed in patients with moderate baseline endoscopic activity after 6 weeks of 75% PEN with an exclusion diet resembling CDED, which was not different from treatment with EEN alone (46%).^37^ There were also no differences in endoscopic remission rates observed between the 2 groups treated with PEN alongside CDED and CDED alone after 24 weeks, in patients with mild baseline endoscopic activity: 53% after PEN with CDED and 46% after CDED alone (*p* = 0.705).^40^ The authors did not present paired comparisons (before vs after treatment) in endoscopic scores within each study group. In all 22 patients with paired colonoscopies at baseline and week 24, median SES-CD decreased by 5·0 points (IQR −6·2 to −1·0); *p* = 0.0025. It is noteworthy though that the criteria used to define endoscopic remission differed between the 2 studies (Table 1).

In contrast to using PEN alongside unrestricted diet, there is suggestive evidence to indicate that combining high-dosage PEN with specific exclusion diets can be effective in treating active CD, with comparable efficacy to EEN.

#### 3.1.3 PEN combined with biological treatment

Three studies (3 of 27, 11%) combined PEN with anti-TNFα agents (infliximab [IFX]) in patients with active CD; all in adults^9,24,25^ (Table 2). One study was an RCT,^9^ and the other 2 were of retrospective design.^24,25^ Two reported clinical remission rates,^9,24^ but none assessed changes in inflammatory biomarkers or in endoscopic findings.

**Table 2:** Summary of study results investigating the clinical efficacy of partial enteral nutrition combined with biological treatments as induction treatment in patients with active Crohn’s disease.

Study reference	Intervention description	Comparison description	Duration^a^	Response/remission criteria	Results
**Matsumoto et al.^24^** Retrospective	PEN at > 1500 kcal/day + IFX (5 mg/kg) (*n* = 49)	TPN at > 1500 kcal/day + IFX (5 mg/kg) (*n* = 36) Unrestricted diet + IFX (5 mg/kg) (*n* = 12) (control group)	2 weeks	*Response*: ↓CDAI ≥ 70 *Remission*: CDAI < 150	*Response rates*: **46.9% (23/49)** PEN group, 44.4% (16/36) TPN group, 58.3% (7/12) control group (between-group: NS) *Remission rates*: **30.6% (15/49)** PEN group, 47.2% (17/36) TPN group, 33.3% (4/12) control group (between-group: NS)
**Tanaka et al.^25^** Retrospective	PEN at ≥ 900 kcal/day + IFX (5 mg/kg at weeks 0, 2, and 6) (*n* = 51) PEN at < 900 kcal/day)/no PEN + IFX (5 mg/kg at weeks 0, 2, and 6) (*n* = 59)	✘	16 weeks	Response: ↓HBI*>3 (inflammatory CD)/50% of the number of previously described draining fistulas (fistulizing CD) *Only 1 point allocated for ↓ in number of liquid stools	*Response rates*: **68.4%** ≥ 900 kcal/day PEN group, **32.4%** < 900 kcal/day PEN group in patients with inflammatory CD (≥ 900 kcal/day vs < 900 kcal/day: *p* = 0.0026), values not presented for patients with fistulizing CD (≥ 900 kcal/day vs < 900 kcal/day: NS)
**Hisamatsu et al.^9^** RCT	PEN at 900-1200 kcal/day + IFX dose escalation (10 mg/kg every 8 weeks) (*n* = 14)	IFX dose escalation (10 mg/kg every 8 weeks) (*n* = 6)	16 weeks	*Response*: ↓CDAI ≥ 50 *Remission*: CDAI < 150	*Response rates*: **63.6% (7/11)** PEN + IFX group, 20% (1/5) IFX group after 8 weeks (PEN + IFX vs IFX: NS); **72.7% (8/11)** PEN + IFX group, 0% (0/5) IFX group after 16 weeks (PEN + IFX vs IFX: *p* = 0.0256) *Remission rates*: **54.5% (6/11)** PEN + IFX group, 20% (1/5) IFX group after 8 weeks (PEN + IFX vs IFX: NS); **72.7% (8/11)** PEN + IFX group, 0% (0/5) IFX group after 16 weeks (PEN + IFX vs IFX: *p* = 0.0256)

^a^Duration is presented for the duration of intervention unless stated otherwise.

Results are presented at the end of intervention/follow-up duration unless stated otherwise. Values in bold represent data for patients receiving partial enteral nutrition.

Abbreviations: CDAI, Crohn’s Disease Activity Index; HBI, Harvey-Bradshaw Index; IFX, infliximab; NS, not significant; PEN, partial enteral nutrition; RCT, randomized control trial; TPN, total parenteral nutrition.

Clinical remission rates with PEN and IFX combination therapy ranged from 31% to 73%. The lowest rate was observed with a 2-week regimen of IFX and PEN at > 1500 kcal/day, with no differences in efficacy when compared to IFX monotherapy or a combination therapy of IFX with total parenteral nutrition (TPN) also at > 1500 kcal/day.^24^ The highest remission rate was reported in an RCT, after 16 weeks of PEN at 900-1200 kcal/day combined with IFX dose escalation, showing significant improvements compared to IFX dose escalation monotherapy.^9^ Nonetheless, this study had a small sample size and was terminated prematurely due to ethical concerns of not offering PEN to the monotherapy group. One study, while not reporting remission rates, observed a higher clinical response rate of 68% after 16 weeks of IFX combined with PEN at ≥ 900 kcal/day, compared to a 32% response rate with IFX with lower PEN dosages, or no PEN, particularly in patients with inflammatory disease behavior (*p* = 0.007), but not in those with fistulizing disease.^25^

There is currently low-quality evidence to suggest that concomitant PEN may improve response and remission rates to IFX therapy and IFX dose escalation therapy in patients with inflammatory, but most likely not in patients with fistulizing CD.

#### 3.1.4. Meta-analysis of PEN as induction treatment

A meta-analysis examining the efficacy of PEN in active CD was performed using data from 27 articles described in the first theme of this review (Tables 1 and 2, and Supplementary Table 3). The analysis focused on clinical remission rates, the most frequently reported outcome within this theme. Among these, 8 studies included a comparison group of patients on an unrestricted diet alongside standard of care treatments,^9,19,20,24,28,29,34,35^ with 6 of them reporting clinical remission rates,^9,19,20,24,28,34^ and subsequently included in the meta-analysis. Of these, 2 studies used PEN with IFX,^9,24^ 1 study alongside CDED,^19^ while the remaining 3 used PEN alongside unrestricted diet and standard of care treatments. A study that reported outcomes separately for 2 different types of PEN formula^20^ was treated as 2 independent studies.

Six other articles compared PEN against EEN in this theme.^8,10,30,33,37,38^ Clinical remission rates were reported in 5 of them,^8,10,30,37,38^ and these studies were included in a second meta-analysis comparing PEN against EEN. Of these, 3 used PEN with exclusion diets,^10,37,38^ and 2 with an unrestricted diet.^8,30^

**Figure 2:**
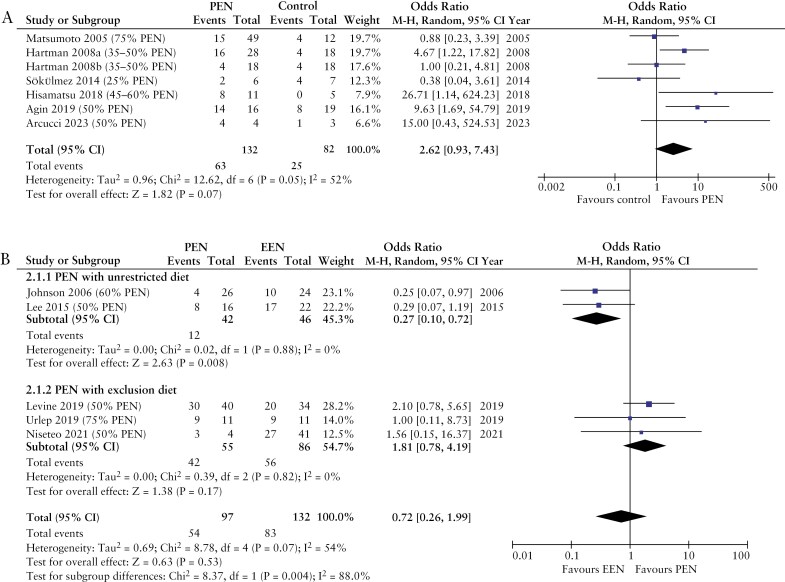
Forest plots of clinical remission rates in patients with active Crohn’s disease after treatment with partial enteral nutrition in comparison to: (A) an unrestricted diet, (B) exclusive enteral nutrition. PEN, partial enteral nutrition; EEN, exclusive enteral nutrition.

The meta-analysis comparing PEN against an unrestricted diet in patients with active CD revealed a trend toward higher remission rates in patients receiving PEN, although this did not reach statistical significance (*p* = 0.07), and significant heterogeneity was also observed, likely attributed to variable study designs, variations in PEN dosage and duration, and differences in background treatments (*p* = 0.05) (Figure 2A). Likewise, the meta-analysis comparing PEN against EEN showed that PEN alongside an unrestricted diet was less effective than EEN alone for 6-8 weeks but was equally effective as EEN when used alongside exclusion diets (OR [95% CI]: 1.81 [0.78-4.19], *p* = 0.17) (Figure 2B).

The findings of the meta-analysis showed that PEN, alongside an unrestricted diet, was less effective as induction therapy than treatment with EEN for 6-8 weeks (Figure 2). However, when PEN was used alongside exclusion diets, it was equally effective as EEN.

### 3.2. PEN as maintenance treatment

Thirty articles (30 of 64, 47%) studied the use of PEN as maintenance treatment.^15–17,22,45–69^ Articles were categorized based on the treatments used to achieve remission including non-biological treatments, biological treatments, and surgical gut resection.

#### 3.2.1. PEN in the maintenance of remission in CD achieved with non-biological treatments

Nineteen articles (19 of 30, 63%) explored the role of PEN as maintenance therapy in patients who achieved remission with non-biological drug treatments including EEN and PEN^15,16,22,45–48,50–52,56,57,61,63,64,66,67,69,70^ (Supplementary Table 4). Among these, 17 used PEN alongside an unrestricted diet, 1 with a low-fat diet (20-30 g/day),^52^ and another one with a low-residue and low-fat diet (< 20 g/day).^63^ Eleven studies included children,^15,16,22,45,46,61,63,64,66,67,70^ 7 adults,^47,48,50–52,56,57^ and 1 all age patients.^69^ Four studies were RCTs,^47,51,56,57^ while the majority (10 of 19, 53%) were of retrospective design.^15,16,46,50,61,63,64,66,69,70^ Among these 19 studies, 17 reported clinical relapse rates,^15,16,22,46–48,50–52,56,57,61,63,64,66,67,69^ 5 reported changes in systemic inflammatory biomarkers,^47,48,52,69,70^ 2 in FC,^67,69^ and 1 described endoscopic findings and changes in mucosal inflammatory biomarkers.^52^

Within the available body of literature, clinical relapse rates varied across studies, ranging from 0% to 89%. The lowest relapse rate was observed in a retrospective study where 4 patients received daily PEN dosages of 500-1000 mL without additional drug treatments over a mean duration of 15 months.^16^ In contrast, the highest relapse rate, reaching 89%, was reported in a prospective observational study where patients received low-dosage PEN at 20%-25% of energy requirements for 1 year, showing no difference compared to those on an unrestricted diet.^67^ An RCT also using low-dosage PEN reported a 31% relapse rate after 6 months of PEN at 500 kcal/day, which was equal to treatment with mesalamine (800 mg 3 times/day), a drug that has now been demonstrated to be ineffective in the treatment of CD.^56^ Three RCTs using higher dosages of PEN, ranging from 35% to 50% of energy requirements (35%-50%,^47^ 50% [900-1200 kcal/day],^51^ ≥ 900 kcal/day^57^), reported relapse rates of up to 58%. In one of these studies, no difference in relapse rates was found between patients receiving PEN with either elemental or polymeric formula,^47^ and in another study, PEN use was associated with lower relapse rates compared to unrestricted diet.^51^ In the last study, PEN showed superior effectiveness to no treatment (*p* = 0.035) and comparable effectiveness to 6-Mercaptopurine at doses of 0.5-1.5 mg/kg/day.^57^

Articles in this theme focused on patients in remission primarily defined by disease activity indices, although in 4 out of 5 studies, several patients had abnormal disease biomarkers.^47,48,69,70^ Of these, only 1 reported improvements in CRP levels over time with PEN, provided at 35%-50% of energy requirements, with no such changes seen in a group of patients following an unrestricted diet.^48^ Similarly, another study found no differences in albumin, white blood cell count, hemoglobin, CRP, and ESR between PEN and thiopurines or methotrexate at 6- and 12-month follow-up.^69^ This is in contrast to findings from a retrospective study, which observed lower albumin levels among patients receiving 3 months of 50% PEN compared to those on an unrestricted diet (*p* = 0.007).^70^ However, children who received PEN had lower weight, body mass index (BMI), and albumin levels at EEN initiation (prior to PEN use).^70^ In an open-label controlled trial where patients had normal baseline values of albumin, 1 year of PEN at 1200-1800 kcal/day administered overnight via a nasogastric tube with a low-fat diet prevented a drop in albumin (*p* = 0.04), deterioration of endoscopic scores (*p* = 0.04), and rise in mucosal inflammatory biomarkers (IL-1β, IL-6, and TNFα) compared to a group of patients on an unrestricted diet.^52^

Two studies in this subtheme measured FC levels. In a prospective observational study, children who adhered to the prescribed PEN formula had lower FC levels 17 days after completing EEN compared to patients who did not adhere to PEN (*p* = 0.049).^67^ No significant differences in FC levels were seen in another study between patients receiving PEN and those receiving thiopurines or methotrexate after 6 and 12 months of treatment.^69^

On the basis of the literature above, it can be concluded that there is some evidence to suggest that PEN at > 35% of energy requirements may prolong remission achieved with conventional, non-biological treatments including steroids, EEN, PEN, and TPN. However, most studies relied solely on subjective disease activity indices, whereas assessment of disease activity using inflammatory biomarkers and endoscopic findings was limited. It is also important to acknowledge that pharmacological treatment paradigms have shifted away from the traditional use of the less effective immunomodulator monotherapy to maintain disease remission to biologics and other advanced agents that offer better efficacy.^71^ Against these new potent agents, there is currently no literature to compare the effectiveness of PEN as maintenance therapy.

#### 3.2.2. PEN in the maintenance of remission in CD achieved with biological treatments

Six articles (6 of 30, 20%), all involving adults, investigated the adjunctive role of PEN in maintaining disease remission achieved with anti-TNFα agents^17,54,58–60,62^ (Table 3). The majority of these studies (4 of 6, 67%) used IFX as the anti-TNFα agent^54,58,60,62^; 1 study used adalimumab,^17^ and another study used either agent.^59^ Most of the studies (4 of 6, 67%) were retrospective,^17,58,60,62^ with none reporting results from RCTs. All 6 articles assessed LOR rates,^17,54,58–60,62^ 2 reported changes in systemic inflammatory biomarkers,^17,59^ and 1 study reported on changes in serum TNFα levels.^17^ None of the articles provided data on intestinal inflammatory biomarkers or endoscopic findings.

**Table 3:** Summary of study results investigating the clinical efficacy of partial enteral nutrition as maintenance treatment in patients with Crohn’s disease in remission achieved with biological treatments.

Study reference	Intervention description	Comparison description	Duration^a^	LOR criteria	Results
**Yamamoto et al.** ^54^ Open-label controlled trial	PEN at 1200-1500 kcal/day overnight via NGT + low-fat diet (20-30 g/day) + IFX (5 mg/kg every 8 weeks) (*n* = 32)	Unrestricted diet + IFX (5 mg/kg every 8 weeks) (*n* = 24)	56 weeks	CDAI ≥ 150	*LOR*: **78% (25/32)** PEN group, 67% (16/24) control group (PEN vs control: NS)
**Sazuka et al.^58^** Retrospective	PEN at ≥ 600 kcal/day + IFX (5 mg/kg every 8 weeks) (*n* = 29) PEN at < 600 kcal/day/no PEN + IFX (5 mg/kg every 8 weeks) (*n* = 45)	✘	Median (range) follow-up: 85 (25-402) weeks	CDAI ≥ 150 + ↑CDAI > 70 + elevated CRP	*LOR*: **20.6% (6/29) ≥ **600 kcal/day PEN group, **52.3% (23/45)** < 600 kcal/day PEN group (≥ 600 kcal/day vs < 600 kcal/day: *p* = 0.0043)
**Hirai et al.^60^** Retrospective	PEN at ≥ 900 kcal/day + IFX (5 mg/kg every 8 weeks) (*n* = 45) PEN at < 900 kcal/day/no PEN + IFX (5 mg/kg every 8 weeks) (*n* = 57)	✘	~544.1 days follow-up	CRP ≥ 1.5 mg/dL/hospitalization/need for additional treatment/IFX dose interval escalation < 4 weeks	*LOR*: **31.1% (14/45)** ≥ 900 kcal/day PEN group, **57.8% (33/57) < **900 kcal/day PEN group (≥ 900 kcal/day vs < 900 kcal/day: *p* = 0.009)
**Kamata et al.^62^** Retrospective	PEN at ≥ 900 kcal/day + IFX (5 mg/kg every 8 weeks) (*n* = 28) PEN at < 900 kcal/day/no PEN + IFX (5 mg/kg every 8 weeks) (*n* = 97)	✘	Mean (SD) follow-up: 799 (398) days ≥ 900 kcal/day PEN group; 771 (497) days < 900 kcal/day PEN group	Clinical symptoms recurrence + IFX dose escalation/IFX dose interval escalation	*LOR*: **3.6% (1/28)** ≥ 900 kcal/day PEN group, **20.6% (20/97)** < 900 kcal/day PEN group (≥ 900 kcal/day vs < 900 kcal/day: *p* < 0.042)
**Sugita et al.^17^** Retrospective	PEN at ≥ 900 kcal/day + ADA (40 mg every 2 weeks) (*n* = 25) PEN at < 900 kcal/day)/no PEN + ADA (40 mg every 2 weeks) (*n* = 92)	✘	Mean (SD) follow-up: 1327 (480) days	HBI ≥ 5/hospitalization/surgery/need for additional concomitant therapy/need for dose escalation of concomitant therapy	*LOR*: lower in ≥ 900 kcal/day PEN group vs < 900 kcal/day PEN group (*p* = 0.023) (no values presented) *CRP*: no differences between ≥ 900 kcal/day PEN group vs < 900 kcal/day PEN group after 28 and 52 weeks (no values presented) *Serum TNFα*: lower in ≥ 900 kcal/day PEN group vs < 900 kcal/day PEN group after 28 (*p* = 0.044) and 52 (*p* = 0.043) weeks (no values presented)
**Hirai et al.^59^** Open-label controlled trial	PEN at > 900kcal/day + IFX/ADA (*n* = 37)	Unrestricted diet + IFX/ADA (*n* = 35)	2 years	CDAI ≥ 200/need for additional treatment/anti-TNFα dose escalation/anti-TNFα dose interval escalation/surgery/need for PEN use in control group/hospitalization	*LOR*: **35% (13/37)** PEN group, 37% (13/35) control group (PEN vs control: NS) *CDAI mean* (*SD*): ↓from **273.3 (50.1) to 100.1 (94.0)** PEN group, ↓from 268.3 (45.9) to 124.1 (94.0) control group (PEN vs control: NS) *CRP mean* (*SD*): **0.9 (1.8) mg/dL** PEN group, 1.6 (2.7) mg/dL control group after 2 years (PEN vs control: NS) *Albumin mean* (*SD*): **3.9 (0.7) g/dL** PEN group, 3.8 (0.6) g/dL control group after 2 years (PEN vs control: NS)

^a^Duration is presented for the duration of intervention unless stated otherwise.

Results are presented at the end of intervention/follow-up duration unless stated otherwise. Values in bold represent data for patients receiving partial enteral nutrition.

Abbreviations: ADA, adalimumab; CDAI, Crohn’s Disease Activity Index; CRP, C-reactive protein; HBI, Harvey-Bradshaw Index; IFX, infliximab; LOR, loss of response; NGT, nasogastric tube; NS, not significant; PEN, partial enteral nutrition; SD, standard deviation; TNFα, tumor necrosis factor alpha.

Within the available literature, LOR rates with combination treatment of PEN and anti-TNFα agents ranged from 4% to 58%. Two open-label controlled trials found no benefit of concomitant PEN in reducing chances of LOR in comparison to anti-TNFα monotherapy. In the first trial, PEN at 1200-1500 kcal/day administered via a nasogastric tube for 56 weeks alongside a low-fat diet (20-30 g/day) did not reduce the risk of LOR.^54^ Similarly, the second trial, where patients received 2 years of PEN at > 900 kcal/day alongside an unrestricted diet, also found no benefit of PEN.^59^ In contrast, the results of the remaining 4 articles showed a significant benefit of concomitant PEN for patients prescribed higher dosages (≥ 600 kcal/day,^58^ ≥ 900 kcal/day^17,60,62^) compared to those receiving lower dosages or no PEN.

These findings were not confirmed when using objective biomarkers, as both studies measuring systemic inflammatory biomarkers did not observe differences between groups on PEN at ≥ 900 kcal/day compared to lower dosages or no PEN.^17,59^ One of these 2 studies also found that serum TNFα levels were significantly lower in patients prescribed higher PEN dosages.^17^ None of the studies assessed anti-TNFα drug levels or anti-drug antibodies, which are frequent causes of LOR.

The available literature of the benefit of PEN in the maintenance of remission in CD achieved with biological treatment is of low quality. However, there is a suggestion in some studies that PEN may reduce the risk of LOR to anti-TNF agents.

#### 3.2.3. PEN for maintenance of CD in remission after surgical gut resection

Five articles (5 of 30, 17%), all including adult patients, investigated the efficacy of postoperative PEN in maintaining remission after gut resection in patients with CD, the majority of which (3 of 5) were of retrospective design^49,53,55,65,68^ (Supplementary Table 5). Clinical relapse rates were reported in 4 studies,^49,53,55,65^ endoscopic relapse rates in 3,^53,55,65^ and surgical relapse rates in 2,^55,68^ with none measuring systemic or intestinal inflammatory biomarkers.

In one small (*n* = 40) open-label controlled trial, 1 year of PEN at 1200-1800 kcal/day administered overnight via nasogastric tube alongside a low-fat (20-30 g/day) diet reduced clinical and endoscopic relapse rates when compared to an unrestricted diet. In this previous study, no patients were given biologics post-operatively.^53^ Nonetheless, this effect was not sustained at the 5-year follow-up, with no differences observed in clinical, endoscopic, and surgical relapse rates between patients on PEN and those on an unrestricted diet.^54,55^ Three other articles retrospectively categorized patients into 2 groups based on their PEN intakes and found that patients receiving higher PEN dosages (> 1200 kcal/day,^49^ or ≥ 900 kcal/day^65,68^) had lower clinical relapse rates,^49^ and longer clinical,^65^ endoscopic,^65^ and surgical^68^ remission intervals than patients prescribed lower dosages or no PEN. The mean patient follow-up times in these studies were approximately 2.6,^49^ 4.3,^65^ and 4.7 years.^68^

Taken together, the findings from the studies discussed above show that there is some low-quality evidence to suggest that postoperative PEN may reduce the rates of clinical, endoscopic, and surgical recurrence in patients with CD.

#### 3.2.4. Meta-analysis of PEN for maintenance of CD in remission

A meta-analysis investigating the efficacy of PEN as a maintenance treatment in CD was conducted using data from 30 articles (Table 3, and Supplementary Tables 4 and 5), and focusing on clinical relapse rates, the most commonly reported outcome measure. Among these, 19 articles compared PEN to an unrestricted diet with or without other background therapies^15,16,22,45,46,48,51–57,59,61,66,67,69,70^ with 17 reporting on clinical relapse rates.^15,16,22,46,48,51–57,59,61,66,67,69^ We analyzed clinical relapse rates up to 1-year follow-up, aligning with the reporting timeframe in most studies. A follow-up study from the same cohort of patients reporting data after 5 years was excluded.^55^ From these 16 studies, PEN was combined with a low-fat diet in 2 studies^52–54^ and with anti-TNFα agents in a third one.^59^

**Figure 3:**
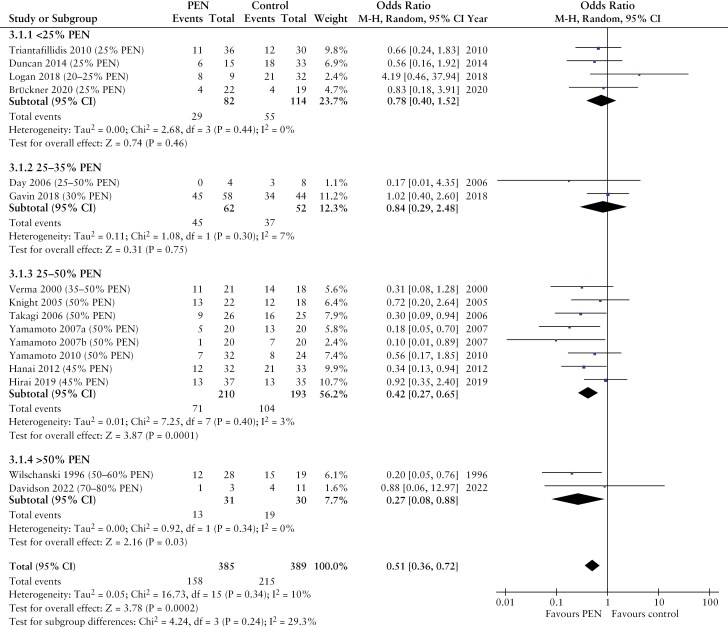
Forest plot of clinical relapse rates in patients with Crohn’s disease in remission after treatment with partial enteral nutrition in comparison to an unrestricted diet. PEN, partial enteral nutrition.

The meta-analysis revealed a dose-dependent effect of PEN in preventing disease relapse, with benefits detectable at dosages of at least 35%. Conversely, PEN at dosages of less than 35% did not have similar benefits, with none of the 6 studies reporting statistically significant effects (Figure 3). Collectively, there is low-quality evidence to suggest that the use of PEN at intake > 35% of energy requirements can prevent disease relapses (35%-50% PEN: OR [95% CI]: 0.42 [0.27-0.65], *p* = 0.0001; > 50% PEN: OR [95% CI]: 0.27 [0.08-0.88], *p* = 0.03).

### 3.3. PEN and nutritional outcomes

Twenty-nine articles (29 of 64, 45%) investigated, mainly as secondary outcomes, the effect of PEN on nutritional outcomes in patients with CD.^8,10,20,22,23,26,29–31,33–35,37–40,42,43,45–48,52,56,66,70,72–74^ Assessed nutritional outcomes included anthropometric measures, body composition, malnutrition risk, handgrip strength, blood biomarkers of nutritional status, and pubertal staging. The literature is presented separately for adult and pediatric patients.

#### 3.3.1. PEN on nutritional outcomes of children

Fifteen of the articles (15 of 29, 52%) included children^8,10,20,22,26,29,30,34,37,38,43,45,46,66,70^ (Supplementary Table 6), with the majority (8 of 15, 53%) having a retrospective study design,^20,26,34,38,43,46,66,70^ whereas 2 presented findings from RCTs.^8,10^ All 15 articles reported changes in anthropometric measures (eg weight, height, and BMI), 2 in body composition,^8,22^ 3 in blood biomarkers of nutritional status,^29,34,43^ 1 in Tanner staging,^45^ and another one assessed handgrip strength.^22^

Out of the 10 articles examining changes in body weight, 7, including 2 RCTs, reported significant weight gain with PEN,^8,10,26,29,30,38,45^ while 3 found no significant effects.^20,37,43^ Among the 6 articles assessing BMI changes, 3 reported increases,^20,38,66^ with 1 observing changes only in patients receiving Modulen IBD formula but not with Nutren formula, potentially indicating sample selection bias or lower tolerance with the latter supplement.^20^ Selection bias may have influenced the outcomes of at least 5 articles, as children assigned to receive PEN had lower baseline weight *z*-scores compared to those in comparator groups.^22,30,34,66,70^

Among the 6 articles assessing changes in height, 4 reported increases, either in all participants^26,29,45^ or specifically in those with Tanner Stage 1-3 pubertal development.^22^ Increased height velocity was also observed in a retrospective study after 1 year of 50%-60% PEN compared to an unrestricted diet.^46^ In another open-label controlled trial, more children receiving PEN reached a height velocity of ≥ 4 cm/year compared to children following an unrestricted diet, although statistical significance was not reported.^45^

In terms of body composition changes, 1 RCT observed an increase in skinfold thickness but not in mid-upper arm circumference with 6 weeks of 50% PEN, with no differences between PEN and EEN.^8^ In contrast, no significant changes in body composition or bone geometry, assessed with peripheral quantitative computed tomography, were observed after 1 year of 25% PEN when compared to an unrestricted diet.^22^

Two out of 3 articles assessing changes in blood biomarkers of nutritional status during PEN reported improvements.^29,43^ Four weeks of PEN providing 20 kcal/kg/day improved several nutritional biomarkers in blood at the 1-year follow-up, including micronutrient levels, although the reason for measurements at such a late stage is unclear.^29^ One study found that 24-week CDED regime coupled with PEN increased serum iron levels,^43^ contrasting with another study reporting no improvements to total blood protein levels with 8 weeks of 50% PEN.^34^

A study that assessed Tanner staging found that 25% (2 of 8) of children progressed from Stage 1 to Stage 2 after 1 year of PEN at 1000-1500 kcal/day compared to no children (0 of 6) on an unrestricted diet.^45^ No improvement in handgrip strength was observed after 1 year of 25% PEN in another study.^22^

There is evidence to suggest that PEN may improve anthropometry in children with CD. However, there is currently insufficient evidence to confirm the benefits of PEN on body composition, blood biomarkers of nutritional status, pubertal development, and handgrip strength in this patient group.

#### 3.3.2. PEN on nutritional outcomes of adults

Fourteen articles (14 of 29, 48%) explored the effect of PEN on nutritional outcomes in adult patients^23,31,33,35,39,40,42,47,48,52,56,72–74^ (Supplementary Table 7) with 5 of them presenting findings from RCTs.^40,47,56,72,73^ Among these, 12 reported changes in body weight or BMI,^23,31,33,35,39,40,42,47,48,52,56,72^ 4 in body composition,^23,35,56,72^ 8 in blood biomarkers of nutritional status,^23,31,33,39,56,72–74^ 2 in malnutrition risk assessed with the Subjective Global Assessment,^35,42^ and 1 reported changes in handgrip strength.^35^

The majority of articles (7 of 12, 58%) that examined changes to body weight or BMI, including 2 RCTs, reported increases in these parameters after treatment with PEN.^23,31,35,42,52,56,72^ Selection bias may have occurred in some studies such as where patients who were assigned to receive 50% PEN had lower body weight and were at higher risk of undernutrition at study enrollment compared to patients assigned to follow an unrestricted diet.^35^

All 4 articles that assessed body composition reported an increase in at least one body composition compartment,^23,35,56,72^ including mid-arm circumference,^35,56,72^ mid-arm muscle circumference,^35,72^ skinfold thickness,^35,56,72^ abdominal circumference,^35^ fat-free mass,^23^ and fat mass,^23^ although in this last study, no effect on body fat percentage was observed.^23^

Among the 8 articles that measured nutritional blood biomarkers, 5 reported improvements in at least one of them following treatment with PEN.^23,31,39,56,72^ These included increases in serum prealbumin,^72^ folic acid,^39,56^ ferritin,^56^ high-density lipoprotein,^56^ vitamin D3,^23,39^ vitamin B12,^39^ calcium,^39^ total protein,^31^ histidine,^31^ tryptophan,^31^ as well as a decrease in low-density lipoprotein.^56^ In 1 study, improvement in serum vitamin B12 achieved with 6-week 50% PEN with CDED was lost after another 6 weeks of 25% PEN with CDED.^39^ While most studies observed similar findings, 2 reported no improvements in nutritional blood biomarkers with PEN,^33,73^ and in a single cross-sectional study, PEN use was associated with lower serum selenium concentrations.^74^

The conflicting findings were reported by 2 studies that assessed the risk of malnutrition; both using the Subjective Global Assessment nutrition tool. In an open-label uncontrolled trial including malnourished adults, a 12-week supplementary PEN at 200-600 kcal/day alongside standard of care induction treatments reduced the risk of malnutrition.^42^ However, in a second open-label controlled trial, no improvement was observed in patients receiving 50% PEN, although the majority of patients were not malnourished at study enrollment.^35^ Additionally, no effect of PEN on handgrip strength was observed in the same study.^35^

Overall, there is good quality evidence to suggest that PEN improves anthropometry, body composition, and blood biomarkers of nutritional status in adults with CD. Nonetheless, its effect on the risk of malnutrition and handgrip strength in this patient population requires further research.

#### 3.3.3. Meta-analysis of PEN on nutritional outcomes

A meta-analysis investigating the effect of PEN on nutritional outcomes in patients with CD was conducted using data extracted from 29 articles (15 including children and 14 adults) described in the third theme of this review (Supplementary Tables 6 and 7).^8,10,20,22,23,26,29–31,33–35,37–40,42,43,45–48,52,56,66,70,72–74^ The meta-analysis, conducted separately for pediatric and adult patients, focused on anthropometric parameters, the most commonly reported nutritional outcome.

##### 3.3.3.1. Meta-analysis of PEN on nutritional outcomes of children

Out of the 15 articles involving children, 8 compared PEN against unrestricted diet alongside standard of care treatments.^20,22,29,34,45,46,66,70^ Three of these studies were excluded due to either reporting outcomes after 1 year, while PEN was used for only for 4 weeks,^29^ or reporting data with ranges, precluding calculations of SD.^45,66^ The remaining 5 articles were included in the meta-analysis.^20,22,34,46,70^ Hartman et al., reported results separately for 2 groups of patients receiving PEN with different formulas, and these were considered as 2 independent studies.^20^ Three out of the 5 articles reported changes in weight SD scores (*z*-scores) or weight,^20,34,70^ 3 in BMI *z*-scores or BMI,^20,22,70^ and all 5 in height *z*-scores, height or height velocity.^20,22,34,46,70^

Five other articles compared PEN against EEN,^8,10,30,37,38^ but 1 study was excluded as data were reported only with value ranges.^38^ All 4 studies included in the meta-analysis, reported changes in weight *z*-scores or weight,^8,10,30,37^ 2 in BMI *z*-scores or BMI,^30,37^ while none reported changes in height *z*-scores or height.

**Figure 4:**
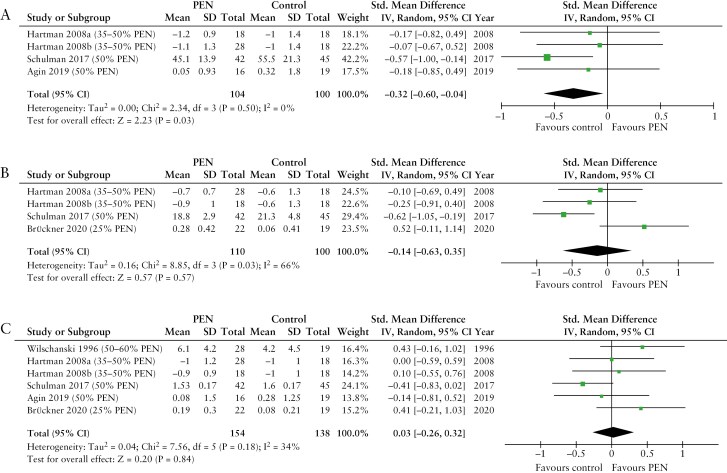
Forest plots of: (A) weight *z*-scores or weight, (B) BMI *z*-scores or BMI, (C) height *z*-scores or height, in children with Crohn’s disease after treatment with partial enteral nutrition in comparison to an unrestricted diet. PEN, partial enteral nutrition.

**Figure 5:**
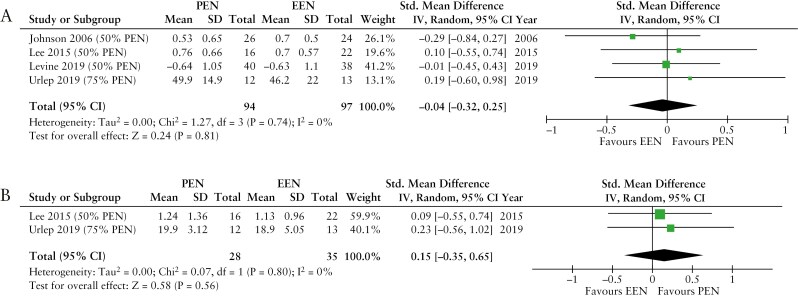
Forest plots of: (A) weight *z*-scores or weight, (B) BMI *z*-scores or BMI, in children with Crohn’s disease after treatment with partial enteral nutrition in comparison to exclusive enteral nutrition. PEN, partial enteral nutrition; EEN, exclusive enteral nutrition.

The meta-analysis comparing PEN against an unrestricted diet in children with CD showed lower weight *z*-scores or weight in the PEN group (Figure 4A); albeit this effect was primarily driven by a large size retrospective study where children who received PEN had lower weight and BMI at study enrollment compared to those on an unrestricted diet.^70^ No significant differences were found in BMI *z*-scores or BMI (Figure 4B) and height *z*-scores or height (Figure 4C) between the 2 groups. It is also important to note significant heterogeneity in studies when BMI was the outcome measure assessed (Figure 4B). No differences were found between PEN and EEN in weight *z*-scores or weight (Figure 5A) and BMI *z*-scores or BMI (Figure 5B).

##### 3.3.3.2. Meta-analysis of PEN on nutritional outcomes of adults

Out of the 14 articles involving adults, 7 compared PEN against an unrestricted diet^35,48,52,56,72–74^ of which 5 assessed weight changes.^35,48,52,56,72^ Two articles were excluded as neither post-treatment values nor changes from baseline were reported.^48,52^ The remaining 3 articles included in the meta-analysis comprised of a parallel design RCT^56^ and a crossover RCT,^72^ as well as an open-label controlled trial.^35^ Harries et al. used PEN as a supplement in malnourished patients aiming to reach a total intake of 3000 kcal/day, and PEN dosage was not reported.^72^ Another study reported results separately for 2 groups of patients receiving PEN: One using the Nutren formula (Ferreira 2020a) and the other using the Modulen IBD formula (Ferreira 2020b). These 2 groups were treated as 2 independent studies.^35^

**Figure 6:**
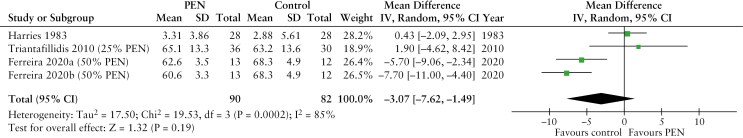
Forest plot of weight changes in adults with Crohn’s disease after treatment with partial enteral nutrition in comparison to an unrestricted diet.

The meta-analysis, with a significant level of heterogeneity (*p* = 0.0002), revealed no benefit of PEN, compared to unrestricted diet, in improving weight in adults with CD (Figure 6). A high risk of selection bias in the available literature was identified. For instance, in the study by Ferreira et al., patients allocated to PEN had a lower weight than those allocated to the unrestricted diet group, potentially indicating that PEN may have been used to correct nutritional deficits in patients at risk of malnutrition.^35^

### 3.4. PEN and quality of life

Five articles (5 of 64, 8%) examined the effects of PEN on quality of life scores of patients with CD, with 4 involving adults and using the Inflammatory Bowel Disease Questionnaire (IBDQ),^23,39,75,76^ and a single study in children with CD using the IMPACT-III questionnaire^30^ (Supplementary Table 8). Among these, 2 were open-label uncontrolled trials,^23,39^ and 1 was an RCT,^76^ 3 included patients with active CD,^23,30,39^ 1 with CD in remission,^76^ and 1 with a combination of both.^75^

Collectively, the majority (4 of 5, 80%) of studies reported improvements in patients’ quality of life with PEN, whether prescribed alongside CDED^39^ or an unrestricted diet.^23,30,75^ A prospective observational study reported that 50% PEN was as effective as EEN and anti-TNFα agents in improving the overall quality of life of children with active CD,^30^ while an RCT using PEN at 900-1,200 kcal/day did not show improvements compared to an unrestricted diet, although patients had high IBDQ scores, indicating good quality of life at study enrollment.^76^ Based on the available evidence, it appears that treatment with PEN may maintain quality of life in patients with CD who are in remission, as well as improving this in those with active disease.

### 3.5. The effect of PEN on additional outcomes

Four articles (4 of 64, 6%) investigated the effect of PEN on other outcomes, which could not form their own distinct themes,^73,76–78^ including hospitalization rates, healthcare costs, and surgical rates. Among these, 2 studies in adults assessed hospitalization rates.^73,78^ In the first of these studies, PEN at 500-750 kcal/day did not reduce hospitalization rates when compared to an unrestricted diet, although it decreased hospitalization days.^73^ However, compliance with PEN was low, with a significant proportion of patients discontinuing their treatment and showing poor adherence to the prescribed PEN dosage. In contrast, a retrospective study found that patients prescribed PEN at > 900 kcal/day had a 36% lower likelihood of hospitalization compared to those receiving lower dosages of PEN or no PEN (hospitalization rates: 50% [76/153] > 900 kcal/day PEN; 61% [70/115] < 900 kcal/day PEN).^78^

PEN providing 900-1200 kcal/day did not influence healthcare costs when compared to an unrestricted diet in an RCT of adults with CD.^76^ In contrast, the use of PEN reduced the likelihood of gastrointestinal surgery in 2 studies.^73,77^ An RCT reported a 13% decrease in surgery rates with PEN provided at 500-750 kcal/day compared to an unrestricted diet,^73^ and a retrospective study reported a 45% decrease in surgery rates with PEN at > 900 kcal/day compared to lower dosages of PEN or no PEN (Hazard Ratio [95%] of 0.55 [0.32-0.94], *p* = 0.029).^77^

### 3.6. Associations between residual dietary intake and disease outcomes during PEN

In this final theme, we explored the relationship between residual dietary intake during PEN and disease outcomes. In only 1 study addressing this aspect, patients achieving > 50% reduction in baseline FC levels consumed fewer servings of red meat (mean [SD] servings/day: 0.4 [0.4] vs 1.7 [1.1]; unadjusted *p* = 0.01; adjusted for symptoms *p* = 0.03).^30^ This association was not observed when clinical remission rates defined by PCDAI were used to assess changes in disease activity. There is currently scarce data to suggest that a certain dietary pattern or nutrient intake is associated with better disease outcomes in patients with CD who receive treatment with PEN.

## 4. Discussion

In this systematic review and meta-analysis of 64 articles, we provided a comprehensive overview of the current literature exploring the role of PEN in patients with CD. While most research focused on PEN as a maintenance treatment, our analysis also suggests efficacy in treating active disease and improving nutritional status parameters and quality of life. We also identified limited literature addressing its effects on hospitalization rates, healthcare costs, and the likelihood of future risk of gastrointestinal surgery. Notably, the evidence predominantly comprises low-quality retrospective and observational studies, with only 11 distinct RCTs.

Current evidence suggests the effectiveness of 50% PEN as induction therapy, particularly when combined with exclusion diets like CDED, showing comparable efficacy to EEN. However, further research is warranted to evaluate the efficacy of PEN as an induction therapy at various dosages and also without exclusion diets, especially considering indications that high-dosage PEN (~80%) alongside an unrestricted diet can effectively manage active CD, as demonstrated in a previous study.^26^ Although encouraging, these findings require confirmation through well-designed RCTs. The single available RCT, investigating 50% PEN with an unrestricted diet in active CD, had several limitations, including high dropout rates, warranting caution when interpreting its findings.^8^ The question also remains unanswered as to whether the observed improvements in studies using PEN with exclusion diets were due to PEN itself, its combination with the daily mandatory foods covering the majority of patients’ energy requirements (>90%), or the dietary elimination. A single RCT suggested that CDED alone may be as effective as CDED with PEN.^40^ However, the study’s methodology differed from previous studies by the same group, with lower PEN doses, most patients having normal baseline FC levels and only mild endoscopic activity, and, importantly, the lack of statistical power for non-inferiority comparisons.

When studying PEN as a maintenance treatment through a formal meta-analysis, a dose–response relationship was indicated. PEN, provided at dosages equivalent to or exceeding 35% of energy requirements, effectively prevented relapse; a finding which was reported elsewhere.^79,80^ It is noteworthy, though, that these observations are predominantly derived from retrospective or observational study designs, with most studies using clinical disease activity indices rather than objective biomarkers to assess disease activity. In the same theme, preliminary evidence supports the use of postoperative PEN to prevent clinical, endoscopic, and surgical relapse, with effects observed after 1 year of treatment, though likely not sustained at 5 years.

A critical aspect of managing CD in the era of biologics and advanced therapies is improving primary response rates and preventing LOR. Preliminary evidence in patients with active CD indicated that PEN exceeding 900 kcal/day may improve response rates to anti-TNFα agents either as initial treatment^25^ or as dose escalation when used for 16 weeks.^9^ Interestingly, these benefits were not observed after 2 weeks of PEN,^24^ and in patients with fistulizing disease.^25^ Several studies also suggest that PEN at dosages exceeding 600-900 kcal/day may be effective in preventing LOR to anti-TNFα agents in patients in remission.^17,58,60,62^ While the conventional approach involves combining anti-TNFα agents with immunomodulators to prevent LOR, a strategy with associated increased risk of side effects,^81^ PEN may be a potentially safer alternative. The evidence supporting PEN as a combination therapy with anti-TNFα agents is promising but currently limited and of low quality, lacking well-controlled and adequately powered studies, all originating from a single country, thus limiting the generalizability of the findings.

The benefits of PEN extend beyond disease management, with some evidence supporting its positive effect on various nutritional parameters and the quality of life of both pediatric and adult patients. In the pediatric population, PEN and EEN demonstrated comparable effects on anthropometry and body composition, but evidence on pubertal development, blood biomarkers of nutritional status, and functional parameters is limited. In adult patients, current evidence suggests that PEN improves nutritional outcomes, including anthropometric measures, body composition, blood biomarkers of nutritional status, and reducing malnutrition risk. However, research on its effects on functional parameters is also limited in this patient population. Additionally, a significant amount of data supports the positive effect of PEN on quality of life. Although there is limited and poor-quality evidence, there are also indications of broader potential benefits of PEN, including lower hospital admission rates, reduced length of hospitalization, and a lower risk of gastrointestinal surgery.

This study identified critical gaps and outlined key considerations for future investigations into the role of PEN in CD. The majority of low-quality evidence highlights the need for well-designed RCTs reporting results with objective disease biomarkers and mechanistic investigations. Clinical disease activity indices, often subjective and prone to reporting bias and placebo effects, present challenges, particularly in patients with mild-to-moderate disease activity.^82^ Recurring limitations of the current research include the use of arbitrary cutoffs to categorize patients’ PEN intake and selection bias, leading to differences between groups at study enrollment, either as a result of offering PEN to patients with more severe disease or malnutrition, or allowing patients to choose their group assignment. Treatment compliance, mostly assessed with self-reported measures, requires careful consideration for future studies. A more accurate assessment of compliance assessment would involve objective biomarkers, as exemplified by a single study measuring plasma phospholipids in patients receiving formula enriched with fish oil.^23^ Furthermore, to ensure the effectiveness of PEN in managing CD, its consumption as a replacement rather than a supplement to the habitual diet may be imperative. Studies, including the RCT by Johnson et al., observed overconsumption of the habitual diet during PEN by 26% of EAR,^8^ potentially contributing to the lowest remission rate of only 15% reported across all studies. Similarly, the observed low rates of patients achieving FC levels indicative of endoscopic remission after 8 weeks of 50% PEN reported by another observational study may also be explained by overconsumption of the habitual diet, with a median energy intake of 151% of EAR.^30^ Limited data on dietary intakes prevented us from conducting a subset meta-analysis, highlighting the need for further investigation and confirmation through meta-analysis as more data become available. Although it is plausible that the efficacy of both EEN and PEN is due to limited exposure to dietary triggers, this aspect has only been studied in a single study, which found lower red meat consumption in children with better improvements to FC.^30^ Future research in this area is needed to gain insights into the mechanisms of action of PEN in managing CD and to propose strategies to improve its effectiveness. It is important to note that the present study did not include literature on the effect of PEN on postoperative outcomes and complication rates, such as anastomotic leaks and stoma formation.

In conclusion, the current evidence suggests that high-dosage PEN can effectively prevent relapse in patients with CD in remission, improve disease outcomes in those with active disease, especially when used alongside exclusion diets, and enhance nutritional status and quality of life. While limited and low-quality data also suggest potential benefits of PEN to prevent postoperative relapse, and in combination with anti-TNFα agents, further high-quality research is warranted before recommendations can be made.

## Supplementary Material

jjae177_suppl_Supplementary_Materials

## Data Availability

No new data were created, and data sharing is not applicable to this article.

## Publication statement

We confirm that this manuscript including related data, figures, and tables has not been previously published and is not under consideration elsewhere.
