# Supplementary material for: Partial Enteral Nutrition in the Management of Crohn’s Disease: A Systematic Review and Meta-Analysis
Source: J Crohns Colitis. 2024 Nov 20;19(5):jjae177. doi: 10.1093/ecco-jcc/jjae177 (PMC12087569; doi:10.1093/ecco-jcc/jjae177)
Supplement: jjae177_suppl_Supplementary_Materials [file jjae177_suppl_supplementary_materials.docx]

**Supplementary Table 1:** Reasons for exclusion of 10 studies initially disputed by reviewers.

| **Study reference** | **Reason for exclusion** |
| --- | --- |
| Kirschner, B. S., Klich, J. R., Kalman, S. S., Defavaro, M. V. & Rosenberg, I. H. 1981. Reversal of growth retardation in Crohn's disease with therapy emphasizing oral nutritional restitution. Gastroenterology, 80, 10-5. | Ineligible study design - case report. |
| Yamazaki, Y., Fukushima, T., Sugita, A., Takemura, H. & Tsuchiya, S. 1990. The medical, nutritional and surgical treatment of fistulae in Crohn's disease. Jpn J Surg, 20, 376-83. | This study did not assess any of the predetermined outcomes. |
| Hirakawa, H., Fukuda, Y., Tanida, N., Hosomi, M. & Shimoyama, T. 1993. Home elemental enteral hyperalimentation (HEEH) for the maintenance of remission in patients with Crohn's disease. Gastroenterol Jpn, 28, 379-84. | It was not clear if this study used PEN or EEN. |
| Lionetti, P., Callegari, M. L., Ferrari, S., Cavicchi, M. C., Pozzi, E., De Martino, M. & Morelli, L. 2005. Enteral nutrition and microflora in pediatric Crohn's disease. JPEN J Parenter Enteral Nutr, 29, S173-5; discussion S175-8, S184-8. | This study used PEN after EEN, but no data was provided for PEN. |
| Takagi, S., Shiga, H., Umemura, K., Endo, K., Kakuta, Y., Takahashi, S., Kinouchi, Y. & Shimosegawa, T. 2010. Scheduled maintenance therapy with infliximab improves the prognosis of crohn's disease: A single center prospective cohort study in Japan. Tohoku Journal of Experimental Medicine, 220, 207-215. | This study used PEN used IFX, but no data was provided for PEN. |
| Wu, G. J., Gong, L., Xu, M. & Zhu, Y. W. 2016. Infliximab combined with enteral nutrition for management of moderate to severe Crohn's disease. World Chinese Journal of Digestology, 21, 2221-2227. | This study used PEN used IFX, but no data was provided for PEN. |
| Lev-Tzion, R., Ben-Moshe, T., Abitbol, G., Ledder, O., Levine, A., Peleg, S., Millman, P., Shaoul, R., Shamaly, H., On, A. & Et Al. 2019. The effect of nutritional therapy on bone mineral density and bone metabolism in paediatric Crohn's disease. Journal of Crohn's & colitis, 13, S465‐. | This is a secondary outcome analysis to another study already included in the review, but this study combined patients on PEN+CDED and EEN together. |
| Ryan, J. J., Hanes, D. A., Bradley, R. D. & Contractor, N. 2019. Effect of a Nutrition Support Formula in Adults With Inflammatory Bowel Disease: A Pilot Study. Global Advances In Health and Medicine, 8. | This study reported combined results for CD and UC. |
| Wall, C. L., Mccombie, A. M., Gearry, R. B. & Day, A. S. 2019. Newly Diagnosed Crohn's Disease Treated with Standard of Care or Enteral Nutrition: Psychological Outcomes over 6 Months. Inflamm Intest Dis, 4, 7-13. | This is a secondary outcome analysis to another study already included in the review, but this study combined patients on PEN and EEN together. |
| Marques, J. G., Shokry, E., Frivolt, K., Werkstetter, K. J., Brückner, A., Schwerd, T., Koletzko, S. & Koletzko, B. 2020. Metabolomic Signatures in Pediatric Crohn's Disease Patients with Mild or Quiescent Disease Treated with Partial Enteral Nutrition: A Feasibility Study. SLAS Technol, 2472630320969147. | This study did not assess any of the predetermined outcomes. |

Abbreviations used: CD: Crohn’s Disease; CDED: Crohn’s Disease Exclusion Diet; EEN: Exclusive Enteral Nutrition; PEN: Partial Enteral Nutrition; IFX: Infliximab; UC: Ulcerative Colitis.

**Supplementary Table 2:** Summary of study characteristics.

| **Study reference** | **Study design** | **Country** | **Population*** | **Concomitant treatments** | **Formula type** | **Comparator group** | **Assessment of compliance** | **Clinical disease activity results** | **Blood inflammatory biomarkers results** | **Faecal biomarkers results** | **Endoscopic results** |
| --- | --- | --- | --- | --- | --- | --- | --- | --- | --- | --- | --- |
| Harries et al., 1983^72^ | Crossover RCT | UK | 28 (11 F, 17 M) adults (mean (SD): 35 (15) y PEN group; 39 (16) y control group) with CD and malnutrition | Prednisolone (64%), SASP (43%) | Polymeric (Ensure Plus) | **✓** | 3-day food diary | ✘ | **✓** | ✘ | ✘ |
| Imes et al., 1986^73^ | Parallel RCT | Canada | 125 (67 F, 58 M) adults (mean (SD): 34.2 (10.5) y group 1 (PEN); 34.0 (11.9) y group 2 (PEN + diet counselling); 29.2 (7.9) y group 3 (unrestricted diet); 36.4 (10.3) y group 4 (unrestricted diet + diet counselling)) with CD | Prednisolone (44% group 1, 29% group 2, 35% group 3, 44% group 4), 5-ASA (56% group 1, 36% group 2, 45% group 3, 31% group 4) | Polymeric (Ensure) | **✓** | Calculating the amount of returned formula | **✓** | ✘ | ✘ | ✘ |
| Aiges et al., 1989^45^ | Open-label controlled trial | US | 14 adolescents (13-17 y) with CD in remission with impaired linear growth (<4 cm/year) and Tanner stage I | SASP (50% PEN group, 16% control group), prednisone (<10 mg/day, 38% PEN group), SASP and prednisone (<10 mg/day, 83% control group), no medication (12.5% PEN group) | Polymeric (Osmolite, Isocal) | **✓** | Not specified | **✓** | ✘ | ✘ | ✘ |
| Wilschanski et al., 1996^46^ | Retrospective | Canada | 65 (29 F, 36 M) children (7-17 y) with active CD treated with EEN: 47/65 children in remission (PCDAI≤20) advised to continue with PEN as a maintenance treatment | EEN initiation: no medication (38%); SASP or 5-ASA alone (20%); metronidazole (20%); prednisone (<0.5 mg/kg/day, 18%); prednisone (>0.5 mg/kg/day, 20%). Oral steroids tapered and discontinued, and other medications (except for metronidazole) discontinued at the start of EEN | Elemental (Vivonex, Vital HN) or semi-elemental (Peptamen) | **✓** | ✘ | **✓** | ✘ | ✘ | ✘ |
| Verma et al., 2000^48^ | Open-label controlled trial | UK | 39 (27 F, 12 M) adults (mean (SEM): 39.2 (3.9) y PEN group; 42 (3.3) y control group) with CD in remission (CDAI<150 + absence of GI symptoms) | Prednisolone (64.1%), AZA (15.4%), 5-ASA (41%) | Elemental (E028 Extra) | **✓** | Calculating the amount of returned formula | **✓** | **✓** | ✘ | ✘ |
| Verma et al., 2001^47^ | Parallel RCT | UK | 33 (23 F, 10 M) adults (17-76 y) with steroid-dependent CD in remission (CDAI≤150 + absence of GI symptoms + ESR<20 mm/h) | Steroids (100%), AZA (42.4%), 5-ASA (15.2%) | Elemental (E028) or polymeric (Fortsip) | ✘ | Calculating the amount of returned formula | **✓** | **✓** | ✘ | ✘ |
| Kuroki et al., 2003^74^ | Cross-sectional | Japan | 53 (10 F, 43 M) adults (16-77 y) with active CD (CDAI>150) or CD in remission (CDAI≤150): CDAI range: 13-414 | SASP (23%), prednisolone (6%), no other treatments (75%) | Polymeric (Ensure Liquid) and/or elemental (Elental) | **✓** | ✘ | **✓** | ✘ | ✘ | ✘ |
| Esaki et al., 2005^49^ | Retrospective | Japan | 40 (15 F, 25 M) adults (17-71 y) with CD in remission (CDAI<150) achieved with surgery | 5-ASA (35%), no steroids or thiopurines (6-MP/AZA) post-surgery | Elemental (Elental, Enterued) or polymeric (Ensure Liquid, Racol, Clinimeal) | ✘ | Not specified | **✓** | ✘ | ✘ | ✘ |
| Knight et al., 2005^15^ | Retrospective | UK | 40 children in remission achieved with EEN | No information | Elemental (E028) or polymeric (Ensure Plus) | **✓** | ✘ | **✓** | ✘ | ✘ | ✘ |
| Matsumoto et al., 2005^24^ | Retrospective | Japan | 97 (21 F, 76 M) IFX-naïve adults (17-53 y) with active CD (CDAI≥150) | Oral 5-ASA (99%), oral prednisolone (27%), thiopurines (23%) | Elemental (Elental) or polymeric (Ensure) | **✓** | ✘ | **✓** | ✘ | ✘ | ✘ |
| Day et al., 2006^16^ | Retrospective | Australia | 27 (10 F, 17 M) children (3.8-16 y) with active CD (PCDAI>15) treated with EEN (24/27 children completed EEN, and 12/24 children achieved remission with EEN): 4/12 (0 F, 4 M) children used PEN as a maintenance treatment after EEN | No other treatments in PEN group; 5-ASA and/or AZA in control group | Polymeric (Modulen IBD, Osmolite) | **✓** | ✘ | **✓** | ✘ | ✘ | ✘ |
| Esaki et al., 2006^50^ | Retrospective | Japan | 145 (37 F, 108 M) adults (14-68 y) with CD in remission (CDAI<150 + radiological/endoscopic improvements of active intestinal lesions) achieved with TPN | 5-ASA (37.9%) | Elemental (Elental, Enterued) and/or polymeric (Ensure Liquid, Racol, Clinimeal) | ✘ | Not specified | **✓** | ✘ | ✘ | ✘ |
| Johnson et al., 2006^8^ | Parallel RCT | UK | 50 (21 F, 29 M) children (3.8-16.0 y) with active CD (PCDAI>20) | No information | Elemental (E028 Extra) | **✓** | 3-day food diary | **✓** | **✓** | ✘ | ✘ |
| Takagi et al., 2006^51^ | Parallel RCT | Japan | 51 (14 F, 37 M) adults (mean (SD): 30.8 (11.1) y PEN group; 28.9 (8.1) y control group) with CD in remission (CDAI<150) | AZA (11.8%) | Elemental (Elental) | **✓** | Not specified | **✓** | ✘ | ✘ | ✘ |
| Tanaka et al., 2006^25^ | Retrospective | Japan | 110 (35 F, 75 M) IFX-naïve adults (median (IQR): 32 (28-36) y) with active (75 luminal, 35 fistulising) CD | 5-ASA (97.3%), prednisolone (21.8%), AZA (9.1%), TPN (21.8%) | Elemental (Elental) | ✘ | Not specified | **✓** | ✘ | ✘ | ✘ |
| Yamamoto et al., 2007a^52^ | Open-label controlled trial | Japan | 40 (13 F, 27 M) adults (mean (SE): 29 (3.9) y PEN group; 31 (4.5) y control group) with CD in remission (CDAI<150) | No other treatments | Elemental (Elental) | **✓** | Clinical interview | **✓** | **✓** | ✘ | **✓** |
| Yamamoto et al., 2007b^53^ | Open-label controlled trial | Japan | 40 (14 F, 26 M) adults (mean (SE): 31 (3.7) y PEN group; 33 (3.9) y control group) with CD in remission (CDAI<150) achieved with surgery | No steroids, immunomodulators, nor IFX prior to clinical recurrence | Elemental (Elental) | **✓** | Clinical interview | **✓** | ✘ | ✘ | **✓** |
| Hartman et al., 2008^20^ | Retrospective | Israel | 64 (27 F, 37 M) children and adolescents (5-20 y) with moderate CD (PCDAI 15-30, n=29), severe CD (PCDAI>30, n=30) or with CD in remission (PCDAI<15, n=5) | 5-ASA (78.1%), 6-MP (15.6%), steroids (29.7%), IFX (6.3%), antibiotics (9.4%) | Polymeric (Modulen IBD, Ensure Plus) | **✓** | ✘ | **✓** | **✓** | ✘ | ✘ |
| Kuriyama et al., 2009^75^ | Cross-sectional | Japan | 126 (51 F, 75 M) adults (18-86 y) with CD | Current use of steroids (17%): total prednisolone<10g (88%), total prednisolone≥10g (12%); current use of immunomodulators (11%); current or previous use of IFX (8%) | Elemental (Elental) and/or polymeric (Ensure Liquid, Racol) | **✓** | ✘ | **✓** | ✘ | ✘ | ✘ |
| Takagi et al., 2009^76^ | Secondary outcome analysis to parallel RCT (Takagi et al., 2006) | Japan | 51 (14 F, 37 M) adults (mean (SD): 30.8 (11.1) y PEN group; 28.9 (8.1) y control group) with CD in remission (CDAI<150) | AZA (11.8%) | Elemental (Elental) | **✓** | Not specified | **✓** | ✘ | ✘ | ✘ |
| Takahashi et al., 2009^77^ | Retrospective | Japan | 153 (39 F, 114 M) patients with CD | AZA (10%), IFX (17%) | Elemental (Elental) | ✘ | ✘ | ✘ | ✘ | ✘ | ✘ |
| Triantafillidis et al., 2010^56^ | Parallel RCT | Greece | 66 (25 F, 41 M) adults (17-70 y) with CD in remission (CDAI<150) | No other treatments | Polymeric (Modulen IBD) | **✓** | ✘ | **✓** | ✘ | ✘ | ✘ |
| Watanabe et al., 2010^78^ | Retrospective | Japan | 268 (63 F, 205 M) adults (mean (SD): 31.9 (6.42) y) with CD | AZA/6-MP (25%) | Elemental (Elental) | ✘ | ✘ | ✘ | ✘ | ✘ | ✘ |
| Yamamoto et al., 2010^54^ | Open-label controlled trial | Japan | 56 (20 F, 36 M) adults (mean (SE): 31 (1.6) y PEN group; 33 (1.6) control group) with CD in remission (CDAI<150) achieved with IFX | 5-ASA (95%), AZA (36%) | Elemental (Elental) | **✓** | Clinical interview | **✓** | ✘ | ✘ | ✘ |
| Wiese et al., 2011^23^ | Open-label uncontrolled trial | US | 28 (22 F, 6 M) adults (20-75 y) with clinical signs of active CD | Stable medication dosing except for steroids which could be tapered. Steroids (50%), 5-ASA (20%), immunomodulators (20%) | Polymeric formula (Inflammatory bowel disease nutrition formula, IBDNF) enriched with fish oil, prebiotics, and antioxidants | ✘ | 3-day food diary + measurements of plasma phospholipids (formula enriched with fish oil) | **✓** | **✓** | ✘ | ✘ |
| Hanai et al., 2012^57^ | Parallel RCT | Japan | 95 (25 F, 70 M) adults (19-48 y) with CD in remission (CDAI≤150) | Stable doses of 5-ASA (90.5%), and SASP (9.5%) started at least 8 weeks prior to study enrolment | Elemental (Elental) | **✓** | ✘ | **✓** | ✘ | ✘ | ✘ |
| Sazuka et al., 2012^58^ | Retrospective | Japan | 74 (18 F, 56 M) adults (18-60 y) in remission (CDAI<150) achieved with IFX | 5-ASA (96%), steroids (9%), AZA (7%), 6-MP (4%) | Elemental (Elental) and/or polymeric (Racol) | ✘ | ✘ | **✓** | ✘ | ✘ | ✘ |
| Gupta et al., 2013^26^ | Retrospective | US | 43 (14 F, 29 M) children (5.1-17.4 y) with active CD (PCDAI>10) | 5-ASA, antibiotics, steroids, AZA, 6-MP, methotrexate, ADA, IFX | Elemental (Optimental, Neocate), semi-elemental (Peptamen AF, Peptamen 1.5, Peptamen 1.0, Nutren, Peptamen jr 1.5) or polymeric (Modulen IBD, Boost) | ✘ | ✘ | **✓** | **✓** | ✘ | ✘ |
| Hirai et al., 2013^60^ | Retrospective | Japan | 102 (25 F, 77 M) adults with CD in remission (CRP<0.3mg/dL) achieved with IFX | Prednisone (13.7%), AZA (26.5%) | Elemental (Elental) or semi-elemental | ✘ | ✘ | ✘ | **✓** | ✘ | ✘ |
| Yamamoto et al., 2013^55^ | Secondary outcome analysis to open-label controlled trial (Yamamoto et al., 2007b) | Japan | 40 (14 F, 26 M) adults (mean (SE): 31 (3.7) y PEN group; 33 (3.9) y control group) with CD in remission (CDAI<150) achieved with surgery | No patients received steroids, immunomodulators or IFX prior to clinical recurrence | Elemental (Elental) | **✓** | Clinical interview | **✓** | ✘ | ✘ | **✓** |
| Duncan et al., 2014^61^ | Retrospective | UK | 48 children (2.5-15.91 y) with CD in remission (PGA) achieved with EEN | No other treatments | Polymeric (Modulen IBD, Fortsip, Neocate Advance) | **✓** | Review of medical records | **✓** | ✘ | ✘ | ✘ |
| Kamata et al., 2014^62^ | Retrospective | Japan | 125 (24 F, 121 M) adults (mean (SE): 36.4 (1.0) y PEN (≥900kcal/day) group; 35.9 (1.0) y PEN (<900kcal/day) group) with CD in remission on maintenance IFX | Immunomodulators (24%) | Elemental | ✘ | ✘ | ✘ | ✘ | ✘ | ✘ |
| Sigall Boneh et al., 2014^27^ | Retrospective | Israel | 47 patients: 34 (10 F, 24 M) children (6-18 y) with active CD (PCDAI≥10), 13 (7 F, 6 M) adults (19-32 y) with active CD (HBI>3) | Immunomodulators (55.3%) | Polymeric (Modulen IBD, Pediasure) | ✘ | Not specified | **✓** | **✓** | ✘ | ✘ |
| Sökülmez et al., 2014^28^ | Pragmatic RCT | Turkey | 38 hospitalised malnourished adults with IBD (25 with UC; 13 (3 F, 10 M) adults (mean (SD): 31.0 (8.5) y PEN group; 43.0 (18.6) y control group) with active CD (CDAI≥220)) | No information | Polymeric (Novasource GI Control) | **✓** | Observing food consumption in hospital | **✓** | ✘ | ✘ | ✘ |
| Kang et al., 2015^29^ | Open-label controlled trial | Korea | 34 (15 F ,19 M) children (mean (SD): 14.1 (3.8) y PEN group; 13.8 (4.1) y control group) with severe CD (PCDAI>45) after 8 weeks of drug-based induction therapy | IFX (35.3%) | Polymeric (Ensure) | **✓** | ✘ | **✓** | ✘ | ✘ | ✘ |
| Konno et al., 2015^63^ | Retrospective | Japan | 58 (23 F, 35 M) children with CD in remission (PCDAI<10 + SES-CD<2) achieved with EEN + 5-ASA or TPN + 5-ASA | 5-ASA (100%) | Elemental (Elental) | ✘ | Not specified | **✓** | ✘ | ✘ | ✘ |
| Lee et al., 2015^30^ | Prospective observational study with a control group | US and Canada | 90 (36 F, 54 M) children (3.8-19.5 y) with active CD (PCDAI>10) | Antibiotics (28.9%), oral 5-ASA (50%), rectal 5-ASA (2.2%), thiopurines (8.9%), methotrexate (6.7%), systemic steroids (36.7%), rectal steroids (1.1%) | Semi-elemental for PEN (Peptamen JR, Peptamen 1.5)  Polymeric for EEN (Modulen IBD, Osmolite) | **✓** | 3-day multiple pass 24h dietary recall | **✓** | ✘ | **✓** | ✘ |
| Kim et al., 2015^64^ | Retrospective | Korea | 58 (9 F, 49 M) children (10-16 y) with CD in remission (PCDAI<10) achieved with EEN | No immunomodulators or IFX (eligibility criteria); no information about other concomitant treatments | Elemental (Energen, Monowell) | ✘ | Review of medical records | **✓** | ✘ | ✘ | ✘ |
| Nakano et al., 2017^31^ | Open-label uncontrolled trial | Japan | 25 (19 F, 6 M) adults (mean (SD): 29.08 (7.72) y) with active CD (CDAI≥150) | 5-ASA (24%), thiopurines (16%), anti-TNFα (40%: anti-TNFα maintenance doses at baseline; 36%: new anti-TNFα treatment started during treatment) | Elemental (Elental) | ✘ | ✘ | **✓** | **✓** | ✘ | ✘ |
| Ohara et al., 2017^65^ | Retrospective | Japan | 38 (8 F, 30 M) adults (22-72 y) with CD after intestinal resection with anastomosis | SASP/5-ASA (84%), AZA/6-MP (16%), anti-TNFα (55%) | Elemental | ✘ | Not specified | **✓** | ✘ | ✘ | **✓** |
| Schulman et al., 2017^70^ | Retrospective | Israel | 87 (26 F, 61 M) children (median (IQR): 11.5 (9.2-13.7) y PEN group; 14.7 (12.7-16.3) y control group) with CD in remission (PGA) | Steroids (70.1%), thiopurines (74.7%), anti-TNFα (27.6%), methotrexate (10.3%), antibiotics (43.7%) | Polymeric (Modulen IBD, Ensure, PediaSure) | **✓** | ✘ | **✓** | **✓** | ✘ | ✘ |
| Sigall Boneh et al., 2017^32^ | Retrospective | Israel | 21 (9 F, 12 M) patients (11 adults, 10 children; (mean (SD): 22.2 (8.9) y) with LOR to IFX and/or ADA and evidence for active inflammation (by CRP, FC, or colonoscopy) | ADA and/or IFX dose escalation (81%), ADA and/or IFX dose interval escalation (76%), no dose escalation (23%); combination therapy (81%): AZA (24%), methotrexate (33%), previous AZA (14%), previous methotrexate (10%), previous 6-MP (5%) | Polymeric (Modulen IBD, Pediasure) | ✘ | Clinical interview | **✓** | **✓** | ✘ | ✘ |
| Gavin et al., 2018^66^ | Retrospective | UK | 102 (24 F, 78 M) children (2-16 y) with CD in remission (PGA) achieved with EEN or steroids +/- PEN | 5-ASA (5%), thiopurines (60%), biologics (25%), methotrexate (4%), surgery within last 12 months (3%), no other treatments (8%) | Polymeric (Modulen IBD, Fortsip Compact, Fortijuice) or elemental (EO28) | **✓** | Not specified | **✓** | ✘ | ✘ | ✘ |
| Hisamatsu et al., 2018^9^ | Parallel RCT | Japan | 20 (4 F, 16 M) adults (mean (SD): 34.8 (8.81) y PEN group; 35.2 (10.11) y control group) with non-colonic CD and LOR to IFX (5 mg/kg every 8 weeks) | Immunomodulators (35%), steroids (5%), 5-ASA (85%) | Elemental | **✓** | ✘ | **✓** | ✘ | ✘ | ✘ |
| Logan et al., 2018^67^ | Prospective observational study with a control group | UK | 66 (24 F ,42 M) children (median (IQR): 13.4 (10.7-14.9) y) with active CD treated with EEN: 41/66 children achieved remission (wPCDAI<12.5); 29/41 children advised to continue with PEN | AZA (59%) | Polymeric (Modulen IBD) | **✓** | 3-day food diary | **✓** | ✘ | **✓** | ✘ |
| Sugita et al., 2018^17^ | Retrospective | Japan | 117 (30 F, 87 M) adults (median (IQR): 36 (33-43) y PEN (≥900 kcal/day) group; 37.5 (27.75-43) y PEN (<900 kcal/day) group) with CD in remission (HBI<5) achieved with ADA | Immunomodulators (30%), prednisolone (3%) | Elemental | ✘ | Clinical interview | **✓** | **✓** | ✘ | ✘ |
| Wall et al., 2018^33^ | Open-label controlled trial | New Zealand | 38 (30 F, 8 M) adults (15.8-38.5 y) with active CD (by endoscopy/ radiology/ elevated FC) | No other treatments (42.1%), 5-ASA (39.5%), immunomodulators (15.8%), biologics (5.3%) | Polymeric (Ensure Plus) | **✓** | 3-day food diary | **✓** | **✓** | **✓** | ✘ |
| Agin et al., 2019^34^ | Retrospective | Turkey | 35 (13 F, 22 M) children (10-17.8 y) with active CD (PCDAI≥10+ clinical symptoms) | Steroids (2 mg/kg/day, max 60 mg/day; 100%), antibiotics | Polymeric (Modulen IBD) | **✓** | ✘ | **✓** | **✓** | ✘ | ✘ |
| Hirai et al., 2019^59^ | Open-label controlled trial | Japan | 72 (20 F, 52 M) anti-TNFα-naïve adults (18-70 y) with CD in remission (CDAI<200) achieved with anti-TNFα (IFX/ADA) | AZA (12.5%) | Elemental (Elental) | **✓** | Clinical interview | **✓** | **✓** | ✘ | ✘ |
| Levine et al., 2019^10^ | Parallel RCT | Israel and Canada | 78 (32 F, 46 M) children (mean (SD): 13.8 (2.8) y PEN+CDED group; 14.5 (2.6) y EEN group) with active mild to moderate CD (10≤PCDAI≤40) + inflammation (CRP>5 g/L / ESR>20mm/h / FC>200μg/g) | Immunomodulators (9.5%) | Polymeric (Modulen IBD) | **✓** | Clinical interview + 3-day food diary + modified MARS questionnaire | **✓** | **✓** | **✓** | ✘ |
| Sigall Boneh et al., 2019^36^ | Secondary outcome analysis to a parallel RCT (Levine et al., 2019) | Israel and Canada | 73 (27 F, 46 M) children (mean (SD): 14.2 (2.7) y) with active mild to moderate CD (10≤PCDAI≤40) + inflammation (CRP>5 g/L / ESR>20mm/h / FC>200μg/g) | Immunomodulators (9.5%) | Polymeric (Modulen IBD) | **✓** | Clinical interview + 3-day food diary + modified MARS questionnaire | **✓** | **✓** | ✘ | ✘ |
| Urlep et al., 2019^37^ | Open-label controlled trial | Slovenia | 25 (13 F, 12 M) children (3.6-18.0 y) with active CD (PCDAI>10 + SES-CD>3) | No changes in maintenance treatment in the last 3 months; immunomodulators only (32%), anti-TNF (4.5%), vedolizumab (4.5%) | Polymeric (Alicalm) | **✓** | Clinical interview | **✓** | **✓** | **✓** | **✓** |
| Bruckner et al., 2020^22^ | Open-label controlled trial | Germany | 41 (19 F, 22 M) children (mean (SD): 15.0 (1.9) y PEN group; 12.8 (3.1) y control group) with CD in remission (wPCDAI<12.5) or with mild CD (12.5≤wPCDAI≤40) | AZA (51%), 5-ASA (27%), Methotrexate (15%), IFX (63%), ADA (2%) | Polymeric (Modulen IBD) | **✓** | 3-day food diary | **✓** | **✓** | **✓** | ✘ |
| Ferreira et al., 2020^35^ | Open-label controlled trial | Brazil | 38 (15 F, 23 M) adults (18-69 y) with active CD (CDAI≥150/ SES-CD≥3/ RS≥i2 for patients after surgery) | Prednisone, AZA, 6-MP, methotrexate, 5-ASA, ciproflaxacin | Polymeric (Modulen IBD or Nutren) | **✓** | 3-day food diary or diet history | **✓** | **✓** | ✘ | **✓** |
| Ferreiro et al., 2021^42^ | Open-label uncontrolled trial | Spain | 144 (67 F, 77 M) malnourished adults (mean (SD): 50 (18) y) with active CD (HBI>5) | Corticosteroids (70.1%), immunosuppressants (29.9%), biologics (31.9%), 5-ASA (32.6%), antibiotics (21.5%), other treatments (10.0%) | Semi-elemental (brand name not provided) | ✘ | Clinical interview | **✓** | **✓** | ✘ | ✘ |
| Niseteo et al., 2021^38^ | Retrospective | Croatia | 61 (30 F, 31 M) children (6.7-17.9 y) with active CD (wPCDAI≥12.5) | No other treatments (18%), AZA (79%), 5-ASA (1.6%) | Polymeric (PEN+CDED group: Modulen IBD, PediaSure, NutriniDrink MF, Resource Junior, Resource 2.0; EEN group: Modulen IBD, PediaSure, Osmolite, Ensure Plus, Resource Junior) | **✓** | ✘ | **✓** | **✓** | ✘ | ✘ |
| Shinozaki et al., 2021^68^ | Retrospective | Japan | 37 (10 F, 27M) adults (19-69 y) with CD after first surgery | Thiopurines (AZA, 6-MP; 38%), anti-TNFα (IFX, ADA; 51%) | Elemental | ✘ | ✘ | ✘ | ✘ | ✘ | ✘ |
| Szczubełek et al., 2021^39^ | Open-label uncontrolled trial | Poland | 32 (18 F, 14 M) adults (20-62 y) with active CD (CDAI>150) | No information | Polymeric (Modulen IBD) | ✘ | ✘ | **✓** | **✓** | **✓** | ✘ |
| Davidson et al., 2022^69^ | Retrospective | US | 44 children and young adults (3-20 y) with CD in remission successfully achieved with 90% PEN | No information | Polymeric (brand name not provided) | **✓** | Not specified | **✓** | **✓** | **✓** | ✘ |
| Matuszczyk et al., 2022^18^ | Open-label uncontrolled trial | Poland | 48 (21 F, 27 M) children (4-17 y) with active CD (FC≥250 µg/g) | No information | Polymeric (Modulen IBD) | ✘ | Clinical interview | **✓** | **✓** | **✓** | ✘ |
| Yanai et al., 2022^40^ | Parallel RCT | Israel | 40 (22 F, 18 M) biologic-naïve adults (median (IQR): 30 (24-39) y) with mild-to-moderate (5<HBI<14) active CD (by colonoscopy/imaging with CRP>5 mg/L or FC>200 μg/g) | 5-ASA (10% (4/40) patients: 2 stopped and other 2 continued on stable doses) | Polymeric (Modulen IBD) | **✓** | 3-day food diary + 24-hour dietary recall + modified MARS questionnaire | **✓** | **✓** | **✓** | **✓** |
| Arcucci et al., 2023^19^ | Parallel RCT | Argentina | 21 (8 F, 13 M) children (median (IQR): 10.7 (4.6-14.5) y PEN+CDED group; 13.6 (12.7-14.4) y control group) with CD and elevated FC levels (FC≥250 µg/g) on maintenance biologic therapy | 5-ASA (62%), prednisolone (24%), IFX (24%), ADA (71%), vedolizumab (5%) | Polymeric (Modulen IBD) | **✓** | 5-day food diary + clinical interview | **✓** | **✓** | **✓** | ✘ |
| Fliss-Isakov et al., 2023^21^ | Retrospective | Israel | 96 (55 F, 41 M) adults: 72 adults (mean (SD): 35.3 (15.2) y) with active CD (HBI≥5 or FC≥250 µg/g or SES-CD≥7 or RS≥i2); and 24 adults (37.8 (16.9) y) with CD in remission | Current biologic therapy (44%), concomitant standard of care treatments (no more information) | No information provided | ✘ | 24h dietary recall + clinical interview | **✓** | **✓** | **✓** | ✘ |
| Jijón Andrade et al., 2023^43^ | Retrospective | Spain | 15 (7 F, 8 M) children with mild-to-moderate CD (PCDAI: 12.5-47.5): 9 children (median (IQR): 11.8 (10.7-14.9) y) who were treatment naïve; and 6 children (median (IQR): 13.5 (9.1-15.4) y) with LOR to biologic therapy | AZA (73%), ADA (20%), ustekinumab (20%) | Polymeric (Modulen IBD) | ✘ | Clinical interview | **✓** | **✓** | **✓** | ✘ |

*Age is presented as range unless stated otherwise.

Abbreviations used: 5-ASA: Aminosalicylates; 6-MP: 6-Mercaptopurine; ADA: Adalimumab; AZA: Azathioprine; CD: Crohn’s Disease; CDAI : Crohn’s Disease Activity Index; CDED: Crohn’s Disease Exclusion Diet; CRP: C-Reactive Protein; EEN: Exclusive Enteral Nutrition; ESR: Erythrocyte Sedimentation Rate; F: Female; FC: Faecal Calprotectin; GI: Gastrointestinal; HBI: Harvey-Bradshaw Index; IBD: Inflammatory Bowel Disease; IFX: Infliximab; IQR: Interquartile Range; LOR: Loss of Response; M: Male; MARS: Medication Adherence Report Scale; PCDAI: Paediatric Crohn’s Disease Activity Index; PEN: Partial Enteral Nutrition; PGA: Physician Global Assessment; RCT: Randomised Control Trial; RS: Rutgeerts Score; SASP: Sulfasalazine; SD: Standard Deviation; SE: Standard Error; SEM: Standard Error of Mean; SES-CD: Simple Endoscopic Score for Crohn’s Disease; TNFα: Tumour Necrosis Factor Alpha; TPN: Total Parenteral Nutrition; wPCDAI: Weighted Paediatric Crohn’s Disease Activity Index; Y: Years.

**Supplementary Table 3:** Summary of study results investigating the clinical efficacy of partial enteral nutrition alongside unrestricted diet as induction treatment in patients with active Crohn’s disease. Results are presented at the end of intervention/follow-up duration unless states otherwise. Values in bold represent data for patients receiving partial enteral nutrition.

| **Study reference** | **Intervention description** | **Comparison description** | **Duration*** | **Response/remission criteria** | **Results** |
| --- | --- | --- | --- | --- | --- |
| **Johnson et al., 2006^8^** Parallel RCT | 50% PEN (n=26) | EEN (n=24) | 6 weeks | Remission: PCDAI <10 | Remission rates: **15%** **(4/26)** PEN group, 42% (10/24) EEN group (PEN vs EEN: P=0.035) PCDAI mean (95% CI): ↓by **13 (-7, -19)** PEN group (P=0.001), ↓by 26 (-19, -33) EEN group (P=0.001) (PEN vs EEN: P=0.005) ESR mean (95% CI): ↓by **4 (-17, 8) mm/h** PEN group (NS), ↓by 26 (-42, -10) mm/h EEN group (P=0.004) (PEN vs EEN: P=0.003) Albumin mean (95% CI): ↑by **1.7 (-0.1, 3.5)** **g/L** PEN group (NS), ↑by 5 (3, 7) g/L EEN group (P<0.001) (PEN vs EEN: P=0.019) Platelet count mean (95% CI): ↓by **26 (-81, 29) x10^9^/L** PEN group (NS), ↓by 146 (-210, -82) x10^9^/L EEN group (P<0.001) (PEN vs EEN: P=0.006) Haemoglobin mean (95% CI): ↓by **0.3 (-6, 0) g/dL** PEN group (NS), ↑by 0.8 (0.3, 1.3) g/dL EEN group (P=0.011) (PEN vs EEN: P<0.001) ↓CRP mean (95% CI): by **6 (-26, 14)** **mg/L** PEN group (NS) vs 13 (-47, 21) mg/L EEN group (NS) (PEN vs EEN: NS) |
| **Hartman et al., 2008^20^** Retrospective | 35-50% PEN with Modulen IBD (n=28)  35-50% PEN with Ensure Plus (n=18) | Unrestricted diet (n=18) | ~4.5-5.5 months | Remission: PCDAI<15 | Remission rates: **57.1% (16/28)** Modulen group, **22.2% (4/18)** Ensure Plus group, 22.2% (4/18) control group (Modulen group vs Ensure plus and control groups: P=0.03) PCDAI mean (SD): ↓from **35 (17) to 15 (13)** Modulen group (P=0.0001), ↓from **31 (11) to 22 (7)** Ensure Plus group (P=0.02), from 27 (10) to 21 (12) control group (NS) (Modulen vs control: P=0.02) ESR mean (SD): ↓from **33 (17) to 22 (17) mm/h** Modulen group (P=0.03), from **33 (15) to 25 (10)** **mm/h** Ensure Plus group (NS), from 32 (17) to 26 (19) mm/h control group (NS) Haematocrit mean (SD): from **34 (4) to 37 (4)** Modulen group (NS), from **32 (3) to 34 (3)** Ensure Plus group (NS), from 35 (3) to 35 (2) control group (NS) Albumin mean (SD): from **3.6 (0.5) to 3.9 (0.5) g/dL** Modulen group (NS), from **3.6 (0.4) to 3.8 (0.3) g/dL** Ensure Plus group (NS), from 3.7 (0.4) to 3.8 (0.3) g/dL control group (NS) |
| **Wiese et al., 2011^23^** Open-label uncontrolled trail | PEN at 600 kcal/day with formula enriched with fish oil, prebiotics, and antioxidants (n=28) | ✘ | 4 months | Response and remission rates not assessed | 71.4% (20/28) patients completed the study (per protocol analysis): 50% (10/20) patients had serum EPA>2.0% indicating compliance (compliant group), and 50% (10/20) had EPA<2.0% indicating poor compliance (non-compliant group). CDAI median (IQR): ↓by **45.6 (-61.4--7.4)** all patients (NS), ↓by **47.8 (-65--37.8)** compliant group (P=0.049), ↓by 8.1 (-54.6-40.1) non-compliant group (NS)  CRP median (IQR): ↑by **0.4 (-0.8-1.8) mg/dL** all patients (NS), ↑by **0.3 (-0.4-0.0)** **mg/dL** compliant group (NS), ↑by 0.3 (0.0-1.6) mg/dL non-compliant group (NS) |
| **Gupta et al., 2013^26^** Retrospective | 80-90% PEN overnight via NGT (n=43) | ✘ | 8-12 weeks | Response: ↓PCDAI≥12.5 Remission: PCDAI<10/aPCDAI* <10 | Response rate: **87% (20/23)**  Remission rate: **65% (15/23)**  PCDAI mean: ↓from **26.9 to 10.2** (P<0.0001)  aPCDAI* mean: ↓from **17.6 to 5.4** (P<0.0001) CRP mean (n=21): ↓from **2.8 to 1.7 mg/L** (P<0.02)  ESR mean (n=25): ↓from **33.5 to 21.1 mm/h** (P<0.0001) Albumin mean (n=27): ↑from **3.7 to 3.9 g/dL** (P<0.03) *PCDAI calculated for 23 patients, aPCDAI (PCDAI without blood inflammatory biomarkers) calculated for all patients. |
| **Sökülmez et al., 2014^28^** Pragmatic RCT | PEN at ≥500 kcal/day 3 consecutive days/week (n=6) | Unrestricted diet (deficiencies supported by adjusting the hospital food, n=7) | 3 weeks | Remission: CDAI<150 | Remission rates: **33.3% (2/6)** PEN group, 57.1% (4/7) control group CDAI: ↓PEN group (P=0.024), ↓control group P=0.001) (PEN vs control: NS) (values not presented) |
| **Kang et al., 2015^29^** Open-label controlled trail | PEN at 20kcal/kg (n=17) | Unrestricted diet (n=17) | 4 weeks | Response and remission rates not assessed | Results after one year from PEN initiation. PCDAI mean (SD): ↓by **62.7 (16.4)** PEN group (P<0.001), ↓by 50.2 (17.1) control group (P<0.001) (PEN vs control: P<0.001) |
| **Lee et al., 2015^30^** Prospective observational study with a control group | 50% PEN (n=16) | EEN (n=22)  Anti-TNFα (n=52) | 8 weeks | Response: ↓PCDAI≥15 / PCDAI≤10  Remission: PCDAI≤10 FC response: FC≤50 µg/g for patients with baseline FC>50 µg/g (0 patients from PEN group), FC≤250 µg/g for patients with baseline FC>250 µg/g | Response rates: **64%** PEN group, 88% EEN group, 84% anti-TNFα group (PEN vs anti-TNFα: P=0.04, PEN vs EEN: NS) Remission rates: **50%** PEN group, 76% EEN group, 73% anti-TNFα group (PEN vs anti-TNFα: NS, PEN vs EEN: P=0.03) PCDAI mean (SD): ↓by **17.7 (20.3)** PEN group (P<0.05), ↓by 25.5 (17) EEN group (P<0.05), ↓by 19.5 (15.5) anti-TNFα group (P<0.05) (PEN vs anti-TNFα: NS, PEN vs EEN: NS) FC≤50 µg/g: **0% (0/0)** PEN group, 5% EEN group, 30% anti-TNFα group  FC≤250 µg/g: **14%** PEN group, 45% EEN group, 62% anti-TNFα group (PEN vs anti-TNFα: P=0.02, PEN vs EEN: P=0.04) FC>50% baseline reduction: **47%** PEN group, 64% EEN group, 72% anti-TNFα group (PEN vs anti-TNFα: P=0.02, PEN vs EEN: NS) FC mean (SD): ↓by **380 (660) µg/g** PEN group (P<0.05), ↓by 682 (678) µg/g EEN group (P<0.05), ↓by 622 (678) µg/g anti-TNFα group (P<0.05) (PEN vs anti-TNFα: NS, PEN vs EEN: NS) |
| **Nakano et al., 2017^31^** Open-label uncontrolled trial | PEN mean (SD): 4.92 (1.04) packs/day (300 kcal/pack) (n=25) | ✘ | 12 weeks | Remission: CDAI<150 | Remission rate: **68% (17/25)**  CDAI mean (SD): ↓from **236.1 (99.8) to 134.4 (78.4)** (P<0.001) CRP mean (SD): ↓from **2.1 (2.3) to 1.0 (1.8)** **mg/dL** (P=0.007) Albumin mean (SD): ↑from **3.6 (0.6) to 3.9 (0.5)** **g/dL** (P=0.002) |
| **Wall et al., 2018^33^** Open-label controlled trial | EEN for first 2 weeks + PEN (one small meal allowed) for next 6 weeks (n=13) | EEN (n=25) | 8 weeks | Response and remission rates not assessed | HBI median: ↓from **5 to 3** in all patients after 2 weeks (P=0.003), improvements sustained at week 8 in PEN group (values not presented), further improvements at week 8 in EEN group (values not presented, P=0.031) (PEN vs EEN: NS) CRP median: ↓from **10 to 5 mg/L** in all patients after 2 weeks (P=0.005), improvements sustained at week 8 in PEN and EEN groups (values not presented) (PEN vs EEN: NS) Albumin median: from **39.5 to 39.5 g/L** in all patients after 2 weeks (NS), values not presented at week 8 (PEN vs EEN: NS) FC median: ↓from **927 to 674 µg/g** in all patients after 2 weeks (P=0.028), improvements sustained at week 8 in EEN and PEN group (values not presented) (PEN vs EEN: NS) FC<500 µg/g: **22% (2/9)** PEN group, 36% (5/14) EEN group at week 8 |
| **Agin et al., 2019^34^** Retrospective | 50% PEN (n=16) | Unrestricted diet (n=19) | 8 weeks | Remission: PCDAI<10 + absence of clinical symptoms | Remission rates: **6% (1/16)** PEN group, 0% (0/19) control group after 1 month (PEN vs control: NS); **88% (14/16)** PEN group, 42% (8/19) control group after 3 months (PEN vs control: P=0.001); **100% (16/16)** PEN group, 100% (19/19) control group after 6 months (PEN vs control: NS) PCDAI mean (SD): from **54.2 (7) to 48 (6.8)** after 1 week **to 16.7 (4.8)** after 1 month **to 7.5 (2.5)** after 3 months **to 7.2 (3.5)** after 6 months PEN group, from 44.4 (10.3) to 40 (8.7) after 1 week to 34.8 (7.6) after 1 month to 23 (5.5) after 3 months to 8.7 (3.6) after 6 months (PEN vs control: baseline: P=0.003, 1 week: P=0.006, 1 month: P=0.001, 3 months: P=0.001, 6 months: NS) CRP mean (SD): from **5.4 (6.1) to 2.3 (2)** after 1 week **to 2.8 (3.9)** after 1 month **to 1 (1.9)** after 3 months **to 0.3 (0.2) g/L** after 6 months PEN group, from 2.9 (3.4) to 1.4 (1.7) after 1 week to 1 (2.1) after 1 month to 0.6 (0.7) after 3 months to 0.9 (1.4) g/L after 6 months control group (PEN vs control baseline: NS, 1 week: NS, 1 month: NS, 3 months: NS, 6 months: NS) ESR mean (SD): from **57 (26) to 42 (28)** after 1 week **to 19.8 (13)** after 1 month **to** **8.7 (5.8)** after 3 months **to 6 (3.8) mm/h** after 6 months PEN group, from 43.8 (17) to 33.6 (12.9) after 1 week to 29.9 (13) after 1 month to 8.8 (2.4) after 3 months to 6.2 (3.6) mm/h after 6 months control group (PEN vs control baseline: NS, 1 week: NS, 1 month: P=0.037, 3 months: NS, 6 months: NS) Albumin mean (SD): from **3.1 (0.7) to 3.4 (0.6)** after 1 week **to 3.7 (0.5)** after 1 month **to 3.8 (0.4)** after 3 months **to 4.1 (0.3) g/dL** after 6 months PEN group, from 3.8 (0.6) to 3.87 (0.4) after 1 week to 4 (0.4) after 1 month to 4 (0.4) after 3 months to 4.2 (0.4) g/dL after 6 months control group (PEN vs control baseline: P=0.007, 1 week: P=0.009, 1 month: P=0.018, 3 months: NS, 6 months: NS) Haemoglobin mean (SD): from **10.7 (1.8) to 16.7 (23)** after 1 week **to 11.6 (2)** after 1 month **to 11.3 (2)** after 3 months **to 11.9 (1.8) g/dL** after 6 months PEN group, from 12.1 (2.3) to 12.2 (1.9) after 1 week to 12.6 (2.3) after 1 month to 13.3 (1.8) after 3 months to 13 (2) g/dL after 6 months (PEN vs control baseline: P=0.021, 1 week: NS, 1 month: NS, 3 months: P=0.010, 6 months: NS)  Platelet count mean (SD): from **425 (13) to 416 (109)** after 1 week **to 407 (112)** after 1 month **to 388 (129)** after 3 months to **385 (137) × 10^3^/µL** after 6 months PEN group, from 363 (180) to 344 (113) after 1 week to 321 (86) after 1 month to 334 (123) after 3 months to 325 (78) × 10^3^/µL after 6 months control group (PEN vs control baseline: P=0.03, 1 week: P=0.03, 1 month: P=0.018, 3 months: NS, 6 months: NS)  Leucocyte count mean (SD): from **9.3 (2.4) to 10.1 (3.3)** after 1 week **to 12.4 (5.6)** after 1 month **to 10.5 (5.5)** after 3 months **to 9.5 (3.7) × 10^3^/µL** after 6 months PEN group, from 9.3 (4.7) to 10.2 (0.3) after 1 week to 11.6 (6.4) after 1 month to 8.4 (2.8) after 3 months to 8.1 (3.1) × 10^3^/µL after 6 months control group (PEN vs control baseline: NS, 1 week: NS, 1 month: NS, 3 months: NS, 6 months: NS) |
| **Ferreira et al., 2020^35^** Open-label controlled trial | 50% PEN with Nutren + nutrition counselling (n=13)  50% PEN with Modulen IBD + nutrition counselling (n=13) | Unrestricted diet + nutrition counselling (n=12) | 3 months | Response and remission rates not assessed | CDAI mean (SD): ↓from **164.3 (14.1) to 121.5 (20.4)** Nutren group (P=0.01), ↓from **168.8 (17.1) to 87.4 (21.3)** Modulen group (P=0.04), from 121.9 (29.4) to 101.4 (18.6) control group (NS) (between-group: NS) CRP mean (SD): from **52.5 (26.7) to 13.9 (4) mg/dL** Nutren group (NS), ↓from **41.6 (18.6) to 5.9 (2.1) mg/dL** Modulen group (P=0.01), from 11.1 (3.3) to 7.8 (3.1) mg/dL control group (NS) (between-group: NS) Histological improvements: **38.5%** Nutren group (NS), **100%** Modulen group (P<0.01), 40% control group (NS) SES-CD median (range): from **6 (0-36) to 3.5 (0-23)** Nutren group (NS), from **10.5 (0-36) to 9.5 (0-23)** Modulen group (NS), from 6 (0-34) to 3 (0-23) control group (NS) (between-group: NS) |
| **Ferreiro et al., 2021^42^** Open-label uncontrolled trial | Supplementary PEN provided at 200, 400 or 600 kcal/day (dose dependent on malnutrition severity) (n=144) | ✘ | 12 weeks | Remission: HBI<5 | Remission rate: **71.8%** HBI mean: ↓from **10.2 to 3.7** (P<0.001) Albumin mean: ↑from **3.0 to 3.7 g/dL** (P<0.001) |

*Duration is presented for the duration of intervention unless stated otherwise.

Abbreviations used: 95% CI: 95% Confidence Intervals; aPCDAI: Abbreviated Paediatric Crohn’s Disease Activity Index; CDAI: Crohn’s Disease Activity Index; CRP: C-Reactive Protein; EEN: Exclusive Enteral Nutrition; EPA: Eicosapentaenoic Acid; ESR: Erythrocyte Sedimentation Rate; FC: Faecal Calprotectin; HBI: Harvey-Bradshaw Index; IQR: Interquartile Range; NGT: Nasogastric Tube; NS: Not Significant; PCDAI: Paediatric Crohn’s Disease Activity Index; PEN: Partial Enteral Nutrition; RCT: Randomised Control Trial; SD: Standard Deviation; SES-CD: Simplified Endoscopic Score for Crohn’s Disease; TNFα: Tumour Necrosis Factor Alpha.

**Supplementary Table 4:** Summary of study results investigating the clinical efficacy of partial enteral nutrition as maintenance treatment in patients with Crohn’s disease in remission achieved with non-biological treatments. Results are presented at the end of intervention/follow-up duration unless states otherwise. Values in bold represent data for patients receiving partial enteral nutrition.

| **Study reference** | **Intervention description** | **Comparison description** | **Duration*** | **Remission/relapse criteria** | **Results** |
| --- | --- | --- | --- | --- | --- |
| **Aiges et al., 1989^45^** Open-label controlled trial | Supplementary PEN at 1,000-1,500 kcal/day overnight via NGT (n=8) | Unrestricted diet (n=6) | 1 year | Remission and relapse rates not assessed | Lloyd-Still score mean (SD): ↑from **70.8 (3.8) to 81.5 (5.9)** PEN group (P<0.001), from 59.6 (10.8) to 57.7 (13.5) control group (NS) |
| **Wilschanski et al., 1996^46^** Retrospective | 50-60% PEN overnight via NGT for 4-5 nights/week (n=28) | Unrestricted diet (n=19) | 1 year | Relapse: PCDAI>20 + need for additional treatment | Relapse rates: **17.9% (5/28)** PEN group, 78.9% (15/19) control group, after 6 months (PEN vs control: P<0.001); **42.9% (12/28)** PEN group, 78.9% (15/19) control group, after 12 months (PEN vs control: P<0.02) |
| **Verma et al., 2000^48^** Open-label controlled trial | 35-50% PEN (n=21) | Unrestricted diet (n=18) | 1 year | Remission: CDAI<150 + absence of GI symptoms  Relapse: ↑CDAI>100 / CDAI>150 / need for surgery / need for steroid dose escalation (>20 mg/day) | Remission rates: **48% (10/21)** PEN group, 22% (4/18) control group, using ITT analysis (PEN vs control: P<0.0003); **60% (10/17)** PEN group, 22% (4/18) control group, using per protocol analysis (PEN vs control: P<0.00001) CDAI mean (SEM): no change from **112.8 (11.5)** PEN group (NS) (post-treatment values not presented), from 88.5 (10.6) to 80 (6.1) control group (NS) CRP mean (SEM): ↓from **9 (1.9) mg/L** PEN group (post-treatment and P values not presented), from 10.7 (5.1) to 8.1 (3.7) mg/L control group (NS) Albumin mean (SEM): no change from **40.6 (1.7) g/L** PEN group (NS) (post-treatment values not presented), from 41.5 (1) to 41.2 (1) g/L control group (NS) |
| **Verma et al., 2001^47^** Parallel RCT | 35-50% elemental PEN (n=19)  35-50% polymeric PEN (n=14) | ✘ | 1 year | Remission: CDAI≤150 2 weeks prior to study initiation + absence of GI symptoms + ESR<20 mm/h  Relapse: ↑CDAI>100 / CDAI≥200 / failure to withdraw steroids / need for surgery / need for steroid dose escalation (≥20 mg/day) | Remission rates: **43% (14/33): 42% (8/19)** elemental PEN group, **43% (6/14)** polymeric PEN group, using ITT analysis (elemental vs polymeric: NS); **52% (14/27),** using per protocol analysis CDAI mean (SEM): no change from **106.4 (14.9)** elemental PEN group (NS), no change from **90.4 (17.8)** polymeric PEN group (NS) (post-treatment values not presented)  CRP mean (SEM): no change from **16.6 (5.3) mg/L** elemental PEN group (NS), no change from **10.3 (2.7) mg/L** polymeric PEN group (NS) (post-treatment values not presented)  ESR mean (SEM): no change from **14.1 (2.7) mm/h** elemental PEN group (NS), no change from **12.5 (3.0) mm/h** polymeric PEN group (NS) (post-treatment values not presented)  Albumin mean (SEM): no change from **40.4 (0.8) g/L** elemental PEN group (NS), no change from **39.7 (0.9) g/L** polymeric PEN group (post-treatment values not presented) |
| **Knight et al., 2005^15^** Retrospective | PEN at 1,000 mL/day (n=22) | Unrestricted diet (n=18) | Median follow-up (range):  3 (1-7) years | No information | Relapse rates: **59% (13/22)** PEN group, 67% (12/18) control group (PEV vs control: NS) |
| **Day et al., 2006^16^** Retrospective | PEN at 500-1,000 mL/day (n=4) | Unrestricted diet + 5-ASA and/or AZA (n=8) | Mean follow-up (range): 15.2 (10-21) months | Remission: PCDAI≤15 | Remission rates: **100% (4/4)** PEN group, 62.5% (5/8) control group |
| **Esaki et al., 2006^50^** Retrospective | PEN at ≥1,200 kcal/day (n=98)  PEN at <1,200 kcal/day) / no PEN (n=47) | ✘ | Mean follow-up (range): 40.7 (3-232) months | Relapse: CDAI>150 + ↑CDAI>70 / hospitalisation with acute intestinal complications | Relapse rates: **48%** ≥1,200 kcal/day PEN group, **64%** <1,200 kcal/day PEN group (≥1,200 kcal/day vs <1,200 kcal/day: P=0.047) |
| **Takagi et al., 2006^51^** Parallel RCT | 50% PEN (900-1,200 kcal/day) + 5-ASA (2250-3000 mg/day) (n=26) | Unrestricted diet + 5-ASA (2250-3000 mg/day) (n=25) | Mean follow-up: 11.9 months | Remission: CDAI<150 Relapse: CDAI>200 / need for induction therapy | Relapse rates: **34.6% (9/26)** PEN group, 64% (16/25) control group (PEN vs control multivariate HR (95% CI): 0.4 (0.16-0.98)) |
| **Yamamoto et al., 2007a^52^** Open-label controlled trial | 50% PEN (1,200-1,800 kcal/day) overnight via NGT + low-fat diet (20-30 g/day) diet + 5-ASA (3000 mg/day) (n=20) | Unrestricted diet + 5-ASA (3000 mg/day) (n=20) | 1 year | Remission: CDAI<150  Relapse: CDAI≥150 | Relapse rates: **25% (5/20)** PEN group, 65% (13/20) control group (PEN vs control: P=0.03) CDAI mean (SE): no change from **101 (6.3)** PEN group (NS), ↑from 92 (4.8) control group (P=0.0001) (post-treatment values not presented) Albumin mean (SE): no change from **3.8 (0.1) g/dL** PEN group (NS), no change from 3.7 (0.1) g/dL control group (NS); albumin higher in PEN group vs control group after 1 year (P=0.04) (post-treatment values not presented) Endoscopic scores mean (SE): from **0.9 (0.2) to 1.3 (0.3)** PEN group, from 0.9 (0.2) to 2.0 (0.3) control group (PEN vs control: P=0.04) Mucosal IL-1β: no change PEN group (NS), ↑ control group (P=0.02) (PEN vs control, after 6 months: P=0.045, after 1 year: P=0.03) (values not presented) Mucosal IL-6: no change PEN group (NS), ↑ control group (P=0.002) (PEN vs control, after 6 months: P=0.03, after 1 year: P=0.04) (values not presented) Mucosal TNF-α: no change PEN group (NS), ↑ control group (P=0.001) (PEN vs control, after 6 months: P=0.04, after 1 year: P=0.04) (values not presented) |
| **Triantafillidis et al., 2010^56^** Parallel RCT | PEN at 500 kcal/day (n=36) | Unrestricted diet + 5-ASA (800 mg 3 times/day) (n=30) | 6 months | Relapse: CDAI>150 / ↑CDAI>60 | Relapse rates: **16.7% (6/36)** PEN group, 23.3% (7/30) control group, after 3 months (PEN vs control: NS); **30.5% (11/36)** PEN group, 40% (12/30) control group, after 6 months (PEN vs control: NS) |
| **Hanai et al., 2012^57^** Parallel RCT | PEN at ≥900 kcal/day (n=32) | 6-MP (0.5–1.5 mg/kg/day) (n=30)  No treatment (control group) (n=33) | 2 years | Relapse: CDAI≥200 / need for additional medication | Relapse rates: **25% (8/32)** PEN group, 16.7% (5/30) 6-MP group, 39.4% (13/33) control group, after 1 year (PEN vs control: NS; PEN vs 6-MP: NS); **37.5% (12/32)**, PEN group, 23.3% (7/30) 6-MP group, 63.6% (21/33) control group, after 2 years (PEN vs control: P=0.0348; PEN vs 6-MP: NS) |
| **Duncan et al., 2014^61^** Retrospective | 25% PEN + AZA (n=9)  25% PEN (n=6) | AZA (n=20)  No treatment (control group, n=13) | 1 year | Remission: PGA (based on combination of inflammatory markers, stool frequency, general well-being, and weight gain) | Remission rates: **89% (8/9)** PEN+AZA group, **100% (6/6)** PEN group, 80% (16/20) AZA group, 15% (2/13) control group, after 6 months (PEN vs control: P=0.003; PEN and PEN+AZA vs AZA and control: P=0.02); **67% (6/9)** PEN+AZA group, **50% (3/6)** PEN group, 65% (13/20) AZA group, 15% (2/13) control group, after 1 year (PEN and PEN+AZA vs AZA and control: NS) |
| **Konno et al., 2015^63^** Retrospective | PEN at ≥30kcal/kg/day + low-residue low-fat diet (<20g/day) (n=58) | ✘ | Median (range) follow-up: 50 (12-216) months | Remission: PCDAI<10 + SES-CD<2 | Relapse rate: **43.1% (25/58)** |
| **Kim et al., 2015^64^** Retrospective | PEN at 400-800 kcal/day (n=58) | ✘ | Mean (range): 16 (3-60) months | Remission: PCDAI<10 Relapse: PCDAI>10 + symptoms recurrence | Remission rates: **55% (32/58)**, using ITT analysis; **73% (32/43)**, using per protocol analysis; **67% (18/27)** after 1 year; **52% (7/13)** after 2 years |
| **Schulman et al., 2017^70^** Retrospective | 50% PEN (n=42) | Unrestricted diet (n=45) | Median (IQR) follow-up: 40 (25.5-79) months PEN group; 63 (52-87) months control group | Remission: PGA (based on clinical judgement) | Length of remission median (range): **6 (0-36) months** PEN group, 6 (0-45) months control group (PEN vs control: NS) wPCDAI median (IQR): from **5.0 (0-12.5)** **to 7.5 (0-17.5)** after 3 months, **to 3.8 (0-13.8)** after 1 year, **to 17.5 (10-20)** after 2 years, **to 17.5 (5-17.5)** after 3 years, PEN group; from 10.0 (7.5-17.5) to 11.3 (5-27.5) after 5 months, to 10.0 (0-20) after 1 year, to 10.0 (2.5-23.8) after 2 years, to 15.0 (0-21.9) after 3 years, control group (PEN vs control, baseline: P=0.005, after 3/5 months: NS, after 1 year: NS, after 2 years: NS; after 3 years: NS) CRP median (range): from **5 (0-107) to 6.4 (0-180) mg/L** after 3 months, PEN group; from 6.5 (1-48) to 5 (1-84) mg/L after 5 months, control group (PEN vs control, baseline: P=0.006, after 3/5 months: NS) Albumin mean (SD): from **42 (5)** **to 39 (5) g/L** after 3 months, PEN group; from 42 (4) to 42 (3) g/L after 5 months, control group (PEN vs control, baseline: NS, after 3/5 months: P=0.007) |
| **Gavin et al., 2018^66^** Retrospective | 30% PEN (n=58) | Unrestricted diet (n=44) | Median (range): 4 (1-12) months | Relapse: PGA (based on combination of inflammatory markers, stool output, anthropometry, and general wellbeing) | Relapse rates: **36% (21/58)** PEN group, 48% (21/44) control group, after 6 months (PEN vs control: NS); **78% (45/58)** PEN group, 77% (34/44) control group, after 12 months (PEN vs control: NS) |
| **Logan et al., 2018^67^** Prospective observational study with a control group | 20-25% PEN (n=29) | Unrestricted diet (n=12) | 1 year | Remission: wPCDAI<12.5 Relapse: need for induction therapy | Results for PEN group presented for 9 patients who were consuming PEN based on prescription and dietary records, and results for control group presented for 32 patients who did not. Relapse rates: **56% (5/9)** PEN group, 31% (10/32) control group, after 6 months (PEN vs control: NS); **89% (8/9)** PEN group, 66% (21/32) control group, after 12 months (PEN vs control: NS) FC median (IQR): **651 (271-1,781) μg/g** PEN group, 1238 (749-2,102) μg/g control group, 17 days post-EEN (PEN vs control: P=0.049) |
| **Bruckner et al., 2020^22^** Open-label controlled trial | 25% PEN (n=22) | Unrestricted diet (n=19) | 1 year | Relapse: wPCDAI≥12.5 + ↑CRP / ↑ESR / ↑FC / need for additional medication | Relapse rates: **18.2% (4/22)** PEN group, 21.1% (4/19) control group (PEN vs control: NS) |
| **Davidson et al., 2022^69^** Retrospective | 80% PEN for first 3-6 months + 70% PEN for another 6-12 months (n=10) | Thiopurines / methotrexate (n=17) | 1 year follow-up | Remission: sPCDAI<10 | Remission rates: **83% (5/6)** PEN group, 59% (10/17) control group, after 6 months (PEN vs control: NS); **67% (2/3)** PEN group, 64% (7/11) control group, after 1 year (PEN vs control: NS) Albumin median (IQR): **4.2 (4.0-4.4)** **g/dL** PEN group, 4.1 (4.0-4.5) g/dL control group, after 6 months (PEN vs control: NS); **4.2 (3.8-4.4)** **g/dL** PEN group, 4.2 (4.0-4.4) g/dL control group, after 1 year (PEN vs control: NS) White blood cell count median (IQR): **9.3 (7.8-9.9)** **10^3^/µL** PEN group, 7.1 (5.5-8.2) 10^3^/µL control group, after 6 months (PEN vs control: P=0.021); **7.2 (6.3-8.1)** **10^3^/µL** PEN group, 6.9 (6.3-8.7) 10^3^/µL control group, after 1 year (PEN vs control: NS) Haemoglobin median (IQR): **12.3 (12.0-12.6)** **g/dL** PEN group, 13.2 (12.3-14.5) g/dL control group, after 6 months (PEN vs control: NS); **12.8 (12.3-13.3)** **g/dL** PEN group, 14.3 (13.6-14.8) g/dL control group, after 1 years (PEN vs control: NS) CRP median (IQR): **0.7 (0.4-1.5)** **mg/dL** PEN group, 0.8 (0.4-1.3) mg/dL control group, after 6 months (PEN vs control: NS); **0.4 (0.4-1.4) mg/dL** PEN group, 0.8 (0.4-2.4) mg/dL control group, after 1 year (PEN vs control: NS) ESR median (IQR): **10.0 (5.0-26.3) mm/h** PEN group, 10.0 (4.0-20.0) mm/h control group, after 6 months (PEN vs control: NS); **5.0 (2.5-16.2)** **mm/h** PEN group, 9.0 (3.0-17.0) mm/h control group, after 1 year (PEN vs control: NS) FC median (IQR): **398 (310-507) μg/g** PEN group, 414 (253-465) μg/g control group, after 6 months (PEN vs control: NS); **371 (371-371)** **μg/g** PEN group, 335 (110-434) μg/g control group, after 1 year (PEN vs control: NS) |

*Duration is presented for the duration of intervention unless stated otherwise.

Abbreviations used: 5-ASA: Aminosalicylates; 6-MP: 6-Mercaptopurine; 95% CI: 95% Confidence Intervals; AZA: Azathioprine; CDAI: Crohn’s Disease Activity Index; CRP: C-Reactive Protein; ESR: Erythrocyte Sedimentation Rate; FC: Faecal Calprotectin; GI: Gastrointestinal; IL-1β: Interleukin 1 Beta; IL-6: Interleukin 6; IQR: Interquartile Range; ITT: Intention-to-treat; NGT: Nasogastric Tube; NS: Not Significant; PCDAI: Paediatric Crohn’s Disease Activity Index; PEN: Partial Enteral Nutrition; PGA: Physician Global Assessment; RCT: Randomised Control Trial; SD: Standard Deviation; SE: Standard Error; SEM: Standard Error of Mean; SES-CD: Simplified Endoscopic Score for Crohn’s Disease; TPN: Total Parenteral Nutrition; sPCDAI: Short Paediatric Crohn’s Disease Activity; wPCDAI: Weighted Paediatric Crohn’s Disease Activity Index.

**Supplementary Table 5:** Summary of study results investigating the clinical efficacy of partial enteral nutrition as maintenance treatment in patients with Crohn’s disease in remission after surgical gut resection. Results are presented at the end of intervention/follow-up duration unless states otherwise. Values in bold represent data for patients receiving partial enteral nutrition.

| **Study reference** | **Intervention description** | **Comparison description** | **Duration*** | **Post-operative recurrence criteria** | **Results** |
| --- | --- | --- | --- | --- | --- |
| **Esaki et al., 2005^49^** Retrospective | PEN at >1,200 kcal/day (n=24)  PEN at ≤1,200 kcal/day / no PEN (n=16) | ✘ | Mean (range) follow-up: 2.6 (0.5-6.9) years | Clinical recurrence: CDAI>150 + ↑CDAI>100 1-month post-surgery/hospitalisation with acute intestinal obstruction | Clinical recurrence: **46% (11/24)** >1,200 kcal/day PEN group, **75% (12/16)** ≤1,200 kcal/day PEN group (>1,200 kcal/day vs ≤1,200 kcal/day: P=0.017) |
| **Yamamoto et al., 2007b^53^** Open-label controlled trial | 50% PEN at 1,200-1,800 kcal/day overnight via NGT + low-fat diet (20-30 g/day) + 5-ASA (3000 mg/day) (n=20) | Unrestricted diet + 5-ASA (3000 mg/day) (n=20) | 1 year | Clinical recurrence: CDAI≥150 Endoscopic recurrence: RS≥i2 | Clinical recurrence: **5% (1/20)** PEN group, 35% (7/20) control group (PEN vs control: P=0.048) Endoscopic recurrence: **25% (5/20)** PEN group, 40% (8/20) control group, after 6 months (PEN vs control: NS); **30% (6/20)** PEN group, 70% (14/20) control group, after 1 year (PEN vs control: P=0.027) |
| **Yamamoto et al., 2013^55^** Secondary outcome analysis to open-label controlled trial  (Yamamoto et al., 2007b) | 50% PEN at 1,200-1,800 kcal/day overnight via NGT + low-fat diet (20-30 g/day) + 5-ASA (3000 mg/day) (n=20) | Unrestricted diet + 5-ASA (3000 mg/day) (n=20) | 5 years | Clinical recurrence: CDAI≥200 Endoscopic recurrence: RS≥i2 Surgical recurrence | Clinical recurrence: **30% (6/20)** PEN group, 60% (12/20) control group (PEN vs control: NS) Endoscopic recurrence: **56% (9/16)** PEN group, 82% (14/17) control group (PEN vs control: NS) Surgical recurrence: **5% (1/20)** PEN group, 25% (5/20) control group (PEN vs control: NS) |
| **Ohara et al., 2017^65^** Retrospective | PEN at ≥900 kcal/day (n=21)  PEN at <900 kcal/day / no PEN (n=17) | ✘ | Median (range) follow-up: 52 (16-99) months | Clinical recurrence: CDAI ≥220 Endoscopic recurrence: RS≥i2 | Clinical recurrence: **5% (1/21)** ≥900 kcal/day PEN group, **24% (4/17)** <900 kcal/day PEN group (≥900 kcal/day vs <900 kcal/day: NS) Endoscopic recurrence: **14% (3/21)** ≥900 kcal/day PEN group, **41% (7/17)** <900 kcal/day PEN group (≥900 kcal/day vs <900 kcal/day: NS) Clinical remission duration: longer in ≥900 kcal/day PEN group vs <900 kcal/day PEN group (P=0.019) (no values presented) Endoscopic remission duration median: **69 months** ≥900 kcal/day PEN group, **22 months** <900 kcal/day PEN group (≥900 kcal/day vs <900 kcal/day: P=0.021) |
| **Shinozaki et al., 2021^68^** Retrospective | PEN at ≥900 kcal/day (n=20)  PEN at <900 kcal/day) / no PEN (n=17) | ✘ | Median (range) follow-up: 1721 (106-7758) days | Surgical recurrence | Surgical remission duration median: **3920 days** ≥900 kcal/day PEN group, **2009 days** <900 kcal/day PEN group (≥900 kcal/day vs <900 kcal/day: P=0.011) |

*Duration is presented for the duration of intervention unless stated otherwise.

Abbreviations used: 5-ASA: Aminosalicylates; CDAI: Crohn’s Disease Activity Index; NGT: Nasogastric Tube; NS: Not Significant; PEN: Partial Enteral Nutrition; RS: Rutgeerts Score.

**Supplementary Table 6:** Summary of study results investigating the effect of partial enteral nutrition on nutritional outcomes of paediatric patients with Crohn’s disease. Results are presented at the end of intervention/follow-up duration unless states otherwise. Values in bold represent data for patients receiving partial enteral nutrition.

| **Study reference** | **Intervention description** | **Comparison description** | **Duration*** | **Results** |
| --- | --- | --- | --- | --- |
| **Aiges et al., 1989^45^** Open-label controlled trial | Supplementary PEN at 1,000-1,500 kcal/day overnight via NGT (n=8) | Unrestricted diet (n=6) | 1 year | Weight mean (range): ↑by **11.8 (6.9-14.4) kg** PEN group (P<0.001), ↑by 0.83 (-2.3-4.4) kg control group (PEN vs control: P<0.001) Height mean (range): ↑by **7 (3.0-10.5) cm** PEN group (P<0.0005), ↑by 1.5 (0.8-3.0) cm control group (PEN vs control: P<0.0005) Height velocity≥4 cm/year: **75% (6/8)** PEN group, 0% (0/6) control group Tanner stage progression to stage 2: **25% (2/8)** PEN group, 0% (0/6) control group |
| **Wilschanski et al., 1996^46^** Retrospective | 50-60% PEN overnight via NGT for 4-5 nights/week (n=28) | Unrestricted diet (n=19) | 1 year | Heigh velocity mean (SD): ↑from **3.2 (1.6) to 6.1 (4.2) cm/year** PEN group (P<0.001), from 3.8 (1.2) to 4.2 (4.5) cm/year control group (NS) |
| **Johnson et al., 2006^8^** Parallel RCT | 50% PEN (n=26) | EEN (n=24) | 6 weeks | Weight mean (95% CI): ↑by **3.0 (2-4) kg** PEN group (P=0.001), ↑by 3.6 (2.5-4.7) kg EEN group (P=0.001) (PEN vs EEN: NS) Weight/height z-score mean (95% CI): ↑by **0.5 (0.3-0.8)** PEN group (P<0.001), ↑by 0.7 (0.5-0.9) EEN group (P<0.001) (PEN vs EEN: NS) Mid-upper AC mean (95% CI): ↑by **9 (0-18) mm** PEN group (NS), ↑by 15 (8-22) mm EEN group (P<0.001) (PEN vs EEN: NS) TSF mean (95% CI): ↑by **1.3 (0.3-2.3) mm** PEN group (P=0.014), ↑by 1.3 (-0.2-2.8) mm EEN group (NS) (PEN vs EEN: NS) Subscapular skinfold mean (95% CI): ↑by **1.2 (0-2.4) mm** PEN group (P=0.04), ↑by 1.3 (0.6-2) mm EEN group (P=0.001) (PEN vs EEN: NS) |
| **Hartman et al., 2008^20^** Retrospective | 35-50% PEN with Modulen IBD (n=28)  35-50% PEN with Ensure Plus (n=18) | Unrestricted diet (n=18) | ~4.5-5.5 months intervention / follow-up | Weight z-score mean (SD): from **-1.7 (1.4) to -1.1 (1.3)** Modulen group (NS), from **-1.8 (1.1) to -1.2 (0.9)** Ensure Plus group (NS), from -1.2 (1.6) to -1 (1.4) control group (NS) Height z-score mean (SD): from **-1.2 (1.4) to -1 (1.2)** Modulen group (NS), from **-1 (0.9) to -0.9 (0.9)** Ensure Plus group (NS), from -1 (1.1) to -1 (1) control group (NS) BMI z-score mean (SD): ↑from **-1.3 (0.8) to -0.7 (0.7)** Modulen group (P=0.01), from **-1.8 (1.9) to -0.9 (1)** Ensure Plus group (NS), from -0.8 (1.5) to -0.6 (1.3) control group (NS) |
| **Gupta et al., 2013^26^** Retrospective | 80-90% PEN overnight via NGT (n=43) | ✘ | 8-12 weeks | Weight mean: from **36.4 to 41 kg** (P value not presented) Weight z-score mean: ↑from **-1.45 to -0.85** (P<0.03) Height mean: from **144.5 to 146.7 cm** in patients who continued on PEN as maintenance treatment (n=29); from 144.5 to 151.3 cm in patients who did not continue on PEN (n=14), after 6 months (P values not presented) Height z-score mean: ↑from **-1.1 to -0.9** in patients who continued on PEN as maintenance treatment (n=29, P<0.005); from -1.08 to -0.93 in patients who did not continue on PEN (n=14, P value not presented), after 6 months |
| **Kang et al., 2015^29^** Open-label controlled trial | PEN at 20kcal/kg/day (n=17) | Unrestricted diet (n=17) | 4 weeks | Results presented after 1 year from study enrolment.  Weight mean (SD): ↑by **10.7 (2.5) kg** PEN group, ↑by 7.4 (4.2) kg control group (PEN vs control: P<0.001) Height mean (SD): ↑by **4.6 (1.7) cm** PEN group; ↑by 3.4 (1.9) cm control group (PEN vs control: P=0.031) Haemoglobin mean (SD): ↑by **5.7 (0.4) g/dL** PEN group, ↑by 4.4 (0.6) g/dL control group (PEN vs control: P=0.012) Transferrin saturation mean (SD): ↑by **21.7 (2.9) %** PEN group, ↑by 16.4 (3.4) % control group (PEN vs control: P=0.033) Ferritin mean (SD): ↑by **134.8 (12.7) ng/mL** PEN group, ↑by 108.6 (21.1) ng/mL control group (PEN vs control: P=0.007) Prealbumin mean (SD): ↑by **117.6 (30.4) mg/L** PEN group, ↑by 82.7 (42.1) mg/L control group (PEN vs control: P<0.001)  Albumin mean (SD): ↑by **0.9 (0.2) g/dL** PEN group, ↑by 0.7 (0.4) g/dL control group (PEN vs control: P=0.041)  Zinc mean (SD): ↑by **45.6 (12.4) μg/dL** PEN group, ↑by 26.7 (21.6) μg/dL control group (PEN vs control: P<0.001) Calcium mean (SD): ↑by **0.8 (0.2) mg/dL** PEN group, ↑by 0.6 (0.3) md/dL control group (PEN vs control: P=0.018) Magnesium mean (SD): ↑by **0.3 (0.1) mmol/L** PEN group, ↑by 0.2 (0.1) mmol/L control group (PEN vs control: P=0.049)  Phosphorus mean (SD): ↑by **0.6 (0.1) mg/dL** PEN group, ↑by 0.5 (0.2) mg/dL control group (PEN vs control: P=0.038)  Vitamin A mean (SD): ↑by **0.48 (0.17) mg/L** PEN group, ↑by 0.32 (0.21) mg/L control group (PEN vs control: P=0.007)  Vitamin B12 mean (SD): ↑by **297.4 (27.1) pg/mL** PEN group, ↑by 197.6 (42.8) pg/mL control group (PEN vs control: P<0.001) Vitamin E mean (SD): ↑by **7.2 (1.4) mg/L** PEN group, ↑by 6.3 (3.1) mg/L control group (PEN vs control: P<0.001)  Folate mean (SD): ↑by **8.4 (1.3) ng/mL** PEN group, ↑by 6.9 (3.4) ng/mL control group (PEN vs control: P<0.001)  25-OH-vitamin D mean (SD): ↑by **4.3 (0.6) ng/mL** PEN group, ↑by 3.8 (1.3) ng/mL control group (PEN vs control: P<0.001) |
| **Lee et al., 2015^30^** Prospective observational study with a control group | 50% PEN (n=16) | EEN (n=22)  Anti-TNFα (n=52) | 8 weeks | Weight z-score mean (SD): ↑by **0.8 (0.7)** PEN group (P<0.05), ↑by 0.7 (0.6) EEN group (P<0.05), ↑by 0.4 (0.4) anti-TNFα group (P<0.05) (PEN vs anti-TNFα: P=0.04; PEN vs EEN: NS) BMI z-score mean (SD): ↑by **1.2 (1.4)** PEN group (NS), ↑by 1.1 (1.0) EEN group (NS), ↑by 0.5 (0.6) anti-TNFα group (NS) (PEN vs anti-TNFα: NS; PEN vs EEN: NS) |
| **Schulman et al., 2017^70^** Retrospective | 50% PEN (n=42) | Unrestricted diet (n=45) | Median (IQR) follow-up: 40 (25.5-79) months PEN group; 63 (52-87) months control group | Weight mean (SD): from **42.1 (13.2) to 45.1 (13.9) kg** after 6 months, PEN group; from 52.2 (19.6) to 55.5 (21.3) kg after 8 months, control group (PEN vs control, baseline: P=0.005, after 6/8months: P=0.011)  Height mean (SD): from **1.5 (0.2) to 1.5 (0.2)** **m** after 6 months, PEN group; from 1.6 (0.2) to 1.6 (0.2) m after 8 months (PEN vs control, baseline: NS; after 6/8months: NS) BMI mean (SD): from **18.1 (4.2) to 18.8 (2.9) kg/m^2^** after 6 months, PEN group; from 20.4 (5.4) 8 to 21.3 (4.8) kg/m^2^ after 8 months (PEN vs control, baseline: P=0.03; after 6/8months: P=0.006) |
| **Gavin et al., 2018^66^** Retrospective | 30% PEN (n=58) | Unrestricted diet (n=44) | Median (range): 4 (1-12) months | BMI z-score median (range): ↓from **-0.6 (-3.2-1.5) to -0.4 (-3.2-2.1)** PEN group (P=0.04), from 0.1 (-2.2-3.7) to -0.1 (-2.4-3.4) control group (NS) Height z-score median (range): from **-0.3 (-2.9-1.9) to -0.4 (-3.0-1.6)** PEN group (NS), from -0.3 (-2.4-2.1) to -0.3 (-2.5-1.9) control group (NS) |
| **Agin et al., 2019^34^** Retrospective | 50% PEN (n=16) | Unrestricted diet (n=19) | 8 weeks | Weight z-score mean (SD): from **-2.2 (1.6) to -1.2 (1.1)** after 1 week, **to -0.9 (1.4)** after 1 month, **to 0.1 (0.9)** after 3 months, **to 0.6 (0.8)** after 6 months, PEN group; from 1.3 (1.2) to -0.1 (1.7) after 1 week, to 0.1 (1.7) after 1 month, to 0.3 (1.8) after 3 months, to 0.6 (1.8) after 6 months, control group (PEN vs control, baseline: P=0.016, 1 week: P=0.014, 1 month: P=0.05, 3 months: NS, 6 months: NS) Height z-score mean (SD): from **-0.2 (1.2) to -0.1 (1.4)** after 1 week, **to -0.01 (1.5)** after 1 month, **to 0.1 (1.5)** after 3 months, **to 0.9 (0.8)** after 6 months, PEN group; from 0.1 (1.3) to 0.1 (1.3) after 1 week, to 0.2 (1.3) after 1 month, to 0.3 (1.3) after 3 months, to 0.5 (1.2) after 6 months, control group (PEN vs control, baseline: NS, 1 week: NS, 1 month: NS, 3 months: NS, 6 months: NS) Total protein mean (SD): from **6.6 (0.8) to 6.7 (0.7)** after 1 week, **to 6.7 (0.6)** after 1 month, **to 6.9 (0.7)** after 3 months, **to 6.8 (0.3) g/dL** after 6 months, PEN group; from 6.8 (0.7) to 6.9 (0.7) after 1 week, to 6.9 (0.4) after 1 month, to 7 (0.7) after 3 months, to 7.2 (0.4) g/dL after 6 months, control group (PEN vs control, baseline: NS, 1 week: NS, 1 month: NS, 3 months: NS, 6 months: P=0.014) |
| **Levine et al., 2019^10^** Parallel RCT | 50% PEN + CDED for first 6 weeks, 25% PEN + CDED for another 6 weeks (n=40) | EEN for first 6 weeks, 25% PEN for another 6 weeks (n=38) | 12 weeks | Weight z-score mean (SD): ↑from **-0.9 (1.2) to -0.6 (1.1)** PEN+CDED group (P<0.001), ↑from -0.9 (1.2) to -0.6 (1.1) EEN group (P<0.001), after 6 weeks (CDED+PEN vs EEN: NS) |
| **Bruckner et al., 2020^22^** Open-label controlled trial | 25% PEN (n=22) | Unrestricted diet (n=19) | 1 year | Height z-score mean (SD): ↑by **0.2 (0.3)** PEN group, by ↑0.1 (0.2) control group (PEN vs control: NS); by ↑**0.5 (0.2)** PEN group, by ↑0.15 (0.16) control group, in children with baseline Tanner stage 1-3 (n=20) (PEN vs control: P=0.005)  BMI z-score mean (SD): ↑by **0.3 (0.4)** PEN group, ↑by 0.1 (0.4) control group (PEN vs control: NS); ↑by **0.4 (0.3)** PEN group, ↑by 0.2 (0.4) control group, in children with baseline Tanner stage 1-3 (n=20) (PEN vs control: NS)  TrbD4 z-score mean (SD): ↓by **0.2 (1.1)** PEN group, ↑by 0.2 (0.7) control group (PEN vs control: NS)  CtD65 z-score mean (SD): ↑by **0.3 (0.8)** PEN group, ↓by 0.1 (0.7) control group (PEN vs control: NS)  CTth65 z-score mean (SD): ↑by **0.02 (0.5)** PEN group, ↑by 0.1 (0.7) control group (PEN vs control: NS)  MuscleCSA65 z-score mean (SD): ↑by **0.2 (0.6)** PEN group, ↓by 0.1 (0.7) control group (PEN vs control: NS)  MuscleCSA65^height^ z-score mean (SD): ↑by **0.4 (0.7)** PEN group, ↑by 0.1 (0.6) control group (PEN vs control: NS)  HGS z-score mean (SD): ↑by **0.1 (0.9)** PEN group, ↓by 0.1 (0.7) control group (PEN vs control: NS) |
| **Urlep et al., 2019^37^** Open-label controlled trial | 75% PEN + AID-CD (n=12) | EEN (n=13) | 6 weeks | Weight mean (SE): ↑from **49.7 (4.3) to 49.9 (4.2) kg** PEN+AID-CD group (NS), ↑from 45.4 (6.5) to 46.2 (6.1) kg EEN group (NS) (PEN+AID-CD vs EEN: NS) BMI mean (SE): ↑from **19.8 (1.0) to 19.9 (0.9) kg/m^2^** PEN+AID-CD group (NS), ↑from 18.9 (1.5) to 18.9 (1.4) kg/m^2^ EEN group (NS) (PEN+AID-CD vs EEN: NS) |
| **Niseteo et al., 2021^38^** Retrospective | EEN for first 1-2 weeks, 50% PEN + CDED for another 6 weeks (n=16)  50% PEN + CDED for 6 weeks (n=4) | EEN for 6-8 weeks (n=41) | 6-8 weeks | Combined results for EEN+PEN+CDED and PEN+CDED. Weight median (range): ↑by **1.3 (-3.1 to 5.3) kg** PEN+CDED groups, ↓by 0.7 (-7.0 to 10.00) kg EEN group (PEN+CDED vs EEN: P=0.002) BMI z-score median (range): ↑by **0.2 (-0.4 to 1.0)** PEN+CDED groups, ↓by 0.3 (-1.1 to 1.7) EEN group (PEN+CDED vs EEN: P=0.001) |
| **Jijón Andrade et al., 2023^43^** Retrospective | 50% PEN + CDED for first 6 weeks, 25% PEN + CDED for another 6 weeks, CDED for last 12 weeks (n=15: treatment-naïve (n=9), with LOR to biologics (n=6)) | ✘ | 24 weeks | Weight z-score: no changes (values not presented)  Iron median: ↑by **7.1 umol/L** in treatment-naïve patients (P=0.003), no changes in patients with LOR to biologics (values not presented) |

*Duration is presented for the duration of intervention unless stated otherwise.

Abbreviations used: 95% CI: 95% Confidence Intervals; AC: Arm Circumference; AID-CD: Anti-Inflammatory Diet for Crohn’s Disease; BMI: Body Mass Index; CDED: Crohn’s Disease Exclusion Diet; CtD65: Cortical Density; CTth65: Cortical Thickness; EEN: Exclusive Enteral Nutrition; HGS: Handgrip Strength; IQR: Interquartile Range; LOR: Loss of Response; muscleCSA65: Muscle Cross-sectional Area; muscleCSA65^height^: Muscle Cross-sectional Area corrected for height; NGT: Nasogastric Tube; NS: Not Significant; PEN: Partial Enteral Nutrition; pQCT: Peripheral Quantitative Computed Tomography; RCT: Randomised Control Trial; SD: Standard Deviation; SE: Standard Error; TNF; TNFα: Tumour Necrosis Factor Alpha; TrbD4: Trabecular Density; TSF – Triceps Skinfold.

**Supplementary Table 7:** Summary of study results investigating the effect of partial enteral nutrition on nutritional outcomes of adult patients with Crohn’s disease. Results are presented at the end of intervention/follow-up duration unless states otherwise. Values in bold represent data for patients receiving partial enteral nutrition.

| **Study reference** | **Intervention description** | **Comparison description** | **Duration*** | **Results** |
| --- | --- | --- | --- | --- |
| **Harries et al., 1983^71^** Crossover RCT | Supplementary PEN to achieve total 3000 kcal/day (n=28) | Unrestricted diet (n=28) | 2 months for each trial | Weight mean (SE): ↑by **3.3 (0.7) kg** PEN group (P<0.001), ↑by 2.9 (1.1) kg control group (P<0.05)  Mid-arm circumference mean (SE): ↑by **1.7 (0.5) cm** PEN group (P<0.01), ↑by 0.8 (0.5) cm control group (NS) Mid-arm muscle circumference mean (SE): ↑by **1.2 (0.4) cm** PEN group (P<0.05), ↑by 0.7 (0.3) cm control group (NS) Total skinfold thickness mean (SE): ↑by **7.4 (2.4) mm** PEN group (P<0.01), ↑by 2.3 (2.8) mm control group  Albumin mean (SE): ↑by **0.5 (0.5) g/L** PEN group (NS), ↑by 1.9 (0.7) g/L control group (P<0.05) Prealbumin mean (SE): ↑by **2.1 (0.8) mg/dL** PEN group (P<0.05), ↑by 3.7 (1.0) mg/dL control group (P<0.01) Creatinine height index mean (SE): ↑by **15.1 (9.9)** PEN group (NS), ↑by 7.1 (4.2) control group (NS) |
| **Imes et al., 1986^72^** Parallel RCT | Group 1: Supplementary PEN at 500 kcal/day for females, 750 kcal/day for males (n=34)  Group 2: Supplementary PEN at 500 kcal/day for females, 750 kcal/day for males + nutrition counselling (n=28) | Group 3: Unrestricted diet (n=31)  Group 4: Unrestricted diet + nutrition counselling (n=32) | 6 months | No differences in blood biomarkers of nutritional status between groups 1 and 2 vs groups 3 and 4. Patients categorised into 3 groups based on PEN use and compliance: no PEN group (patients not randomised to receive PEN - groups 3 and 4), PEN-compliant group (patients in groups 1 and 2 who complied with PEN), PEN-stopped (patients in groups 1 and 2 who did not comply with PEN). Low levels of serum vitamin B12: ↑from **0 to 19%** of patients PEN-stopped group (P<0.01), ↑from 7 to 20% of patients no PEN group (P<0.05) Low levels of WBC vitamin C: ↓from **44 to 22% of** patients PEN-stopped group (P<0.05) |
| **Verma et al., 2000^48^** Open-label controlled trial | 35-50% PEN (n=21) | Unrestricted diet (n=18) | 1 year | BMI mean (SEM): no change from **20.0 (2.2) kg/m^2^** PEN group (NS) (post-treatment values not presented), from 22.3 (1.3) to 23.1 (1.5) kg/m^2^ control group (NS) |
| **Verma et al., 2001^47^** Parallel RCT | 35-50% elemental PEN (n=19)  35-50% polymeric PEN (n=14) | ✘ | 1 year | Weight mean (SEM): no change from **62.4 (3.4) kg** elemental PEN group (NS), no change from **71.4 (7.7) kg** polymeric PEN group (NS) (post-treatment values not presented) BMI mean (SEM): no change from **21.8 (1.2) kg/m^2^** elemental PEN group (NS), no change from **24.4 (1.6) kg/m^2^** polymeric PEN group (NS) (post-treatment values not presented) |
| **Kuroki et al., 2003^73^** Cross-sectional | PEN mean (range): 1,384 (500-2,300) kcal/day (n=29) | Unrestricted diet (n=24) | Mean (SD; range): 1.5 (1.3; 0.1-5.3) years | Serum selenium mean (SD): **7.1 (3.5) μg/dl** PEN group, 10.2 (2.0) μg/dl control group (PEN vs control: P<0.0001) Selenium concentration correlated with PEN dose (r=-0.374, P<0.05) and duration (r=-0.433, P<0.05) |
| **Yamamoto et al., 2007a^52^** Open-label controlled trial | 50% PEN (1,200-1,800 kcal/day) overnight via NGT + low-fat diet (20-30 g/day) diet + 5-ASA (3000 mg/day) (n=20) | Unrestricted diet + 5-ASA (3000 mg/day) (n=20) | 1 year | Weight mean (SE): ↑from **51.1 (1.9) kg** after 6 months (P=0.004), no change from 6 months to 1 year (NS), PEN group; no change from 48.9 (1.7) kg after 6 months (NS), ↓from 6 months to 1 year (P=0.03), control group (PEN vs control, after 6 months: NS, after 1 year: NS) (post-treatment values not presented) BMI mean (SE): ↑from **19.2 (0.3) kg/m^2^** after 6 months PEN group (P=0.002), no change from 6 months to 1 year (NS), PEN group; no change from 19.1 (0.4) kg/m^2^ after 6 months (NS), ↓from 6 months to 1 year (P=0.02), control group (PEN vs control, after 6 months: NS, after 1 year: P=0.03) (post-treatment values not presented) |
| **Triantafillidis et al., 2010^56^** Parallel RCT | PEN at 500 kcal/day (n=36) | Unrestricted diet + 5-ASA (800 mg 3 times/day) (n=30) | 6 months | Weight mean (SD): ↑from **63.2 (13.5) to 65.1 (13.3) kg** PEN group (P=0.003), from 61.7 (13.8) to 63.2 (13.6) kg control group (NS) BMI mean (SD): ↑from **21.2 (4.5) to 21.9 (4.0) kg/m^2^** PEN group (P=0.002), from 21.2 (4.5) to 21.7 (4.0) kg/m^2^ control group (NS) Skinfold thickness mean (SD): ↑from **15.8 (7.5) to 16.9 (8.7) mm** PEN group (P=0.039), from 13.9 (7.5) to 15.1 (8.7) mm control group (NS) Mid arm circumference mean (SD): ↑from **28.0 (4.4) to 29.1 (4.7) cm** PEN group (P=0.004), from 26.7 (4.5) to 28.1 (4.8) cm control group (NS) Folic acid mean (SD): ↑from **6.3 (3.1) to 8.2 (3.9) ng/mL** PEN group (P=0.038), ↑from 5.9 (3.0) to 6.3 (3.4) ng/mL control group (P=0.030) Ferritin mean (SD): ↑from **60.6 (49) to 69.9 (35) μg/L** PEN group (P=0.019), ↑from 52.6 (59) to 61 (55) μg/L control group (P=0.038)  HDL mean (SD): ↑from **47.4 (10.3) to 51.1 (9.8) mg/dL** PEN group (P=0.003), from 49.5 (9.7) to 50.1 (10.3) mg/dL control group (NS) LDL mean (SD): ↓from **77.5 (30) to 68.1 (29) mg/dL** PEN group (P=0.002), from 81.5 (24) to 79.7 (26) mg/dL control group (NS) |
| **Wiese et al., 2011^23^** Open-label uncontrolled trial | PEN at 600 kcal/day with formula enriched with fish oil, prebiotics, and antioxidants (n=28) | ✘ | 4 months | 71.4% (20/28) patients completed the study (per protocol analysis): 50% (10/20) patients had serum EPA>2.0% indicating compliance (compliant group), and 50% (10/20) had EPA<2.0% indicating poor compliance (non-compliant group). BMI median (IQR): ↑by **0.8 (0.1-1.2) kg/m^2^** all patients (P=0.011), ↑by **0.9 (0.3-1.2) kg/m^2^** compliant group (P=0.01), ↑by 0.5 (-0.1-1.2) kg/m^2^ non-compliant group (NS) Fat mass median (IQR): ↑by **0.4 (-0.7-2.1) kg** all patients (NS), ↑by **1.7 (0.1-2.2) kg** compliant group (P=0.049), ↓by 0.4 (-0.7-1.0) kg non-compliant group (NS) Fat-free mass median (IQR): ↑by **1 (-0.3-1.8) kg** all patients (P=0.043), ↑by **1.1 (0.1-2) kg** compliant group (P=0.014), ↓by 0.2 (-0.7-1.4) kg non-compliant group (NS)  Body fat % median (IQR): ↑by **0.2 (-1-1.4) %** all patients (NS), ↑by **0.2 (-2.3-1.4) %** compliant group (NS), by 0 (-0.9-1) % non-compliant group (NS) T-score median (IQR): by **0 (0-0.1)** all patients (NS), ↑by **0.1 (0-0.4)** compliant group (NS), by 0 (-0.1-0.1) non-compliant group (NS) Serum 25-OH vitamin D median (IQR): ↑by **7.4 (3.5-11.4) ng/mL** all patients (P<0.001), ↑by **9.5 (3.8-13.3) ng/mL** compliant group (P=0.002), ↑by 4.4 (0.8-9.4) ng/mL non-compliant group (P=0.027) PTH median (IQR): ↓by **12 (-24.5-8) pg/mL** all patients (NS), ↓by **17 (-24-5) pg/mL** compliant group (NS), ↓by 2.5 (-25-15) pg/mL non-compliant group (NS)  Homocysteine median (IQR): ↓by **0.4 (-1.3-1.3) µmol/L** all patients (NS), ↓by **0.5 (-1.7-1.3) µmol/L** compliant group (NS), ↑by 0.2 (-1.0-1.3) µmol/L non-complaint group (NS) MMA median (IQR): ↑by **9 (-20- -48) nmol/L** all patients (NS), ↑by **6 (-20-268)** **nmol/L** compliant group (NS), ↑by 9 (-224-10) nmol/L non-compliant group (NS) |
| **Nakano et al., 2017^31^** Open-label uncontrolled trial | EEN for first 2 weeks + PEN (one small meal allowed) for next 6 weeks (n=13) | ✘ | 12 weeks | BMI mean (SD): ↑from **19.4 (3.3) to 20.5 (3.8) kg/m^2^** (P=0.009) Total protein mean (SD): ↑from **7.3 (1.0) to 7.6 (0.9) g/dL** (P=0.04)  Histidine median (IQR): ↑from **69.5 (55.6-79.2) to 76.9 (70.5-82.9) μmol/L** (P=0.012) Tryptophan median (IQR): ↑from **42.5 (35.4-55.2) to 53.7 (38.3-67.2) μmol/L** (P=0.012) Leucine median (IQR): from **96.3 (77.3-127.6) to 112.5 (89.6-126.1) μmol/L** (NS) Valine median (IQR): from **188.2 (154.5-233.1) to 231.0 (167.1-268.0) μmol/L** (NS) Methionine median (IQR): from **26.2 (21.1-39.3) to 31.7 (22.9-53.3) μmol/L** (NS) Isoleucine median (IQR): from **64.4 (53.6-84.2) to 69.8 (59.6-87.8) μmol/L** (NS)  Phenylalanine median (IQR): from **56.6 (41.5-78.9) to 63.4 (46.5-79.8)** **μmol/L** (NS) Threonine median (IQR): from **123.8 (101.2-145.1) to 118.9 (105.1-146.3) μmol/L** (NS) Lysine median (IQR): from **177.2 (148.0-224.6) to 175.7 (147.4-192.9)** **μmol/L** (NS) Essential amino acids median (IQR): from **835.9 (733.6-1037.4) to 982.1 (754.7-1086.9) μmol/L** (NS) |
| **Wall et al., 2018^33^** Open-label controlled trial | EEN for first 2 weeks + PEN (one small meal allowed) for next 6 weeks (n=13) | EEN (n=25) | 8 weeks | BMI median: from **25.2 to 24.7 kg/m^2^** PEN group (NS), ↑from 23.7 to 23.3 kg/m^2^ EEN group (P=0.01) (PEN vs EEN: NS) IGF-1 SDS median: ↑from **0.0 to 0.1** in all patients after 2 weeks (P=0.006), improvements sustained at week 8 in PEN and EEN groups (no values presented) (PEN vs EEN: NS) |
| **Ferreira et al., 2020^35^** Open-label controlled trial | 50% PEN with Nutren + nutrition counselling (n=13)  50% PEN with Modulen IBD + nutrition counselling (n=13) | Unrestricted diet + nutrition counselling (n=12) | 3 months | Weight mean (SD): ↑from **59.6 (3.8) to 62.6 (3.5) kg** Nutren group (P=0.01), ↑from **54 (2.9) to 60.6 (3.3) kg** Modulen group (P=0.02), from 67.9 (4.2) to 68.3 (4.9) kg control group (NS) (between-group*, baseline: P=0.04, after 3 months: NS)  BMI median (range): from **23.4 (13.8-27.5) to 24.3 (15.9-28.5) kg/m^2^** Nutren group (NS), from **19.4 (14.1-26.6) to 23.2 (18.4-29) kg/m^2^** Modulen group (NS), from 24.1 (15.8-38.4) to 23.3 (18-38.6) kg/m^2^ control group (NS) (between-group*, baseline: NS, after 3 months: NS) AbC mean (SD): from **82 (2.9) to 83.8 (2.6) cm** Nutren group (NS), from **77 (2.1) to 81.5 (2.3) cm** Modulen group (NS), from 87.6 (4.3) to 89.2 (4.8) cm control group (NS) (between-group*, baseline: NS, after 3 months: NS) AC median (range): ↑from **26.5 (19-32) to 28 (22-23) cm** Nutren group (P<0.01), ↑from **24.10 (19-33) to 27 (24-33) cm** Modulen group (P=0.04), from 26.5 (19-37) to 25 (23-37) cm control group (NS) (between-group*, baseline: NS, after 3 months: NS) TSF median (range): ↑from **8 (4.6-21.3) to 11 (5-23.6) mm** Nutren group (P<0.01), from **16 (3-25.5) to 18 (4.5-25.5) mm** Modulen group (NS), ↑from 11.5 (5-21.6) to 13.6 (4-26.6) mm control group (P=0.04) (between-group*, baseline: NS, after 3 months: NS) AMA mean (SD): from **35.5 (11.4) to 37 (9) cm^2^** Nutren group (NS), ↑from **27.6 (9.9) to 30.7 (9.9) cm^2^** Modulen group (P<0.01), from 31.4 (16.7) to 32.8 (13.9) cm^2^ control group (NS) (between-group*, baseline: NS, after 3 months: NS) HGS mean (SD): from **27.2 (3.2) to 29.2 (3.4) kgf** Nutren group (NS), from **25.3 (2.5) to 27.2 (3.3) kgf** Modulen group (NS), from 26.1 (2.4) to 27.1 (2.9) kgf control group (NS) (between-group*, baseline: NS, after 3 months: NS) SGA malnutrition risk: from **30.8% to 15.4%** patients Nutren group (NS), from **46.2% to 22.2%** patients Modulen group (NS), from 25% to 30% patients control group (NS) (between-group*, baseline: NS, after 3 months: NS)  *P-values from ANOVA/Kruskal-Wallis test, post-hoc p-values not presented |
| **Szczubełek et al., 2021^39^** Open-label uncontrolled trial | 50% PEN + CDED for first 6 weeks, 25% PEN + CDED for another 6 weeks (n=32) | ✘ | 12 weeks | Weight mean (SD): from **67.3 (16.4) to 67.9 (16.3)** after 6 weeks (NS), **to 67.2 (14.9) kg** after 12 weeks (vs baseline: NS; vs 6 weeks: NS)  BMI median (IQR): from **21.4 (19.2-24.7) to 21.5 (19.8-25.4)** after 6 weeks (NS), **to 21.2 (19.8-24.4) kg/m^2^** after 12 weeks (vs baseline: NS; vs 6 weeks: NS) Sodium mean (SD): from **140.9 (1.9) to 140.3 (2.0)** after 6 weeks (NS), **to 140.8 (1.4) mmol/L** after 12 weeks (vs baseline: NS; vs 6 weeks: NS) Potassium mean (SD): from **4.4 (0.3) to 4.4 (0.2)** after 6 weeks (NS), **to 4.3 (0.3) mmol/L** after 12 weeks (vs baseline: NS; vs 6 weeks: NS) Calcium mean (SD): ↑from **2.3 (0.1) to 2.4 (0.1)** after 6 weeks (P=0.002), **to 2.4 (0.1) mmol/L** after 12 weeks (vs baseline: NS; vs 6 weeks: NS) Iron mean (SD): from **74.9 (45.6) to 78.2 (38.0)** after 6 weeks (NS), **to 79.5 (40.7) µg/dL** after 12 weeks (vs baseline: NS; vs 6 weeks: NS) Ferritin median (IQR): from **61.0 (22.5-154.0) to 57.0 (24.5-113.3)** after 6 weeks (NS), **to 32.0 (12.5-80.5) ng/mL** after 12 weeks (vs baseline: NS; vs 6 weeks: NS)  Vitamin B12 mean (SD): ↑from **407.6 (224.1) to 528.6 (262.8)** after 6 weeks (P<0.001), **to 488.8 (249.6) pg/mL** after 12 weeks (vs baseline: P=0.012; vs 6 weeks: P=0.002) Vitamin D3 mean (SD): ↑from **26.3 (12.3) to 30.7 (12.9)** after 6 weeks (P=0.025), **to 28.6 (9.2) ng/mL** after 12 weeks (vs baseline: NS; vs 6 weeks: NS) Folic acid median (IQR): ↑from **5.8 (3.9-12.4) to 13.3 (10.3-15.7)** after 6 weeks (P=0.010), **to 11.7 (9.3-16.3) ng/mL** after 12 weeks (vs baseline: NS; vs 6 weeks: NS) Total protein mean (SD): from **7.2 (0.7) to 7.3 (0.8)** after 6 weeks (NS), **to 7.1 (0.9) g/dL** after 12 weeks (vs baseline: NS; vs 6 weeks: NS) |
| **Yanai et al., 2022^40^** Parallel RCT | PEN (1000 kcal/day) + CDED + vitamin D (2000 IU/day) supplementation for first 6 weeks, PEN (600 kcal/day) + CDED + calcium and vitamin D (2000 IU/day) supplementation for next 6 weeks, CDED + calcium and vitamin D (2000 IU/day) supplementation for last 12 weeks (n=19) | CDED + calcium and vitamin D (2000 IU/day) supplementation for first 6 weeks, CDED + calcium and vitamin D (2000 IU/day) supplementation for next 6 weeks, CDED + calcium and vitamin D (2000 IU/day) supplementation for last 12 weeks (n=21) | 24 weeks | Weight median (IQR): from **73.7 (60.1-85.4) to 76.4 (60.9-85.7)** after 6 weeks, **to 77.5 (67.0-83.2) kg** after 12 weeks, PEN+CDED group; from 70.0 (62.0-82.5) to 70.3 (60.2-82.6) after 6 weeks, to 72.0 (60.4-79.4) kg after 12 weeks, CDED group (PEN+CDED vs CDED:NS) BMI mean (SD): from **24.7 (6.2) to 26.0 (6.1) kg/m^2^** PEN+CDED group, from 25.9 (5.9) to 26.7 (5.9) CDED group, after 24 weeks (P values not presented) |
| **Ferreiro et al., 2021^42^** Open-label uncontrolled trial | Supplementary PEN provided at 200, 400 or 600 kcal/day (dose dependent on malnutrition severity) (n=144) | ✘ | 12 weeks | ↑BMI mean: from **20.2 to 21.1 kg/m^2^** (P=0.002) SGA nutritional status improvements: **83.8%** of patients: 54.6% from moderately malnourished to low risk of malnourishment, 26.1% from severely malnourished to low risk of malnourishment, 19.3% from severely malnourished to moderately malnourished (vs baseline: P<0.001) |

*Duration is presented for the duration of intervention unless stated otherwise.

Abbreviations used: 5-ASA: Aminosalicylates; AbC: Abdominal Circumference; AC: Arm Circumference; AMA: Arm Muscle Area; BMI: Body Mass Index; CDAI: Crohn’s Disease Activity Index; EEN: Exclusive Enteral Nutrition; EPA: Eicosapentaenoic Acid; HDL: High-Density Lipoprotein; HGS: Handgrip Strength; IGF-1: Insulin-Like Growth Factor-1; IQR: Interquartile Range; IU: International Units; LDL: Low-Density Lipoprotein; MMA: Methylmalonic Acid; NGT: Nasogastric Tube; NS: Not Significant; PEN: Partial Enteral Nutrition; PTH: Parathyroid Hormone; RBC: Red Blood Cell; RCT: Randomised Control Trial; SD: Standard Deviation; SDS: Standard Deviation Score; SE: Standard Error; SEM: Standard Error of Mean; SGA: Subjective Global Assessment; T-score: Total Body Estimated T score; TSF: Triceps Skinfold; WBC: White Blood Cell.

**Supplementary Table 8:** Summary of study results investigating the effect of partial enteral nutrition on quality of life of patients with Crohn’s disease. Results are presented at the end of intervention/follow-up duration unless states otherwise. Values in bold represent data for patients receiving partial enteral nutrition.

| **Study reference** | **Intervention description** | **Comparison description** | **Duration*** | **Results** |
| --- | --- | --- | --- | --- |
| **Kuriyama et al., 2009^74^** Cross-sectional | PEN (median (range): 900 (300-2,700 kcal/day) (n=95) | Unrestricted diet (n=31) | No information | IBDQ total score: higher in PEN group vs control group, in patients with disease duration ≥10 years (P=0.009), but not in patients with disease duration <10 years (NS) (values not presented) IBDQ bowel symptoms: higher in PEN group vs control group, in patients with disease duration ≥10 years (P=0.0033), but not in patients with disease duration <10 years (NS) (values not presented) IBDQ systemic symptoms: higher in PEN group vs control group, in patients with disease duration ≥10 years (P=0.0161), but not in patients with disease duration <10 years (NS) (values not presented) IBDQ emotional function: no differences between PEN vs control group, in patients with disease duration ≥10 years (NS), and in patients with disease duration <10 years (NS) IBDQ social function: no differences between PEN vs control group, in patients with disease duration ≥10 years (NS), and in patients with disease duration <10 years (NS) |
| **Takagi et al., 2009^75^** Secondary outcome analysis to parallel RCT (Takagi et al., 2006) | 50% PEN (900-1,200 kcal/day) + 5-ASA (2250-3000 mg/day) (n=26) | Unrestricted diet + 5-ASA (2250-3000 mg/day) (n=25) | Mean follow-up: 11.9 months | IBDQ total score (95% CI): from **164.5 (151.5-177.5) to 178.5 (162.5-194.5)** PEN group (NS), from 171.5 (159.7-183.3) to 171.9 (158.2-185.6) control group (NS) |
| **Wiese et al., 2011^23^** Open-label uncontrolled trial | PEN at 600 kcal/day with formula enriched with fish oil, prebiotics, and antioxidants (n=28) | ✘ | 4 months | 71.4% (20/28) patients completed the study (per protocol analysis): 50% (10/20) patients had serum EPA>2.0% indicating compliance (compliant group), and 50% (10/20) had EPA<2.0% indicating poor compliance (non-compliant group). IBDQ total score median (IQR): ↑by **23 (-1-47)** all patients (P=0.016), ↑by **41.4 (23.1-47)** compliant group (P=0.002), ↓by 1 (-9.9-9) non-compliant group (NS) |
| **Lee et al., 2015^30^** Prospective observational study with a control group | 50% PEN (n=16) | EEN (n=22)  Anti-TNFα (n=52) | 8 weeks | IMPACT-III total score mean (SD): ↑by **6.2 (9.6)** PEN group (P<0.05), ↑by 11.0 (12.1) EEN group (P<0.05), ↑by 10.5 (13.8) anti-TNFα group (P<0.05) (PEN vs EEN: NS, PEN vs anti-TNFα: NS) IMPACT-III bowel symptoms subscore mean (SD): ↑by **5.1 (13.5)** PEN group (NS) ↑by 14.4 (22.8) EEN group (P<0.05), ↑by 14.4 (20.3) anti-TNFα group (P<0.05) (PEN vs anti-TNFα: P=0.03, PEN vs EEN: NS) IMPACT-III systemic symptoms subscore mean (SD): ↑by **13.0 (23.4)** PEN group (P<0.05), ↑by 33.0 (27.3) EEN group (P<0.05), ↑by 21.6 (26.1) anti-TNFα group (P<0.05) (PEN vs anti-TNFα: NS, PEN vs EEN: P=0.03) IMPACT-III emotional function subscore mean (SD): ↑by **8.6 (13.6)** PEN group (P<0.05), ↑by 9.4 (17.4) EEN group (P<0.05), ↑by 10.4 (16.4) anti-TNFα group (P<0.05) (PEN vs anti-TNFα: P=0.01, PEN vs EEN: NS) IMPACT-III social function subscore mean (SD): ↑by **3.8 (7.5)** PEN group (NS), ↑by 6.0 (12.5) EEN group (P<0.05), ↑by 8.0 (12.0) anti-TNFα group (P<0.05) (PEN vs anti-TNFα: NS, PEN vs EEN: NS) IMPACT-III body image subscore mean (SD): ↑by **4.7 (12.9)** PEN group (NS), ↑by 12.9 (13.8) EEN group (P<0.05), ↑by 2.7 (16.6) anti-TNFα group (NS) (PEN vs anti-TNFα: NS, PEN vs EEN: NS) IMPACT-III treatment subscore mean (SD): ↑by **7.3 (16.1)** PEN group (NS), ↑by 3.0 (15.3) EEN group (NS), ↑by 7.5 (18.7) anti-TNFα group (P<0.05) (PEN vs anti-TNFα: NS, PEN vs EEN: NS) |
| **Szczubełek et al., 2021^39^** Open-label uncontrolled trial | 50% PEN + CDED for first 6 weeks, 25% PEN + CDED for another 6 weeks (n=32) | ✘ | 12 weeks | IBDQ total score mean (SD): ↑from **125.4 (36.6) to 172.3 (35.0)** after 6 weeks (P<0.001), **to 177.6 (31.2)** after 12 weeks (vs baseline: P<0.001; vs 6 weeks: NS) |

*Duration is presented for the duration of intervention unless stated otherwise.

Abbreviations used: 5-ASA: Aminosalicylates; 95% CI: 95% Confidence Intervals; CDED: Crohn’s Disease Exclusion Diet; EEN: Exclusive Enteral Nutrition; EPA: Eicosapentaenoic Acid; IBDQ: Inflammatory Bowel Disease Questionnaire; IQR: Interquartile Range; NS: Not Significant; PEN: Partial Enteral Nutrition; RCT: Randomised Control Trial; SD: Standard Deviation; TNFα: Tumour Necrosis Factor Alpha.
